# Synopsis of the genus *Ponthieva* (Orchidaceae) in Colombia

**DOI:** 10.7717/peerj.6728

**Published:** 2019-04-11

**Authors:** Dariusz L. Szlachetko, Marta Kolanowska, Natalia Olędrzyńska

**Affiliations:** 1 Department of Plant Taxonomy and Nature Conservation, Faculty of Biology, University of Gdańsk, Gdańsk, Poland; 2 Department of Biodiversity Research, Global Change Research Institute AS CR, Brno, Czech Republic; 3 Department of Geobotany and Plant Ecology, Faculty of Biology and Environmental Protection, University of Lodz, Łódź, Poland

**Keywords:** Biodiversity, Colombia, Cranichidinae, Neotropics, Taxonomy

## Abstract

The Neotropical orchid genus *Ponthieva* R. Br. was established in 1813. The 70 representatives of this taxon are terrestrial, lithophytic, or epiphytic plants with basal, sessile, or petiolate leaves which are glabrous or pubescent. Their erect peduncle is pubescent and terminated by lax, few- to many-flowered raceme. Flowers are non-resupinate, with dissimilar sepals and asymmetrical petals which are adnate to the sides of the gynostemium. The lip is uppermost, fused to the lower part of the gynostemium. Here the synopsis of Colombian *Ponthieva* is presented. A list of national genus representatives includes 26 species, including one new species, *P. vallecaucana* Szlach. Kolan. & Olędrzyńska, sp. nov., discovered during this study. We did not confirm occurrence of four species reported in previous research. To facilitate process of identification of genus representatives, we divided *Ponthieva* into six morphological complexes based on vegetative and floral characters. The highest number of species was found in Magdalena and Cauca Valley montane forests. Lectotypes for *Ponthieva inaudita* and *P. mandonii* are designated. Morphological characteristics and illustrations of perianth segments of Colombian representatives of the genus are provided as well as a key for their identification.

## Introduction

The tropical area of South America is well known for its extraordinary biodiversity and high endemism level. The biological richness is a result of a heterogeneity of abiotic conditions as well as of a complex geological history (Amorim & Pires, 1996; Sigrist & Barros De Carvalho, 2009). Unfortunately, continuing habitat destruction and fragmentation may result in losses of biodiversity before full species variousness is known. The recent studies on threats to South American flora revealed that over 78% of 16,339 analyzed species have at least one population which is seriously threatened by the anthropogenic activities (Ramirez-Villegas, Jarvis & Touval, 2012). Moreover, about 13% of the evaluated species have up to 80% of their populations are at risk of extinction (Ramirez-Villegas, Jarvis & Touval, 2012). Taxonomic revisions and floras are the most important source of data for assessing the extinction risk of organisms (Von Staden, Raimondo & Dayaram, 2013).

The aim of this study was to provide comprehensive taxonomic revision of the orchid genus *Ponthieva* R. Br. in Colombia, which is a part of monographic series dedicated to the orchid flora of this country. *Ponthieva* was described by Brown (1813) based on *Neottia glandulosa* Sims (≡ *Ponthieva glandulosa* (Sims) R.Br.). The generic name was given in honor of Henri de Ponthieu, a French merchant in the Antilles. There is general consensus on both the generic separateness of *Ponthieva* and its classification within Cranichidinae (Pfitzer, 1887; Dressler, 1993; Szlachetko, 1995; Chase et al., 2015). Due to several distinguishing morphological traits, for example, petals being adnate to or decurrent on the gynostemium, lip fused with the lower half of the gynostemium (Dressler, 1993), short, massive gynostemium which is much dilated just below the receptive surface, the digitate rostellum and triangular, acute hamulus (Szlachetko & Rutkowski, 2000) also the specific composition of the genus was largely accepted by taxonomists (Schweinfurth, 1958; Garay, 1978; Dressler, 2003; Pupulin, 2002). The situation changed when Salazar et al. (2009) published results of genetic studies on Cranichidinae and Prescottiinae. According to these studies *Ponthieva* is polyphyletic with two clades containing representatives of this genus. Peculiarly, the first one included also *Baskervilla colombiana* Garay in a derived position within the group formed by *P. formosa* Schltr., *P. elata* Schltr., and *P. tuerckheimii* Schltr. The second clade included, besides *Ponthieva* species, also *Exalaria parviflora* (C. Presl) Garay & G.A. Romero and *Ocampoa mexicana* A. Rich. & Galeotti. Salazar et al. (2009) decided to lump both these genera in *Ponthieva* but in this concept the genus is morphologically poorly defined as there is no synapomorphy for such defined taxon. The separateness of *Exalaria* Garay & G.A. Romero and *Ocampoa* A. Rich. & Galeotti was recently discussed by Kolanowska & Szlachetko (2015) who considered Salazar et al. (2009) proposal as premature and suggested that further phylogenetic study is necessary before any nomenclatural action can be taken.

In the present study, we applied a concept of *Ponthieva* that includes neither *Exalaria* nor *Ocampoa* (Kolanowska & Szlachetko, 2015). The genus is defined by the basal leaves, the peduncle more or less pubescent, terminated by a few- to many-flowered raceme. The flowers are non-resupinate with more or less connate lateral sepals and asymmetrical petals which are adnate much above the base to the side of the column. The small lip is adnate to the lower part of the gynostemium. The footless gynostemium is short and very massive, distinctly dilated just below the receptive surface. Populations of *Ponthieva* are found growing epiphytically in mossy trunks and terrestrially in forest floor and along humid banks. The altitudinal range extends from near the sea level up to over 3,000 m a.s.l.

## Materials and Methods

A total of about 300 herbarium specimens deposited or borrowed from herbaria AMES, BM, CUVC, COL, E, F, FI, K, MO, NY, QCA, QCNE, P, PSO, RPSC, UGDA, US, USF, VALLE, and W were examined. Additionally, the electronic database of scanned herbarium specimens deposited in RENZ was used. Each studied specimen was photographed and the data from the label were taken. The leaves arrangement, shape, and size were examined together with the length of the scape and rachis. Additionally, the presence/absence and type of indumentum on the surface of scape and rachis were surveyed. The form and size of the floral bracts and ovaries as well as the perianth segments were studied using a stereomicroscope. Flowers of each specimen were studied after softening in boiling water. The examined specimens were compared with type material, diagnoses, and original illustrations of *Ponthieva* representatives. Herbaria acronyms are cited according to “Index Herbariorum” (Thiers, 2018).

Distribution maps were prepared using ArcGIS 9.3 (Esri, Redlands, CA, USA) and a digital elevation model. Only localities (100 records) which could be precisely georeferenced were used.

### Nomenclature

The electronic version of this article in portable document format will represent a published work according to the International Code of Nomenclature for algae, fungi, and plants (Turland et al., 2018), and hence the new names contained in the electronic version are effectively published under that Code from the electronic edition alone. In addition, new names contained in this work which have been issued with identifiers by The International Plant Names Index (IPNI) will eventually be made available to the Global Names Index. The IPNI Life Science Identifiers (LSIDs) can be resolved and the associated information viewed through any standard web browser by appending the LSID contained in this publication to the prefix “http://ipni.org/.” The online version of this work is archived and available from the following digital repositories: PeerJ, PubMed Central, and CLOCKSS.

## Results

In 1994 Ortiz Valdivieso (in Escobar, 1994) suspected “some fourteen species” of *Ponthieva* to occur in this country, while later (Ortiz Valdivieso & Uribe Vélez, 2007) he listed 17 species in the list of Colombian orchids. In the most recent catalogue of Colombian plants Bernal, Gradstein & Celis (2015) noted 15 species. The latter authors treated *P. elata* Schltr. as synonym of *P. diptera* Lindl. & Rchb. f., accepted broad concept of *P. racemosa* (Walter) C. Mohr (including *P. rostrata* Lindl.*, P. orchioides* Schltr., and *P. glandulosa* (Sims) R. Br.), and added to the list presented by Ortiz Valdivieso & Uribe Vélez (2007) three species: *P. microglossa* Schltr., *P. venusta* Schltr., and *P. fertilis* (F. Lehm. & Kraenzl.) Salazar (=*Exalaria parviflora*).

The current list of Colombian *Ponthieva* includes 26 species, including one new species, *P. vallecaucana* Szlach. Kolan. & Olędrzyńska, sp. nov., discovered during this study. We did not confirm occurrence of four species reported in previous research (Ortiz Valdivieso & Uribe Vélez, 2007; Bernal, Gradstein & Celis, 2015): *P. andicola* Rchb.f., *P. appendiculata* Schltr., *P. orchioides*, and *P. rostrata*. To facilitate process of identification of genus representatives we divided *Ponthieva* into six morphological groups based on vegetative and floral characters.

We were able to precisely locate distribution of populations representing 21 species and the information about occurrence of other national *Ponthieva* was imprecise and we were not able to situate their localities. Populations of mapped species occur in 11 ecoregions (Olson et al., 2001) in Colombia: Apure-Villavicencio dry forests, Cauca Valley dry forests, Cauca Valley montane forests, Cordillera Oriental montane forests, Guajira-Barranquilla xeric scrub, Magdalena Valley dry forests, Magdalena Valley montane forests, Northern Andean páramo, Northwestern Andean montane forests, Santa Marta montane forests, and Santa Marta páramo. The highest number of *Ponthieva* species was found in Magdalena Valley montane forests (11) and in Cauca Valley montane forests (9). The occurrence of four species *P. gracilis* Renz*, P. pubescens* (C. Presl) C. Schweinf., *P. racemosa*, and *P. elata* is restricted to dry forest ecoregions.

## Taxonomic Treatment

***Ponthieva*** R. Br.
Hort. Kew. ed.2 **5**: 199–200. 1813; TYPE: *Ponthieva glandulosa* (Sims) R.Br. (≡ *Neottia glandulosa* Sims).

**Figure 1:**
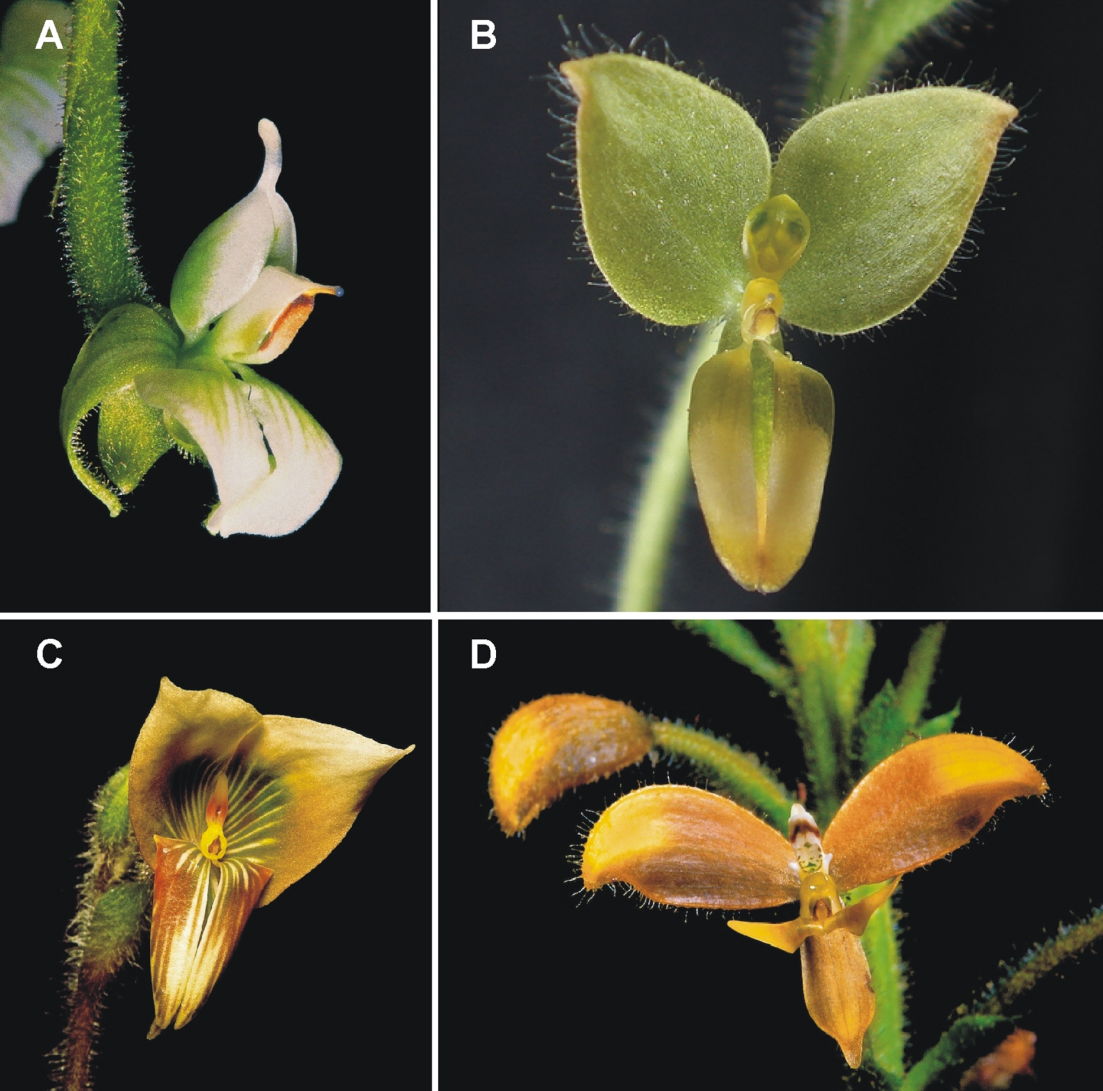
*Ponthieva* representatives. (A) *P. rostrata*, (B) *P. villosa*, (C) *P. andicola*, (D) *P. diptera*. Photos: (A, C, and D) Alexander Hirtz, (B) Ramiro Medina Trejo.

**Figure 2:**
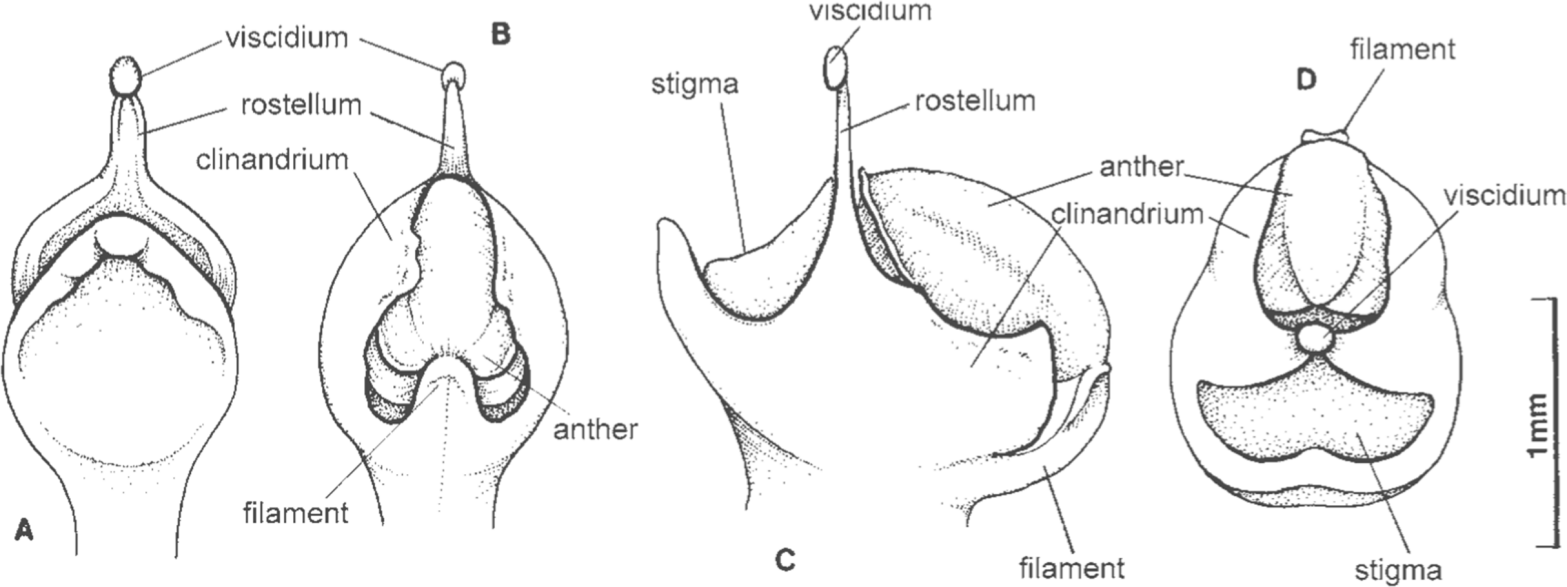
Gynostemium structure of *Ponthieva*. (A) Gynostemium, bottom view, (B) gynostemium, back view, (C) gynostemium, side view, (D) gynostemium, front view (Szlachetko & Rutkowski, 2000; based on *Ponthieva* cf. *lilacina*).

Plants terrestrial, lithophytic, or epiphytic. Roots fusiform, fleshy, fasciculate. Leaves usually basal, sessile or petiolate, erect or patent, glabrous or pubescent. Peduncle erect, pubescent, usually elongate, terminated by lax, few- to many-flowered raceme. Flowers non-resupinate, mostly inconspicuous, soft and delicate in texture. Sepals dissimilar; lateral sepals more or less connate basally. Petals asymmetrical, adnate much above the base to the sides of the gynostemium. Lip small, fleshy, uppermost, fused to the lower part of the gynostemium. Gynostemium short and very massive (except in *P. sprucei* Cogn.), sessile or prominently stalked, erect, greatly dilated just below the stigmatic surface. Column foot absent. Anther base situated well below the stigma base. Anther erect, oblong-ovate, motile. Pollinia 4, oblong-ovate, compact. Caudiculae formed by the elongated apical parts of pollinia. Staminodes wing-like, fused with the filament, or part of it, and with the stigma margins forming a prominent dorsal clinandrium, sometimes with thickened lower parts consisting of ballooned cells. Stigma hortizontal to subhortizontal, transversely elliptic, flat, but surrounded by thickened margins of lateral stigma lobes. Rostellum finger-like, erect, rather soft and fleshy, canaliculate. Viscidium single, detachable, fleshy, rather small. Hamulus small, triangular, acute, directed toward the anther. Rostellum truncate at the apex after the removal of pollinarium (Figs. 1 and 2).

The genus includes about 70 species distributed in the American tropics and subtropics, from Florida to northern Argentina, with the greatest diversity observed in the Andes.

## Key to the Groups

1. Leaves villose or pubescent2
1* Leaves glabrous3
2. Lip widest at the base, usually with complicated callus6. *Andicola* group
2* Lip widest at the apex, callus simple, basal5. *Maculata* group
3. Lip lamina narrow, much longer than wide3. *Diptera* group
3* Lip lamina more or less expanded, more or less as long as wide4
4. Lip lamina with dendritic venation4. *Mandoni* group
4* Lip lamina with parallel venation5
5. Lip lamina suborbicular to transversely elliptic1. *Rostrata* group
5* Lip lamina not as above, obcordate to triangular in general outline2. *Venusta* group

**1. *Rostrata* group**

Plants with sessile or petiolate, glabrous leaves, more or less pubescent scape and small, rather inconspicuous flowers, orbicular, elliptic to transversely elliptic lip lamina, unguiculate or sessile, rounded at apex with elongate apical lobule, papillose or glabrous on disc, occasionally with calli present on the claw only, the gynostemium shortly stalked, rather massive.

## Key to the Species

1. Lip claw with prominent calli*P. pubescens*
1* Lip claw ecallose, if any2
2. Margins of petals glabrous3
2* Margins of petals ciliolate4
3. Lip claw broadly ovate above narrow base, margins upcurved, foming a kind of channel; lamina 2.5 × 5 mm, transversely elliptic in general outline, apical projection 0.5 × 0.3 mm*P. castanedae*
3* Lip claw not as above; lamina 5.2 × 5.5 mm, obreniform in general outline, apical projection 1.2 × 0.5 mm*P. camargoi*
4. Lip sessile, disc sparsely glandulose-verrucose*P. orchioides*
4* Lip unguiculate, occasionally subsessile, disc papillose5
5. Petals with truncate base*P. rostrata*
5* Petals with auriculate base6
6. Lip disc with an obscure linear median crest*P. racemosa*
6* Lip disc without any crest*P. micromystax*

*Ponthieva orchioides* Schltr., Repert. Spec. Nov. Regni Veg. 15: 50. 1917. TYPE: Ecuador. *P. Mille 27A* (B†; lectotype, designated by Garay (1978: 219), AMES-00103552!—drawing).

**Figure 3:**
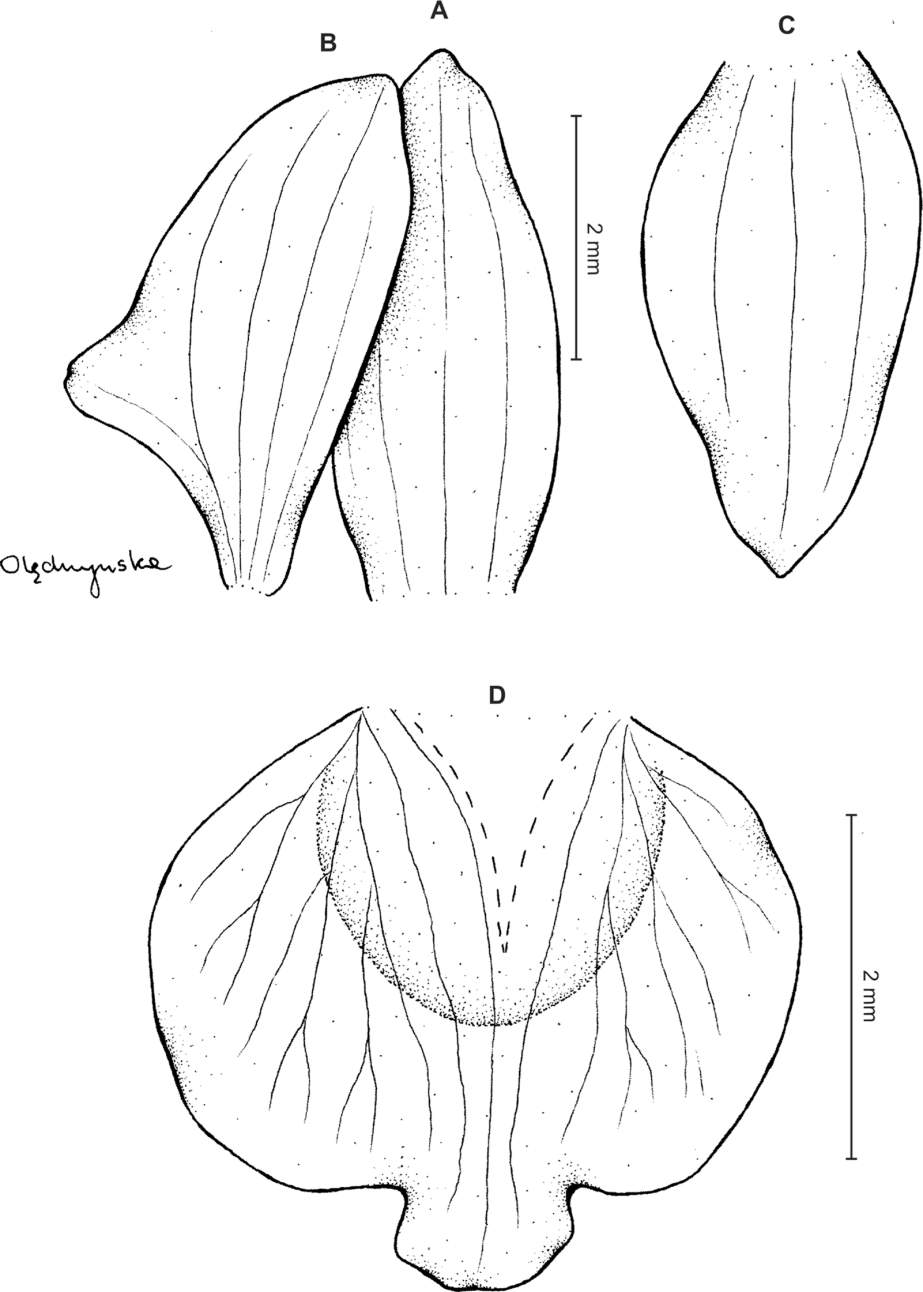
*Ponthieva orchioides* Schltr. (A) Dorsal sepal, (B) petal, (C) lateral sepal, (D) lip, front view. Drawn by N. Olędrzyńska from *Bonpland 6237*.

Plants up to 33 cm tall, erect. Leaves up to 6, basal, rosulate, subsessile; blade up to 7.5 × 2 cm, elliptic to oblanceolate, acute, tapering basally, glabrous. Peduncle erect with several loose-fitting, lanceolate, acuminate sheaths, glabrous basally, toward the rachis puberulent, terminating in a many-flowered, dense, elongate, cylindrical, raceme up to 10 cm long. Flowers small, greenish, sepals glandulose-puberulent externally. Floral bracts up to eight mm long, lanceolate, acuminate, glandulose-puberulent. Pedicellate ovary up to 10 mm long, glandulose-puberulent. Dorsal sepal up to 5 × 1.5 mm, narrowly ovate to ovate-elliptic, obtuse, 3-veined. Petals shortly unguiculate; blade up to 4.5 × 2.5 mm, apically coherent with the dorsal sepal, prominently bilobed, obliquely semirhombic to triangular-dolabriform in general outline with rounded angles, with the anterior margin minutely ciliolate, 2-veined. Lateral sepals up to five mm long and two mm wide, free to the base, obliquely ovate to elliptic-ovate, obtuse, glandulose-puberulent externally, 3-veined. Lip sessile, up to 3.5 × 3 mm, ecallose; lamina orbicular, concave to gibbose, apically 3-lobed; lateral lobes suborbicular; middle lobe 0.7–1 × 0.8–0.9 mm, suborbicular to ligulate-spathulate lobule; disc sparsely glandulose-verrucose, from the base to the middle with 3–7 veins, thin. Gynostemium 2.5 mm long in total, shortly stalked (Fig. 3).

*Ecology*: Terrestrial. Flowering in January.

*Distribution*: Ecuador, Colombia, Venezuela. The occurrence of this species in Colombia was reported by Ortiz Valdivieso & Uribe Vélez (2007). Alt. 1,700 m.

*Other materials examined*: ECUADOR. **Pichincha**: Pifo, *P. Mille 27a* (AMES!). VENEZUELA. **Miranda**: Hacienda San Blas, Los Teques, 1,700 m, 11 Jan. 1942, *F. Fernandez 82* (AMES!); Inter Maypure et San Fernando, *A. Bonpland 6237* (P!, UGDA!—drawing).

*Notes:* This species shares with *P. rostrata* a sessile or subsessile lip, but can be easily separated from the latter by its prominently bilobed petals, with somewhat upcurved lateral lobe. In other species of this group the lip is more or less unguiculate and petals are auriculate rather than having a lateral, upcurved lobe. The exception in the petals shape is *P. rostrata* with basally truncate petals but without prominent lobation.

*Ponthieva rostrata* Lindl., Ann. & Mag. Nat. Hist., ser. 1, 15: 385. 1845. TYPE: Ecuador. *K.T. Hartweg 1437* (lectotype, designated by Garay (1978: 224), K-L-000079986!; isolectotype, E!, FI-011847!, AMES-00103561!—drawing, UGDA!—drawing).
*Ponthieva ecuadorensis* Schltr., Rep. Sp. Nov. Regni Veg. 14: 117. 1915. TYPE: Ecuador. Pifo, *P. Mille 27* (B†; lectotype, designated by Garay (1978: 224), AMES-00103519!—drawing).
*Ponthieva rostrata* var*. spicata* Lindl., Ann. Mag. Nat. Hist. 15: 385. 1845. TYPE: Ecuador. Pichincha, near Quito, *K.T. Hartweg s.n*. (lectotype, designated by Garay (1978: 224), K; isolectotypes, P-00363512!, BM-000058544; NY!—photo of isotype).

**Figure 4:**
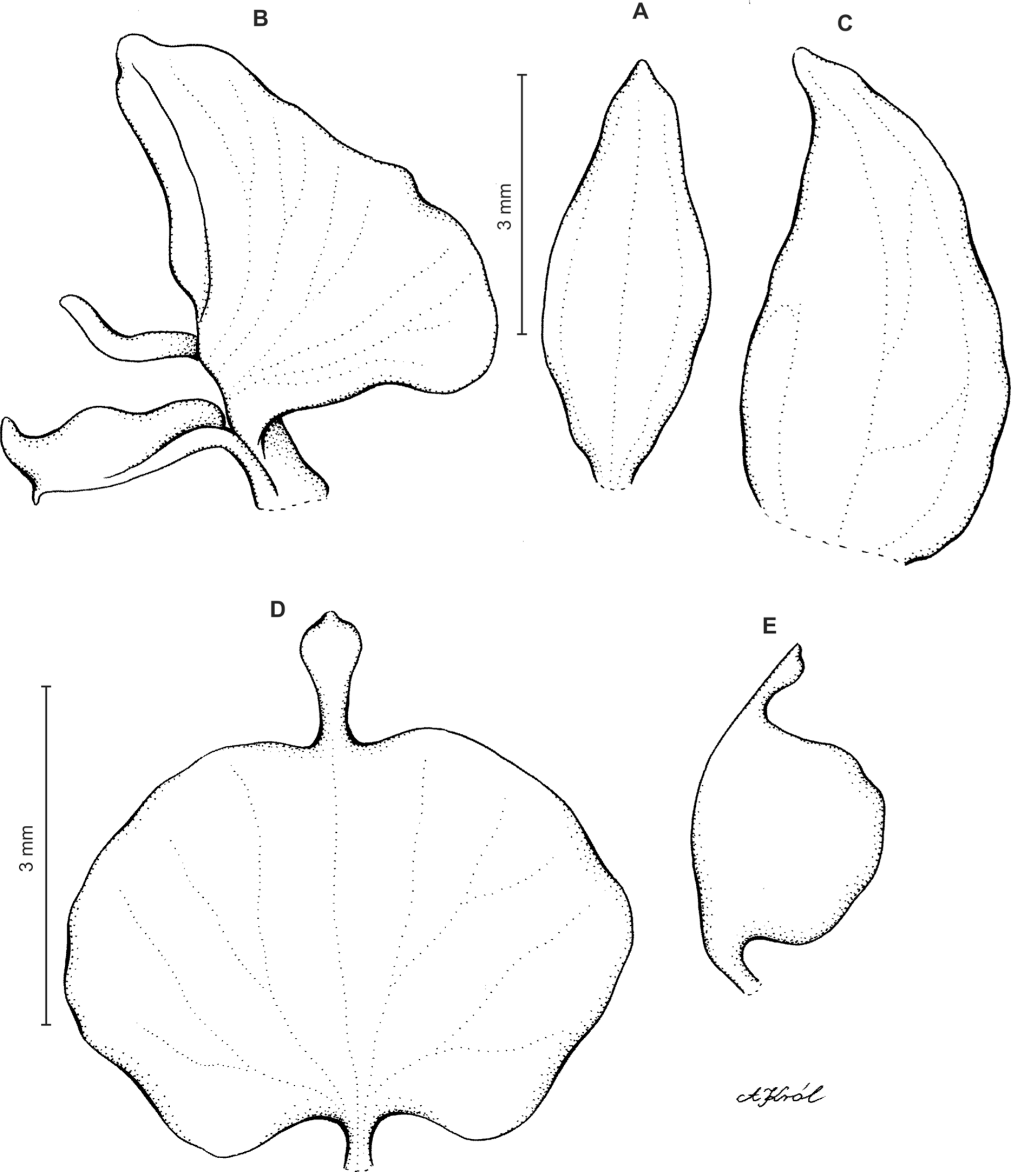
*Ponthieva rostrata* Lindl. (A) Dorsal sepal, (B) petal and lip, (C) lateral sepal, (D) lip, front view, (E) lip, side view. Drawn by A. Król from *Moritz 1104*.

**Figure 5:**
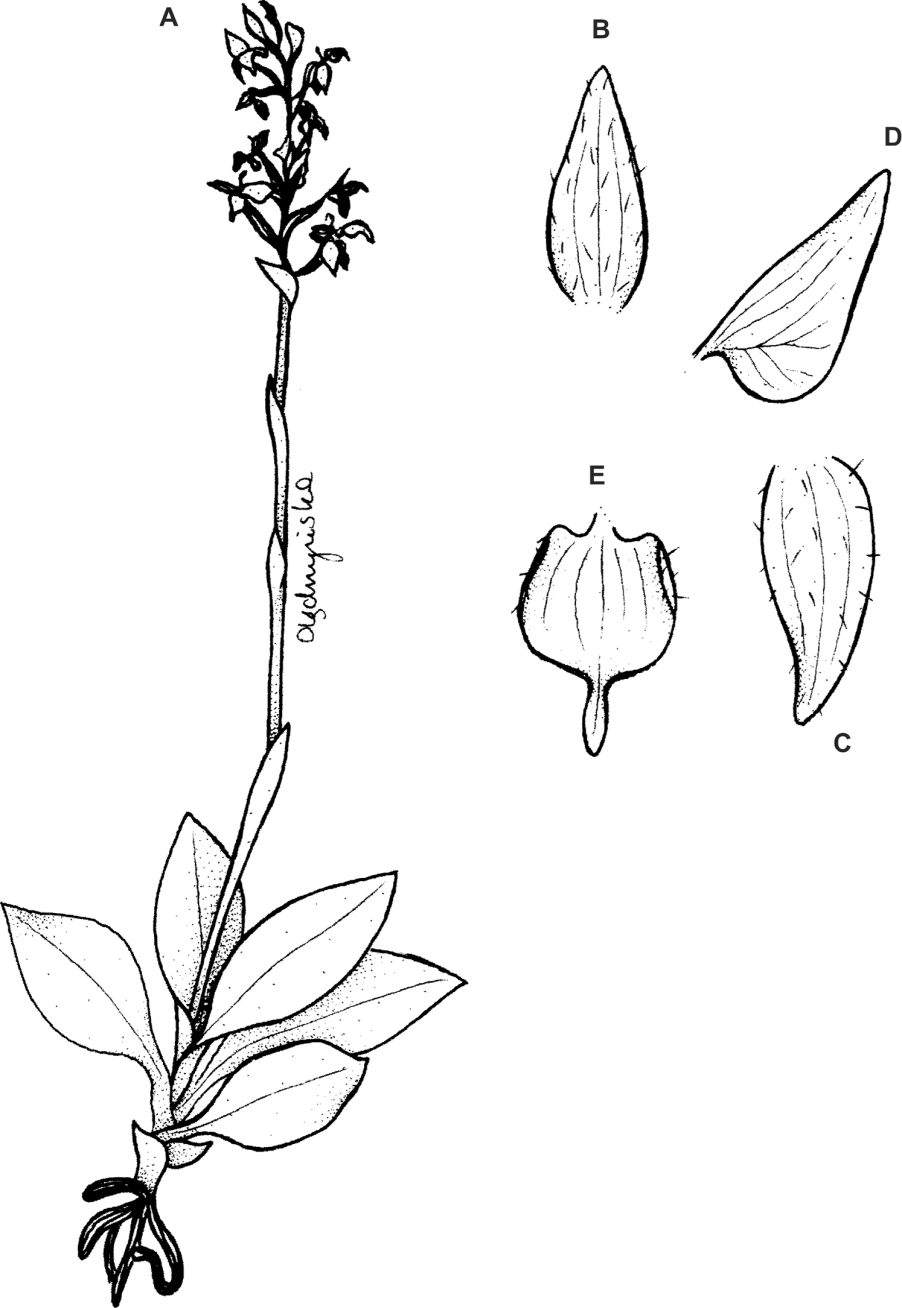
*Ponthieva rostrata* Lindl. (A) Habit, (B) dorsal sepal, (C) lateral sepal, (D) petal, (E) lip. Original illustration of *Ponthieva ecuadorensis* deposited in AMES redrawn by N. Olędrzyńska.

Plants 10–50 cm tall. Leaves 2–6, basal, petiolate; petiole l–4(7) cm long; blade up to nine cm long and 3.3 cm wide, commonly smaller, elliptic to lanceolate-elliptic, more or less oblique, acute to obtuse, glabrous. Peduncle erect, glandulose-puberulent above basal third, completely covered with approximate sheaths, terminated by a subdensely many-flowered, cylindrical raceme. Flowers greenish to greenish-white, sepals glandulose-pubescent externally. Floral bracts up to l4 mm long, ascending, ovate-lanceolate, acute to acuminate, glandulose-puberulent. Pedicellate ovary up to 22 mm long, glandulose-puberulent, ascending. Dorsal sepal up to seven mm long and 2.5 mm wide, lanceolate-elliptic to ovate-lanceolate, acute to subobtuse, 3-veined. Petals subsessile, blade up to seven mm long and four mm wide, obliquely triangular-ovate, more or less attenuate towards apex, rounded at distal end, basally truncate, apically connivent with dorsal sepal, margins distinctly but minutely ciliolate, 3-veined, veins branching. Lateral sepals up to eight mm long and three mm wide, free to the base, obliquely ovate to oblong ovate with more or less falcate apex, acute to subacuminate, 4-veined. Lip shortly unguiculate to subsessile; lamina up to five mm long and up to four mm wide when expanded, cymbiform, deeply concave, transversely elliptic in general outline, subcordate at base, truncate at apex, apically provided abruptly with a fleshy, conduplicate, elongate, deltoid or spathulate, acute apical lobule, disc densely papillose, margins thin, glabrous. Gynostemium up to five mm long in total, basaly shortly stalked (Figs. 4 and 5).

*Ecology*: Terrestrial. Flowering in February, May, and December.

*Distribution:* Peru, Ecuador, Colombia, Venezuela. The occurrence of this species in Colombia was reported by Ortiz Valdivieso & Uribe Vélez (2007) and from Peru by Zelenko & Bermúdez (2009).

*Other materials examined:* ECUADOR. **Pichincha**: *Sine loc*., *K.T. Hartweg 1437* (K-L!, W!); In Valle Chilo. Locis Columbia. Prope Quito, *K.T. Hartweg 1437* (P!, BM, NY!—photo); Near Quito, *W. Jameson 163* (E!, UGDA!—drawing). VENEZUELA. Mérida. 1844–45, *J.W.K. Moritz 1104* (W!, UGDA!—drawing). *Sine loc.*, Feb. 1880, *E. Klaboch s.n*. (W!).

*Notes*: *P. rostrata* can be distinguished from other species of this group by having subsessile lip with transversely elliptic lamina, devoid of any calli, except papillose protuberances on disc. The petals are relatively large, subsessile with minutely ciliolate margins, more or less triangular in general outline, attenuate towards apex, with rounded distal end and truncate base. It can be easily confused with *P. micromystax* and *P. camargoi* described below. Both species, however, have unguiculate lip and auriculate petals.

*Ponthieva micromystax* Kraenzl. *ex* Szlach. & Kolan., Ann. Bot. Fenn. 50: 394. 2013. TYPE: Colombia. *F.C. Lehmann s.n*. (holotype, W-80947!, UGDA!—drawing).

**Figure 6:**
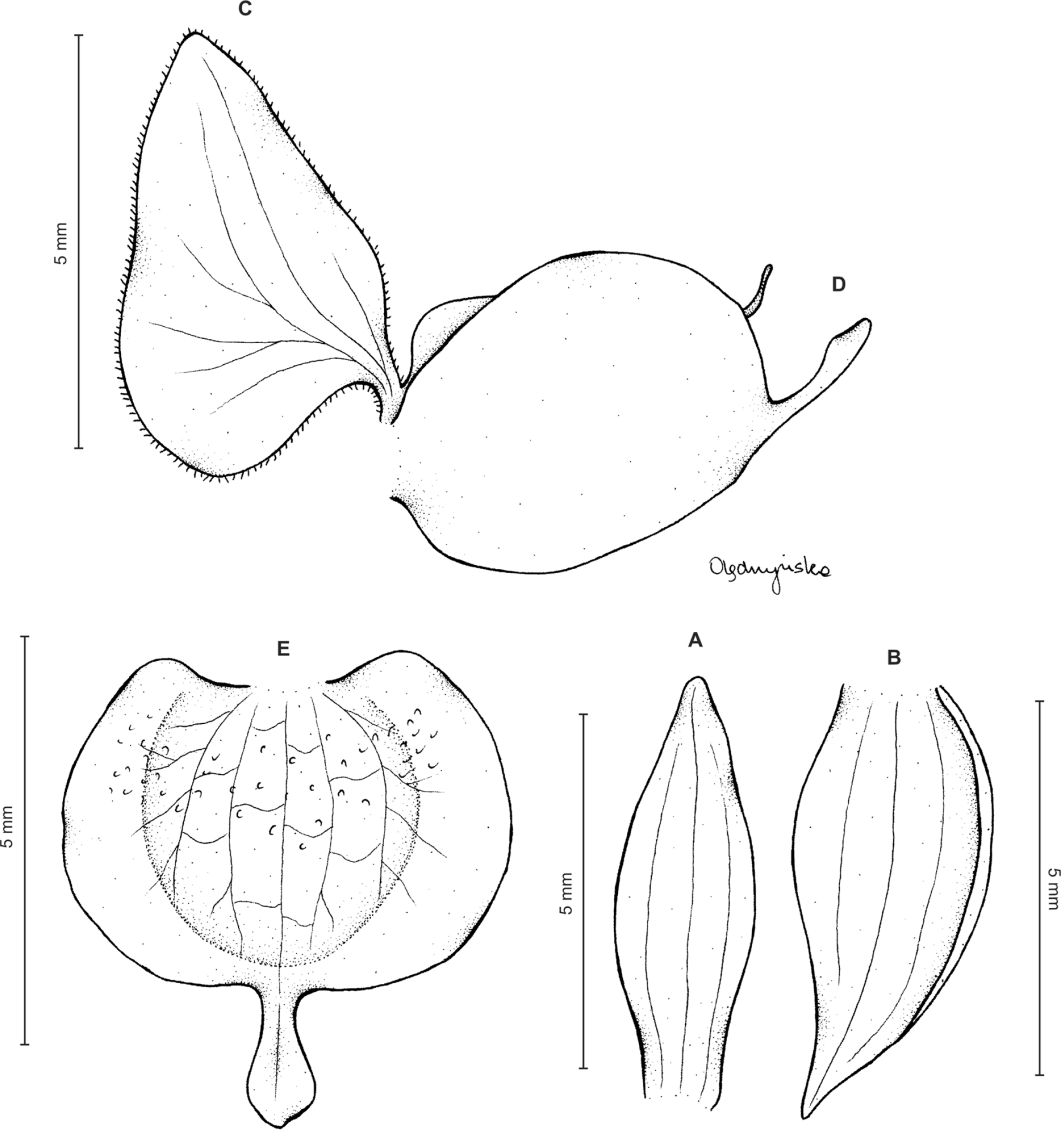
*Ponthieva micromystax* Kraenzl. *ex* Szlach. & Kolan. (A) Dorsal sepal, (B) lateral sepal, (C) petal, (D) lip, side view, (E) lip, front view. Drawn by N. Olędrzyńska from *Pennell 2536*.

Plants about 35 cm tall. Leaves 6, basal, subsessile; blade up to 4.5 cm long and 1.3 cm wide, oblong-lanceolate, acute to obtuse, glabrous. Peduncle erect, glandulose-puberulent above, enclothed by numerous sheaths, terminated by a fairly dense, many-flowered, cylindrical raceme. Flowers glandulose-pubescent externally. Floral bracts up to six mm long, ovate-lanceolate, acute to acuminate, glandulose-puberulent. Pedicellate ovary up to seven mm long, glandulose-puberulent. Dorsal sepal about 5.5 mm long and two mm wide, lanceolate-ovate, subobtuse, 3-veined. Petals up to five mm long and 3.5 mm wide, from shortly unguiculate base obliquely triangular-dolabriform, obtuse, rounded and auriculate at base, 1-veined, branching, margins ciliolate. Lateral sepals about six mm long and 2.5 mm wide, free to the base, obliquely oblong-ovate with a subfalcate apex, subobtuse, 3-veined, margins ciliolate. Lip distinctly, but shortly unguiculate; lamina 4.5–4.8 mm long and 2.8–3 mm wide, concave, suborbicular, cordate at base, apex with an elongate, linear lobule rounded at apex; disc 5-veined, nerves branching, papillose in basal half. Gynostemium ca three mm long (Fig. 6).

*Ecology*: Terrestrial, growing in grassy banks, meadows, and open hills. Flowering in January, May, September, October, and December.

*Distribution*: Ecuador, Colombia. Alt. 600–2,750 m.

**Figure 7:**
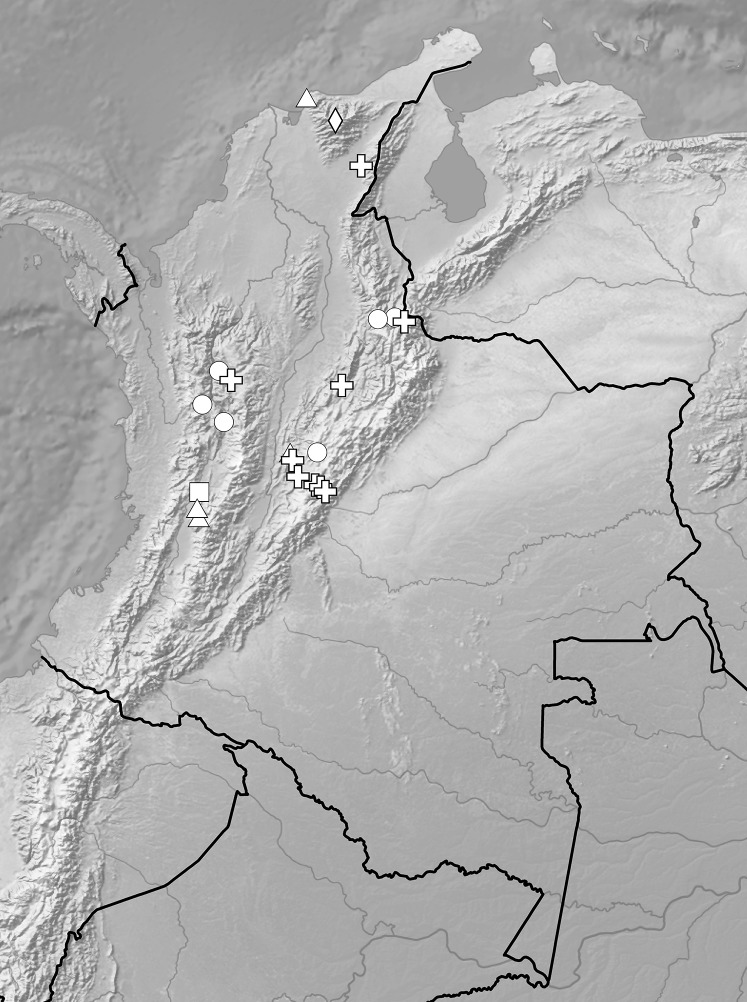
Distribution of representatives of *Rostrata*-group. *P. camargoi* (cross), *P. castanedae* (diamond), *P. micromystax* (cicrle), *P. pubescens* (square), *P. racemosa* (triangle). Map generated in ArcGis 9.3 (Esri, Redlands, CA, USA).

*Representative specimens:* COLOMBIA. **Antioquia**: 13 km al E de Bolivar, 2,700 m, 21 Jan. 1949, *J. Molina & F. Barkley 19An024* (US!, UGDA!—drawing); San Jerónimo, 1,500–1,800 m, Oct. 1884, *F.C. Lehmann 8166* (AMES!, NY!); Caramanta, Grassy bank, near Quebrada Arquia, 1,700–2,000 m, 19 Sep. 1922, *F.W. Pennell 10760* (AMES!, NY!, US!); **Cundinamarca**: Zipaquirá, in meadow, 2,650 m, 20–24 Oct. 1917, *F.W. Pennell 2536* (NY!, UGDA!—drawing); 50 miles N of Bogotá, 2,150 m, 26 May 1935, *A. Lawrance 846* (AMES!, UGDA!—drawing); **Magdalena**: Santa Marta, on the Onaca Estate, on rocks in damp forest, 1,200 m, 8 Dec. 1898, *H.H. Smith 2635* (AMES!, F!, NY!—partly damaged, US!, UGDA!—drawing). **Norte de Santander**: Valley of Rio Chitago, SW of Pamplona, Toledo up Rio Calaga, 1,800 m, 30 Oct. 1944, *N.C. Fassett 25990* (US!, UGDA!—drawing); Vicinity of California, 2,300 m, 11–27 Jan. 1927, *E. Killip & A. Smith 17058* (AMES!); **Valle del Cauca**: W Andes of Cali, 1,800 m, *F.C. Lehmann s.n*. (W!). **Unprecise locality**. Pedregal, *F.C. Lehmann BT75* (NY!) (Fig. 7).

*Other materials examined*: ECUADOR. **Pichincha**: Camino de Hospital, entre el Mauca Quito y el Sincholagua, 0°5′N 78°30′W, 2,400–2,750 m, 21 May 1988, *C. Cerón & M. Cerón 3774* (QCNE!, RPSC!, UGDA!—drawing).

*Notes*: *P. micromystax* can be confused with *P. rostrata*. In general the sepals and petals are similar in shape, but in *P. micromystax* they are prominently auriculate, and in *P. rostrata*—truncate at base. Also the lip morphology separates both species. The lip of *P. micromystax* is suborbicular usually with a linear, obtuse apical lobule, whereas in *P. rostrata* it is cymbiform-transversely elliptic with a deltoid or spathulate, acute apical lobule, widest just below the apex. Most valuable distinctive characters of *P. micromystax* are its subsessile, oblong-lanceolate leaves and the floral bracts almost as long as the ovaries. The leaf blade of *P. rostrata* is elliptic to lanceolate-elliptic, with a one to four cm long petiole, and the floral bracts are much shorter than ovaries. *Ponthieva camargoi* differs from *P. micromystax* in having 1.5 mm long unguiculate petals and lip.

*Ponthieva camargoi* Szlach. & Kolan., Pl. Syst. Evol. 299: 1672. 2013. TYPE: Colombia. *G. Huertas & L.A. Camargo G. 6971* (holotype, COL-6971!; UGDA!—drawing).

**Figure 8:**
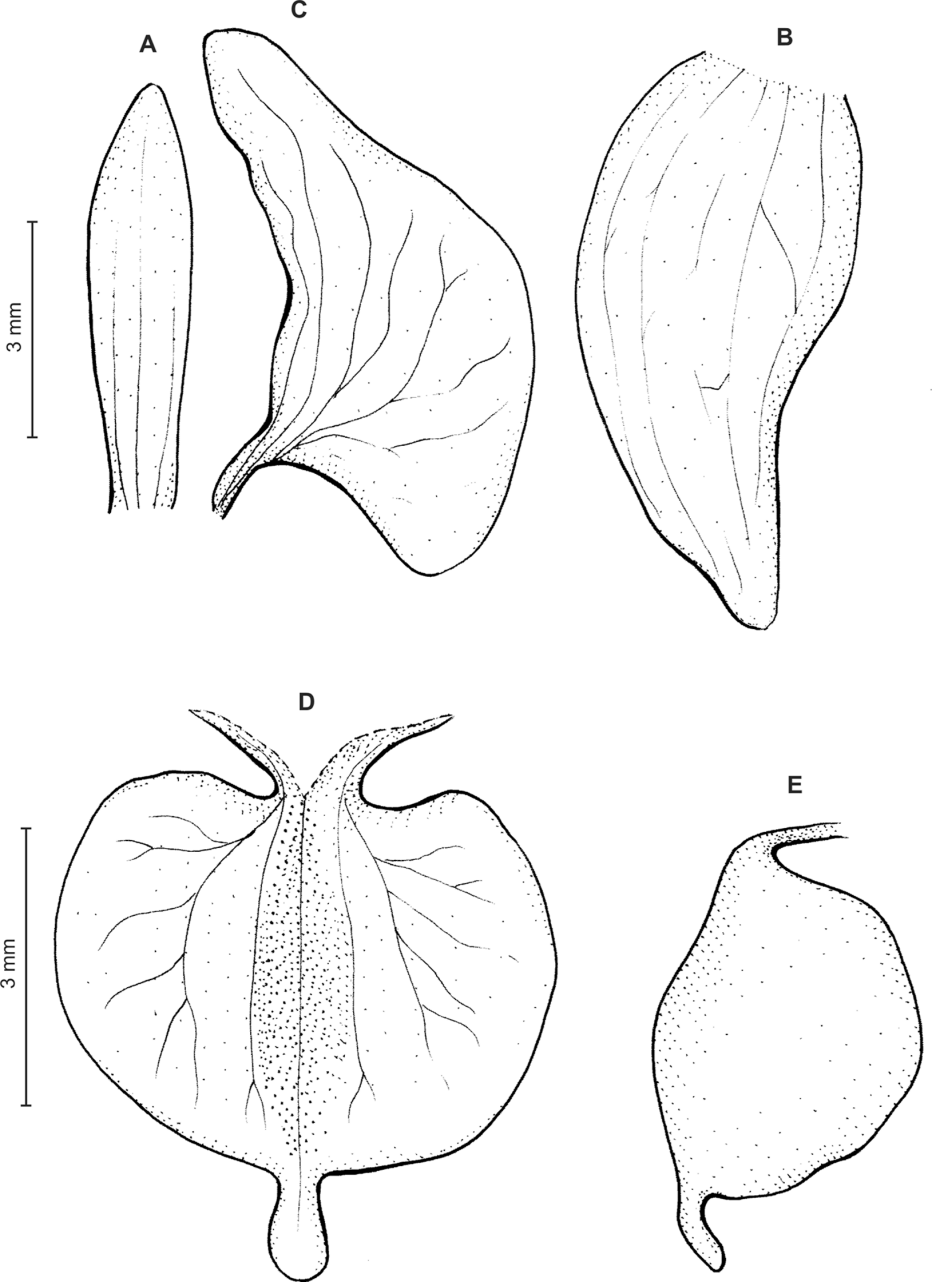
*Ponthieva camargoi* Szlach. & Kolan. (A) Dorsal sepal, (B) lateral sepal, (C) petal, (D) lip, front view, (E) lip, lateral view. Drawn by P. Baranow from *Huertas & L.A. Camargo G. 6971*.

Plants up to 50 cm tall. Leaves 5–7, basal, rosulate, petiolate; petiole up to three cm long, narrow; blade up to 10 cm long and three cm wide, ovate-lanceolate to oblanceolate, acute, glabrous. Peduncle erect, slender to relatively stout, glandular above lower third, otherwise glabrous, with up to six cauline sheaths, terminated by a sublaxly many-flowered raceme up to 17 cm long. Flowers green with cream margins. Floral bracts up to 15 mm long, lanceolate, glandular. Pedicellate ovary up to nine mm long, densely glandular. Sepals almost glabrous. Dorsal sepal six mm long, 1.5 mm wide, ligulate, obtuse, obscurely 3-veined. Petals unguiculate; claw free part 1.5 mm long, narrow; lamina seven mm long, 3.2 mm wide, obliquely elliptic-ovate in the lower part, ligulate above, obtuse at both ends, auriculate basally, 2-veined, veins branching, margins glabrous. Lateral sepals seven mm long, three mm wide, ovate-lanceolate, subobtuse, somewhat falcate, free to the base, 5-veined. Lip unguiculate, claw about 1.5 mm long; lamina 5.2 mm long in total, 5.5 mm wide when spread, obreniform in general outline, subcordate at the base, concave in the center, glabrous, nerves 5, branching, apical projection 1.2 mm long and 0.5 mm wide, linear-ligulate, obtuse. Gynostemium five mm long (Fig. 8).

*Ecology*: Terrestrial in premontane forest. Flowering in April, May, October, November, and December.

*Distribution*: Ecuador, Colombia, Venezuela. Alt. 1,235–2,300 m.

*Representative specimens:* COLOMBIA. **Antioquia**: Cordillera Central, a orillas de la Carretera entre Medellín y Guarne, 1,500–1,900 m, 5 May 1946, *G. Gutierrez V. 1035* (AMES!, COL!)*;* **Cesar**: Mpio. Agustin Codazzi, Serrania de Perijá, vereda Siete de Agosto. Colecciones desde la base de la cuchilla Macho Solo en margen del Rio Guajirita, y ascendiendo hacia la finca cafeteria El Oasis, en sector degradados, sometidos a quemas, 9°57′11.1″-33.2″N, 73°02′46.4″-03′20.9″W, 1,235–1,662 m, 12 Dec. 2005, *O. Rivera Diaz & al. 2936* (COL!); **Cundinamarca**: Mpio. Albán, granjas del Padre Luna. Bosque al borde de el quebrada La Maria, 4°53′N, 74°26′W, 1,800 m, 16 Apr. 2001, *M. Chaparro & al. 27* (COL!); Mpio. Chipaque. Road from Bogotá to Villavicencio, two km below Chipaque, 2,200 m, 31 Oct. 1949, *W. R. Philipson, J.M. Idrobo & A. Fernandez 1289* (COL!); Mpio. Cáqueza. Cáqueza, acueducto de la Planta Electrica, 1,700 m, 26 Nov. 1963, *L. Uribe U. 4597* (COL!); Mpio. Quetame, 1,500 m, 28 Nov. 1963, *L. Uribe U. 4615* (COL!); Mpio. San Antonio del Tequendama. Santandercito, subiendo a Laguna Seca, 1,900 m, 7 Apr. 1962, *L. Uribe U. 4006* (COL!); **Norte de Santander**: Mpio. Toledo. Cordillera Oriental, hoya de Samaria, 2,000–2,100 m, 30 Oct. 1941, *J. Cuatrecasas & al. 12805* (AMES!, COL!, F!, US!, UGDA!—drawing); **Santander**: Mpio. La Paz. Cordillera Oriental, alrededores del Hoyo del Aire, 1,943 m. 7 Nov. 1975, *G. Huertas & L.A. Camargo G. 6971* (COL!). (Fig. 7).

*Other materials examined*: ECUADOR. **Pichincha**: Volcan Pululahua, 35 km NW of Quito, beyond the Monument to the Center of the Earth, 2,300 m, May 1985, *A. Hirtz 2918* (QCNE!, RPSC!, UGDA!—drawing). VENEZUELA. **Táchira**: Distr. Junín. La Pena, along Delicias-Rubio Highway, 1,900 m, 15 Nov. 1982, *G. Davides & A.C. Gonzalez 22277* (COL!).

*Notes*: This species is similar to *P. micromystax* from which it can be distinguished by unguiculate petals and lip. In both cases the claws measure ca 1.5 mm long.

*Ponthieva racemosa* (Walter) C. Mohr, Contr. U. S. Natl. Herb. 6: 460. 1901. *Arethusa racemosa* Walter, Fl. Carol.: 222. 1788; TYPE: USA. *Walter s.n*. (BM-000061837).

**Figure 9:**
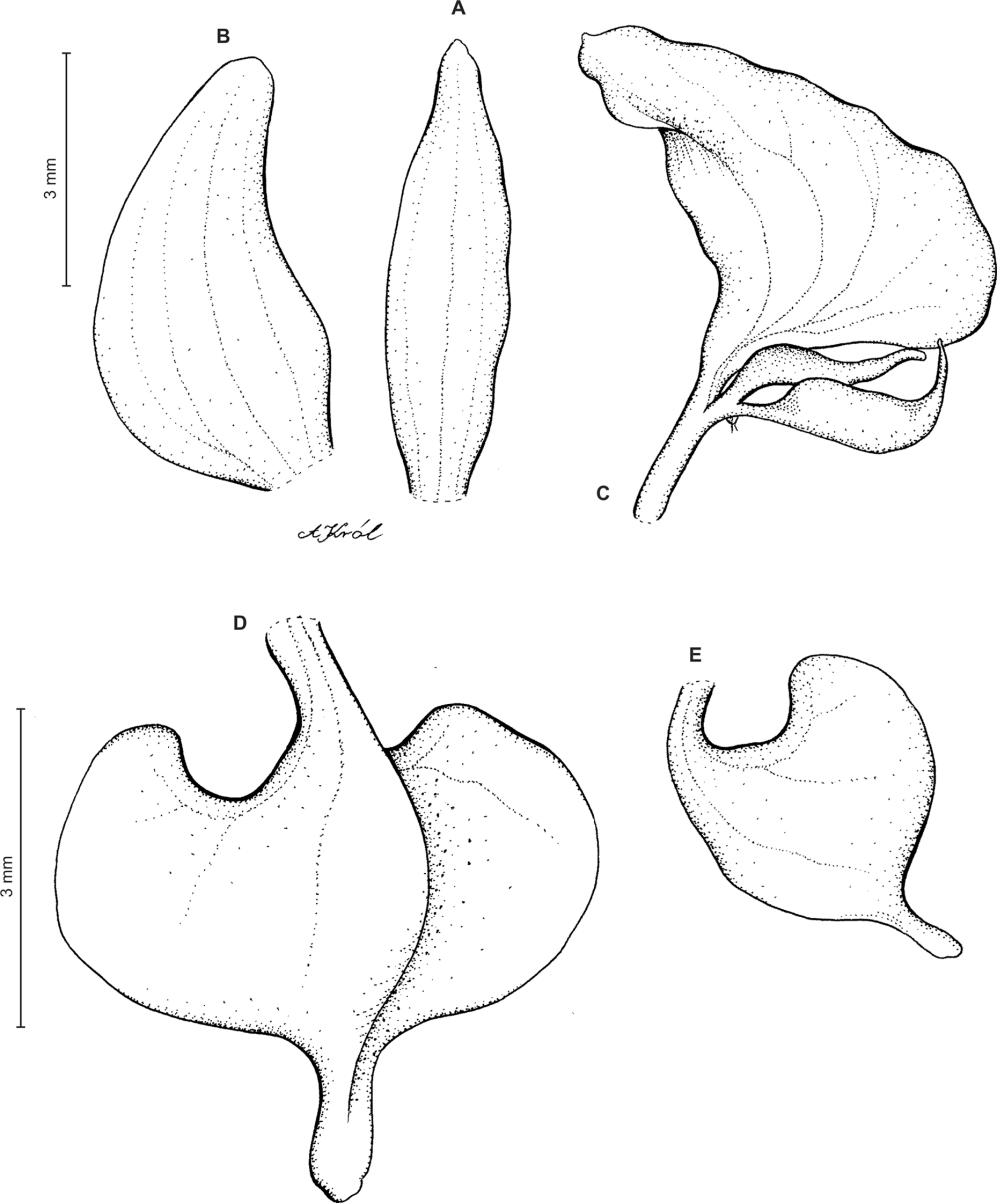
*Ponthieva racemosa* (Walter) Mohr. (A) Dorsal sepal, (B) lateral sepal, (C) petal, gynostemium and lip, (D) lip, front view, (E) lip, side view. Drawn by A. Król from *Poeppig s.n*.

Plants scapose, 13–60 cm tall. Leaves 3–6, mostly in a basal rosette, subsessile to long-petiolate, 2–17 cm long (including the petiole), 1–5.5 cm wide, oblong-elliptic, obovate or oblanceolate, obtuse or subacute, glaucous on the lower surface. Peduncle erect, glandular-pubescent, terminated by a laxly several-flowered raceme 5–24 cm long. Flowers white-green, marked with green. Floral bracts 5–10 mm long, ovate-lanceolate to narrowly lanceolate, acuminate, glandular. Pedicellate ovaries 10–22 mm long, rather stout, glandular. Dorsal sepal 3.8–7.5 mm long, 2–3 mm wide, oblong-elliptic to elliptic-lanceolate, obtuse to subacute, 3- or 5-veined. Petals subsessile, four to eight mm long, 1.5–5 mm wide at the base, obliquely triangular to semicordate, incurved, dilated on the outer margin at the base, somewhat constricted near the apex, obtuse to subacute, mostly ciliate along outer margin, 3-veined, veins branching. Lateral sepals 4.3–8 mm long, 2.5–4 mm wide, free to the base, broadly ovate to ovate-oblong, oblique, somewhat falcate at the apex, obtuse to acute, 5-veined. Lip shortly unguiculate, claw one to two mm long; lamina 4–7.5 mm long, 2.5–6 mm wide when spread out, suborbicular to transversely elliptic in general outline, concave with the lateral margins upturned, truncate or subcordate at base, rounded at apex, terminated by a linear, obtuse to acute apical lobe; disc papillose, with an obscure linear median crest. Gynostemium 4.5 mm long in total, shortly stalked (Fig. 9).

*Ecology*: Terrestrial in deep shade in wet tropical forest. Flowering in January, March, May, November.

*Distribution*: USA, Mexico, Guatemala, El Salvador, Honduras, Nicaragua, Costa Rica, Cuba, Panama, Bolivia, Peru, probably Ecuador, Colombia, Venezuela. Alt. 600–1,400 m.

*Representative specimens:* COLOMBIA. **Cundinamarca**: Mpio. Albán. Finca Los Sauces, Vereda Jaba, carratera Bogotá-Guaduas, 48 km W de Bogotá, Cordillera Oriental, vertiente occidental, bosque nativo secundario, 22 May 1999, *F. Silverstone-Sopkin & R. Clavijo 8247* (CUVC!); **Magdalena**: Santa Marta, 600 m, *H. Smith 2553b* (AMES!); **Valle del Cauca**: Mpio. Buga. Cordillera Central, vertiente occidental, carratera Buga-Nogales, 10.9 km arriba de iglesia del pueblo de La Habana, Hacienda La Aurora, 14 Nov. 1981, *F. Silverstone-Sopkin 931* (CUVC!, MO!); Cordillera Central, La Marina, 1,400 m, Mar. 1942, *E. Dryander 2493* (US!—in buds) (Fig. 7).

*Other materials examined:* VENEZUELA. Mérida, 1842, *J.W.K. Moritz 16(1104)* (W-R!, UGDA!—drawing); *Sine loc., J.W.K. Moritz s.n*. (AMES!). CUBA. Cahobas. In sylvis obscuris, Jan. 1824, *E. Poeppig s.n*. (W!, UGDA!—drawing).

*Notes*: This species is similar to *P. rostrata*, but unlike the latter its lip is unguiculate. The claw is narrow, canaliculated, with thin margins, what separates it from *P. castanedae*. The truncate petals base distinguishes *P. racemosa* from both *P. micromystax* and *P. camargoi*.

*Ponthieva castanedae* Szlach. & Kolan., Pl. Syst. Evol. 299(9): 1673. 2013. TYPE: Colombia. *J. Cuatrecasas & R. Romero-Castaneda 24716* (holotype, COL-84360!; isotype, US-00292540!; UGDA!—drawing).

**Figure 10:**
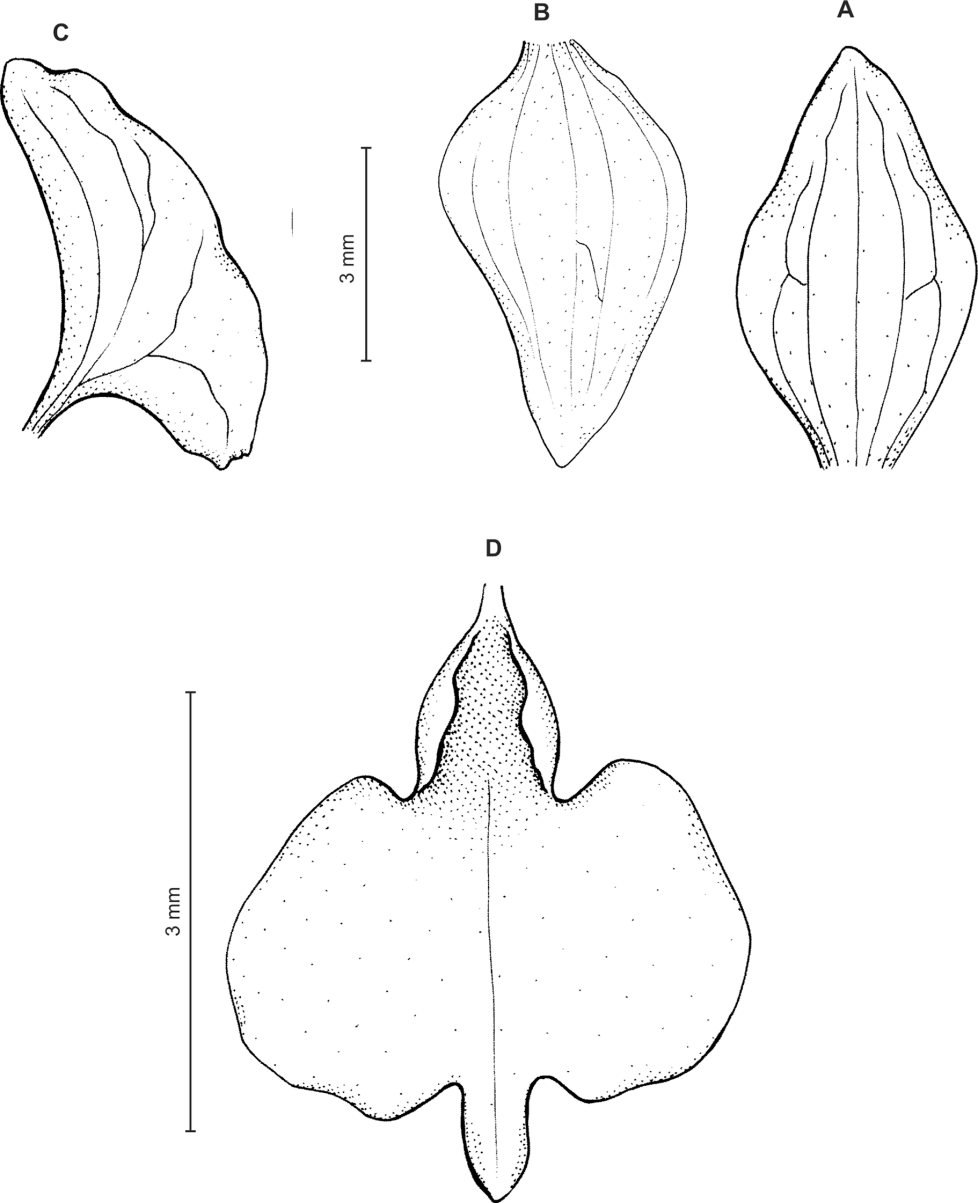
*Ponthieva castanedae* Szlach. & Kolan. (A) Dorsal sepal, (B) lateral sepal, (C) petal, (D) lip, front view. Drawn by P. Baranow from *Cuatrecasas & R. Romero-Castaneda 24716*.

Plants 14–28 cm tall. Leaves 2–3, basal, rosulate, petiolate; petiole up to 3.5 cm long; blade up to five cm long and 2.3 cm wide, ovate, oblong-lanceolate to oblong-ovate, acute, glabrous. Peduncle delicate, erect, glabrous, glandular, with three cauline sheaths, terminated by a loosely few-flowered raceme usually up to three cm long. Flowers small, white, sepals sparsely glandular. Floral bracts six mm long, lanceolate, glabrous. Pedicellate ovary up to 11 mm long, densely glandular. Dorsal sepal six mm long, three mm wide, ovate-deltoid, widest near the middle, subobtuse, with 5, sparsely anastomozing veins. Petals unguiculate; claw free part 1–1.2 mm long, narrow; lamina 4.6 mm long, 2.3 mm wide, obliquely oblong-ovate, obtuse at both ends, with three branching veins, margins glabrous. Lateral sepals six mm long, 3.8 mm wide, obliquely ovate, subobtuse, somewhat falcate, free to the base, 5-veined. Lip unguiculate, claw free part 1.5 mm long, broadly ovate above narrow base, margins upcurved, foming a kind of channel; lamina 2.5 mm long, five mm wide, transversely elliptic in general outline, auriculate or cordate at the base, subcordate at the apex, concave, glabrous, 3-lobed apically; lateral lobes obliquely elliptic-ovate; middle lobe 0.5 mm long, ca 0.3 mm wide, linear, obtuse, appearing at the bottom of shallow sinus. Gynostemium 2.6 mm long in total, shortly stalked (Fig. 10).

*Ecology*: Terrestrial in the fields and forests. Flowering in October.

*Distribution*: Colombia. Alt. 2,400–2,600 m.

*Representative specimen:* COLOMBIA. **Magdalena**: Sierra Nevada de Santa Marta. SE slopes, Hoya del Rio Donachui, Cancurua, fields and forests, 2,400–2,650 m, 10–11 Oct. 1959, *J. Cuatrecasas & R. Romero-Castaneda 24716* (COL!, US!) (Fig. 7).

*Notes*: This species has been originally compared with Ecuadorian-Peruvian congener—*P. parvilabris* (Lindl.) Rchb.f. (Szlachetko & Kolanowska, 2013). Both species differ in floral segments. Although, similar in general outline, the lip makes them easily separable. The lip of *P. castanedae* is prominently auriculated basally, and devoided of any calli, the claw margins are upcurved forming a kind of channel directed to the base of the lip. In *P. parvilabris* lip claw is semiterete, lamina possesses cuneate base, and a pair of keel-like, fleshy calli at base. Additionally, petal’s claw of this species is shorter than lower lobe, whereas in *P. parvilabris* petal’s claw is as long as or longer than lower lobe. Lateral sepals of *P. castanedae* are obliquely ovate versus obliquely elliptic-ovate in *P. parvilabris. Ponthieva castanedae* can be distinguished from other representatives of *P. rostrata* group by having relatively wide claw with upcurved margins. Claw lateral lobes are oblong elliptic and the base of lamina is cordate.

*Ponthieva pubescens* (C. Presl) C. Schweinf., Fieldiana, Bot. 33: 5. 1970. *Ophrys pubescens* C. Presl, Rel. Haenk. 1: 91. 1827. TYPE: Peru. *T. Haenke s.n*. (lectotype, designated by Garay (1978: 224), PR).
*Ponthieva montana* Lindl., Pl. Hartw.: 155. 1845. TYPE: Ecuador. Loja. *K.T. Hartweg 834* (lectotype, designated by Garay (1978: 224), K-L!; isolectotypes, E!, FI-011848!, P-00363558!).

**Figure 11:**
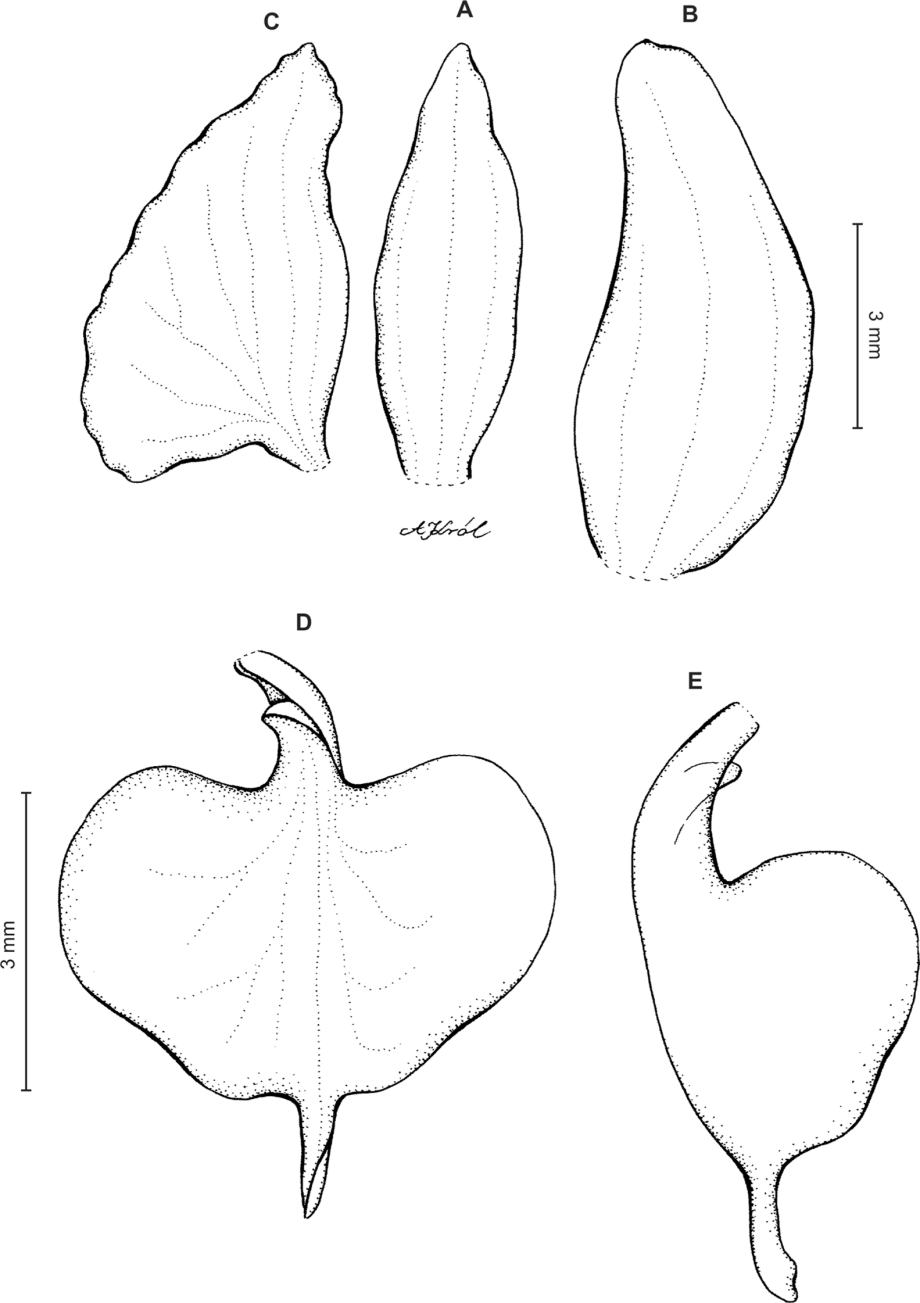
*Ponthieva pubescens* (C.Presl) C. Schweinf. (A) Dorsal sepal, (B) lateral sepal, (C) petal, (D) lip, front view, (E) lip, side view. Drawn by A. Król from *Poeppig s.n*.

Plants up to 32 cm tall. Leaves 3–8, basal, rosulate, petiolate; petiole up to two cm long; blade up to nine cm long and 2.6 cm wide, elliptic to oblong-elliptic, occasionally ovate, acute to obtuse, glabrous. Peduncle erect, slender, glabrous below, glandular-pubescent above, with three to four distant sheaths, terminated by a loosely 5–15-flowered raceme. Flowers small, white, often spotted or veined with green or brown, sepals sparsely glandular-pubescent on the outside. Floral bracts up to nine mm long, ovate-lanceolate, acute to subacuminate. Pedicellate ovary up to 13 mm long, densely glandular. Dorsal sepal up to five mm long and two mm wide, narrowly elliptic to lanceolate-elliptic, acute, 3-veined. Petals shortly unguiculate to subsessile, up to five mm long and two mm wide, obliquely elliptic-ovate with obtuse to rounded lobes, glabrous, veins 2, more or less branching. Lateral sepals up to six mm long and 3.2 mm wide, free to the base, obliquely ovate, acute to obtuse, 3-veined. Lip unguiculate, claw ca 1.2–1.5 mm long, with 2 small, long decurrent keels; lamina up to three mm long and five mm wide, often less, transversely elliptic, with truncate-rounded base and apex, concave to conduplicate, gibbose at base, disc with 3–5-veins more or less branching lateral veins, glabrous, 3-lobed; the middle lobe short, linear-oblong, obtuse; lateral lobes rounded. Gynostemium ca four mm long in total, shortly stalked (Fig. 11).

*Ecology*: No information about habit. Flowering in May and October, in January in Cuba.

*Distribution*: Peru, Ecuador, Colombia, Cuba. Alt. 2,400 m.

*Representative specimen:* COLOMBIA. **Valle del Cauca**: La Paila, 9 May 1853, *I. Holton 202* (AMES!, UGDA!—drawing) (Fig. 7).

*Other materials examined*: PERU. Prope Tarapoto, 1855–1856*, R. Spruce 4578* (AMES!, P!, W!); *Sine loc. K.T. Hartweg 834* (P!, W!). ECUADOR. **Loja**: Loja, *K.T. Hartweg s.n*. (K-L!); **Tungurahua**: Tungurahua, 2,400 m, 31 Oct. 1879, *F.C. Lehmann 321* (W!), Tungurahua, 1857–1859, *R. Spruce 5218* (P!, W!). CUBA. Cahoba, Jan. 1824, *E. Poeppig s.n*. (W-R).

*Notes*: This species is characterized by the basal lip callus consisting of two keels decurrent gradually on prominent claw, a character not found in any representatives of *P. rostrata* group.

**2. *Venusta* group**

Plants with usually basal, sessile to petiolate, glabrous leaves, with small flowers which lip is obcordate to triangular in general outline, cuneate basally and truncate to emarginated apically, with elongate apical lobe and shortly stalked gynostemium.

## Key to the Species

1. Margins of petals glabrous*P. gracilis*
1* Margins of petals ciliolate or glandular2
2. Lip lamina ecallose3
2* Lip lamina with prominent calli4
3. Lateral sepals free to the base*P. vallecaucana*
3* Lateral sepals more or less connate*P. parvilabris*
4. Petals occasionally with a few, bristle-like papillae at the obtuse angles*P. venusta*
4* Petals glandular5
5. Lip lamina with somewhat thickened two plates on both sides of midvein, petals obliquely triangular*P. lilacina*
5* Lip without any thickened plates, petals obliquely ligulate-lanceolate to ligulate-ovate, subfalcate*P. microglossa*

*Ponthieva gracilis* Renz, Candollea 11: 266, Fig. 9B. 1948. TYPE: Colombia*. O. Renz 4153* (holotype, RENZ-4153.1!; isotypes, RENZ-4153.2!, RENZ-4153.3!).

**Figure 12:**
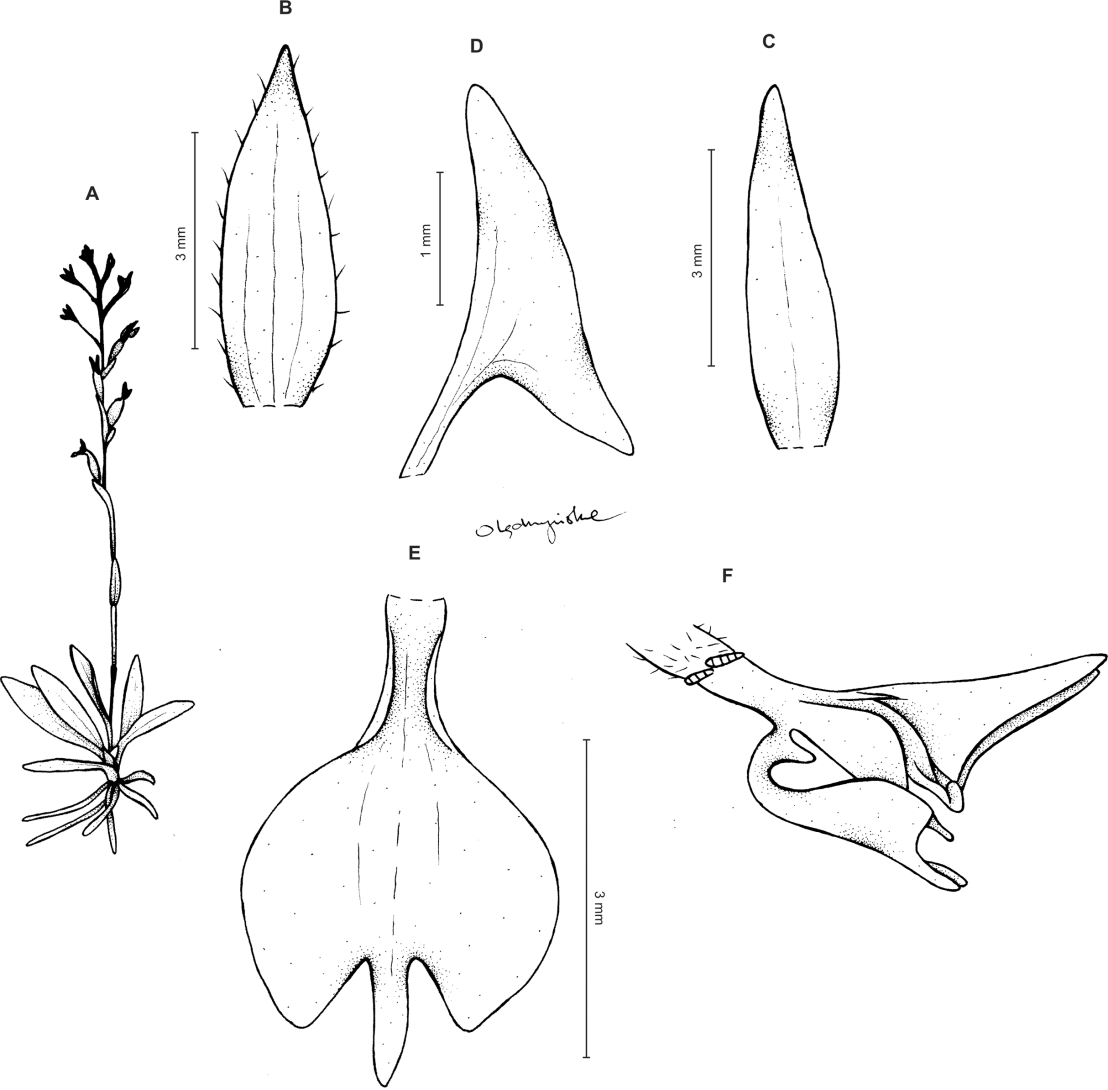
*Ponthieva gracilis* Renz. (A) Habit, (B) dorsal sepal, (C) lateral sepal, (D) petal, (E) lip, front view, (F) lip, petal and gynostemium. Drawn by A. Król from the holotype.

Plants ca 10–25 cm tall. Leaves 5–9, basal, rosulate, shortly petiolate; blade two to three cm long, 0.5 cm wide, oblong lanceolate to linear-oblanceolate, acute to subobtuse, glabrous. Peduncle erect, delicate, glabrous below, pubescent above, 3-sheathed, terminated by a laxly many-flowered raceme about three to eight cm long. Flowers small, sepals pubescent-glandular on the outer surface. Floral bracts ca five mm long, lanceolate, pubescent-glandular. Pedicellate ovary up to nine mm long, densely pubescent-glandular. Dorsal sepal up to five mm long, two mm wide, oblong-lanceolate, subobtuse, 3-veined. Petals 3–3.5 mm long, about two mm wide, prominently unguiculate; claw linear, narrow; lamina narrowly falcate-triangular in outline, with acute both ends, the lower one with prominently elongate outer margin, veins 2, sparsely branching, margins glabrous. Lateral sepals up to six mm long, two mm wide, free to the base, obliquely lanceolate-ovate, subobtuse, 3-veined. Lip three mm long, up to three mm wide, unguiculate; claw U-shaped, with single, transverse callus at the base; lamina obcordate in general outline, widest near the middle, apically 3-lobed; middle lobe linear-lanceolate, subacute, exceeding both laterals; lateral lobes semi-elliptic-obovate, rounded. Gynostemium shortly stalked, erect (Fig. 12).

*Ecology*: Terrestrial. Flowering in December and January.

*Distribution*: Colombia. Alt. 600 m.

**Figure 13:**
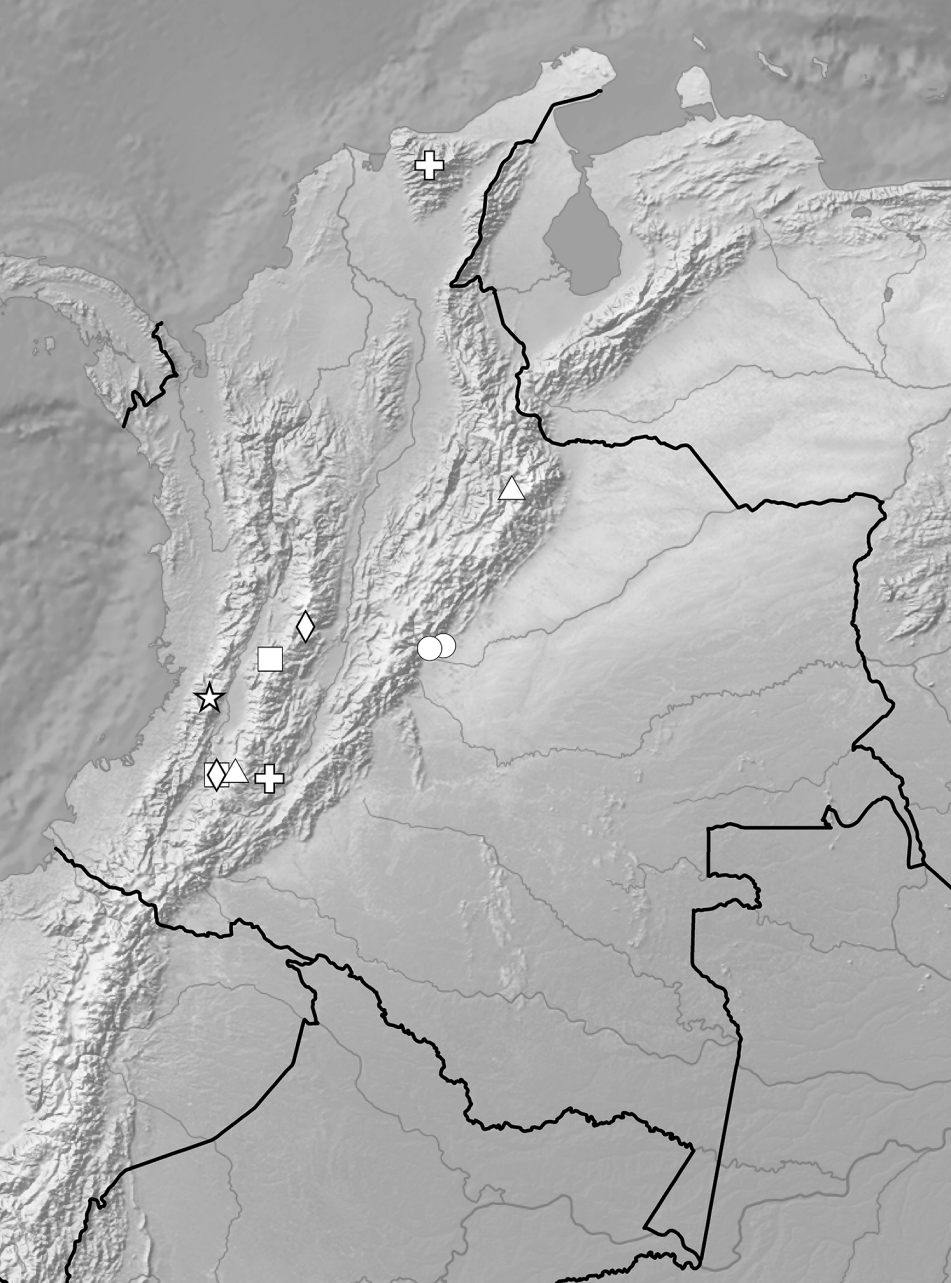
Distribution of representatives of *Venusta*-group. *P. gracilis* (circle), *P. lilacina* (square), *P. microglossa* (triangle), *P. parvilabris* (diamond), *P. vallecaucana* (star), *P. venusta* (cross). Map generated in ArcGis 9.3 (Esri, Redlands, CA, USA).

*Notes*: This species shares with other representatives of *P. venusta* group the similar lip shape, which in general outline is obcordate with much elongate apical middle lobe. The character which separates it from other species is the form of petal, which is narrowly falcate-triangular in outline, with acute both ends and prominently elongate outer margin.

*Representative specimens:* COLOMBIA. **Meta**: Villavicencio, Río Guacavía, 600 m, 1 Jan. 1938, *O. Renz 4097* (RENZ—Dueñas Gómez & Fernández Alonso, 2009), Río Negro unterhalb der Hauser Servitá, 600 m, 1 Dec. 1939, *O. Renz 4153* (RENZ—Dueñas Gómez & Fernández Alonso, 2009) (Fig. 13).

*Ponthieva venusta* Schltr., Repert. Sp. Nov. Regni Veg., Beih. 9: 57. 1921. TYPE: Peru. *A. Weberbauer 529* (B†; lectotype, designated by Garay (1978: 228), AMES-00103574!—drawing).

**Figure 14:**
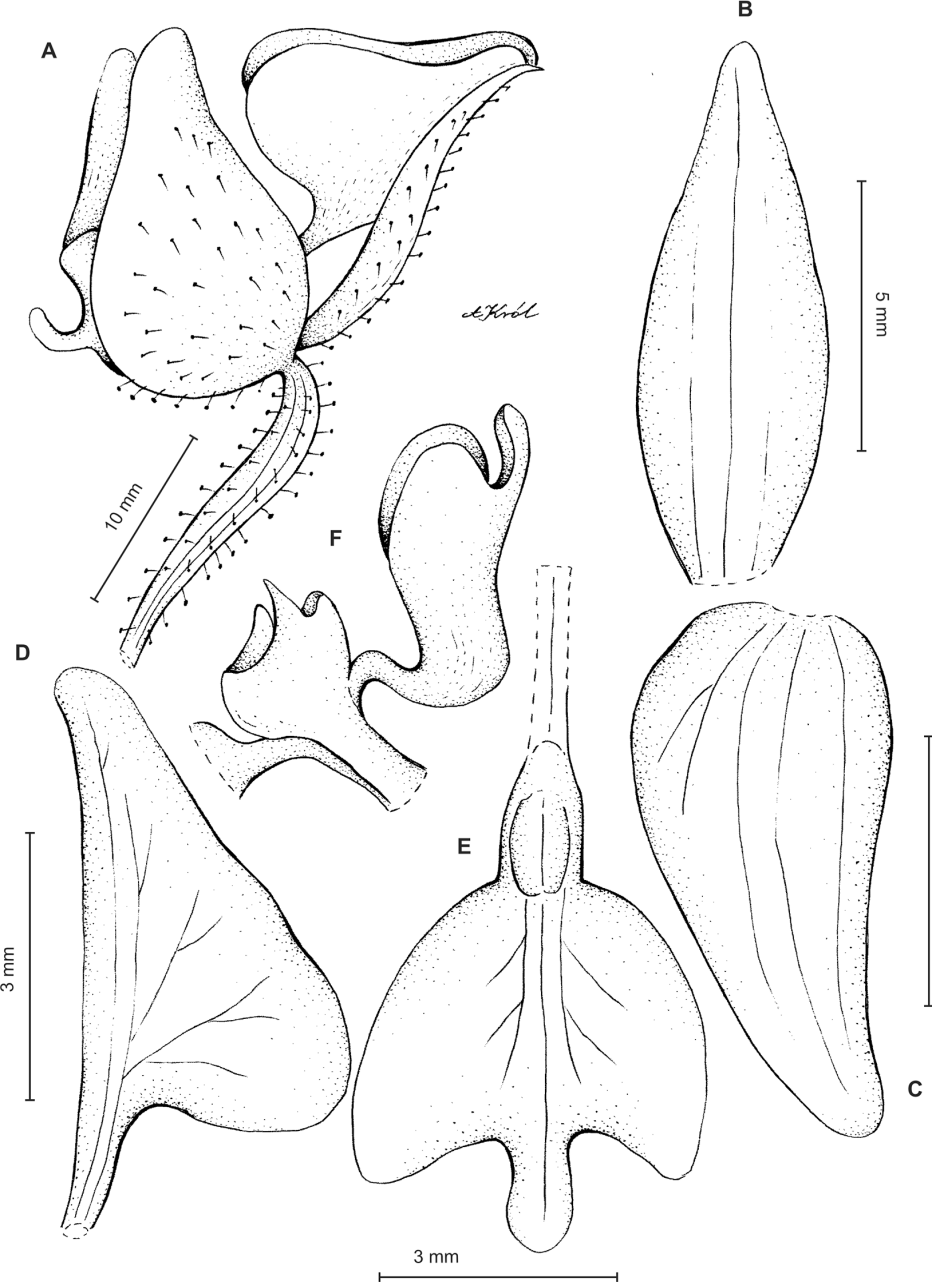
*Ponthieva venusta* Schltr.—original Schlechter’s illustration reproduced by Mansfeld (1929) and redrawn by A. Król. (A) Flower, (B) dorsal sepal, (C) lateral sepal, (D) petal, (E) lip, front view, (F) gynostemium and lip. Scale bars made based on measurements provided by Schlechter in species description.

Plants up to 40 cm tall, erect or ascending. Leaves 3–5, basal, petiolate; petiole 3–4.5 cm long; blade five to nine cm long, 2.5–4 cm wide, elliptic to obovate-elliptic, acute, glabrous. Peduncle erect, apically glandular-puberulent, remotely 3-sheathed, terminated by a loosely several- to many-flowered raceme up to 10 cm long. Flowers white, sepals sparsely pubescent-glandular externally. Floral bracts up to 10 mm long, ovate-cucullate, acuminate. Pedicellate ovary up to 15 mm long. Dorsal sepal up to 10 mm long and three mm wide, elliptic to ovate-elliptic, acute, 3-veined. Petals unguiculate; claw free part up to two mm long; lamina up to seven mm long and three mm wide, obliquely triangular-dolabriform, basally subauriculate, occasionally with a few, bristle-like papillae at the obtuse angles, veins 2, the outer one branching. Lateral sepals up to 10 mm long and five mm wide, free to the base, obliquely ovate, acute, 4-veined. Lip up to four mm long and 2.8 mm wide when spread, distinctly unguiculate; claw narrow; lamina 3-lobed, obcordate in general outline, disc at base with a pair of approximate calli; the middle lobe ligulate, obuse; lateral lobes obliquely triangular-obovate with obtuse apex. Gynostemium ca three mm long, rather massive, shortly stalked (Fig. 14).

*Ecology*: Terrestrial. Flowering in July and October.

*Distribution*: Peru, Ecuador, Colombia. Alt. 1,200–2,000 m. This species was reported from Ecuador by Garay (1978: 228).

*Representative specimens:* COLOMBIA. **Huila**: Mpio. de La Plata, Vereda Agua Bonita. Finca Merenberg, 1,200–1,300 m, 14 Jul. 1975, *S. Diaz P. & al. 527* (COL!); **Magdalena**: Sierra Nevada de Santa Marta. Valle del Rio Donachuy, camino Sacaracungua-Canguruaca, 2,000 m, 14 Oct. 1958, *T. van der Hammen 1110* (COL!) (Fig. 13).

*Other material examined*: PERU. **Cuzco**: Sandia, *A. Weberbauer 529* (AMES!—drawing).

*Notes*: In the time of description Schlechter (1921) classified this species in *P. montana* group and stated i.a. that it has much larger flowers and differently formed lip lamina. It is characterized by obcordate lip with a pair of approximate calli at the base of lamina.

*Ponthieva microglossa* Schltr., Repert. Spec. Nov. Regni Veg., Beih. 7: 64. 1920 *non* Schltr. 1921 (= *P. inaudita*). TYPE: Colombia, *M. Madero s.n*. (B†; lectotype, designated by Garay (1978: 221), AMES-00103543!—drawing).

**Figure 15:**
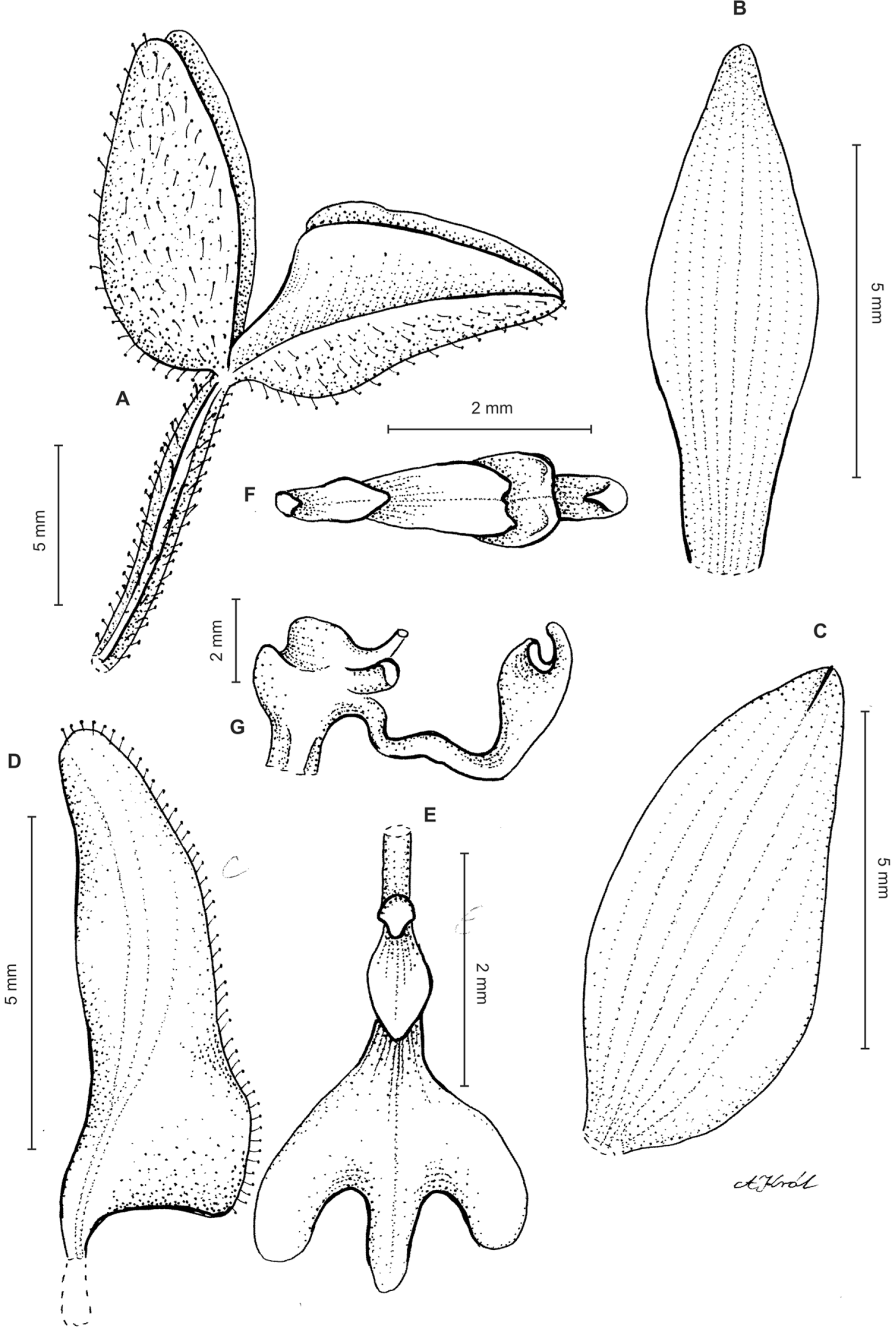
*Ponthieva microglossa* Schltr.—original Schlechter’s illustration reproduced by Mansfeld (1929) and redrawn by A. Król. (A) Flower, (B) dorsal sepal, (C) lateral sepal, (D) petal, (E and F) lip, (G) gynostemium and lip. Scale bars made based on measurements provided by Schlechter in species description.

Plants 18–27 cm tall, erect. Leaves 2–3, basal, petiolate; petiole three to five cm long, canaliculate; blade 5.5–7 cm long, 2–3.5 cm wide, elliptic, acuminate, cuneate at the base, glabrous. Peduncle subflexuose, densely puberulent, 2–3-sheathed, terminated by a laxly 4–6-flowered raceme three to five cm long. Flowers medium-sized to rather small, sepals glandular-puberulent. Floral bracts five mm long, elliptic-lanceolate, acuminate, sparsely glandular along margins and at base. Pedicellate ovary 10 mm long, densely glandular-puberulent. Dorsal sepal 7.5–8 mm long, 2.4–3.2 mm wide, broadly lanceolate to lanceolate-ovate, subobtuse, veins 5, branching. Petals unguiculate, claw free part ca one mm long; lamina six to eight mm long, 2.2–2.4 mm wide, obliquely ligulate-lanceolate to ligulate-ovate, subfalcate, obtuse, truncate at the base, glandular on outer margin, obscurely 1–2-veined. Lateral sepals eight to nine mm long, three to four mm wide, free to the base, obliquely elliptic to elliptic-ovate, slightly concave at the base, subobtuse, 5-veined, veins branching. Lip unguiculate, 3.5–3.8 mm long in total, two mm wide when spread, lamina obtriangular from cuneate base, basal callus bilobed, both lobes upcurved and directed to each other, apex obcordate, 3-lobed; the middle lobe ligulate, obtuse, incurved; lateral lobes falcately-elliptic, obtuse, upcurved. Gynostemium 2.5 mm long, massive, shortly stalked (Fig. 15).

*Ecology*: Terrestrial in the premontane low forest and high-montane forest. Flowering in April and October.

*Distribution*: Colombia. Alt. 1,800–2,750 m.

*Representative specimens:* COLOMBIA. **Boyacá**: Mpio. San Mateo, Guicas Buraga, Cañon de Chicamocha, 1,800 m, 3 Oct. 1991, *A. Etter & al. 903(699, 268)* (COL!); **Cauca**: *Sine loc.*, 2,700 m, *M. Madero s.n*. (B†); *Sine loc.*, 1909–1911, *M. Madero 171* (AMES!—drawing); Guanacas, Popayán, *H. Karsten s.n*. (AMES!) (Fig. 13).

*Notes*: This species is easily distinguishable from other members of the genus by its lip form which is obcordate, 3-lobed, with all lobes being similar in size and form, and basally truncate petals.

*Ponthieva lilacina* C. Schweinf., Bot. Mus. Leafl. Harvard Univ. 9: 224. 1941. TYPE: Peru, *A. Weberbauer 7842* (holotype, AMES-00103537!; isotype, W-9480!, F-0041596F, GH-00103538, UGDA!—drawing).

**Figure 16:**
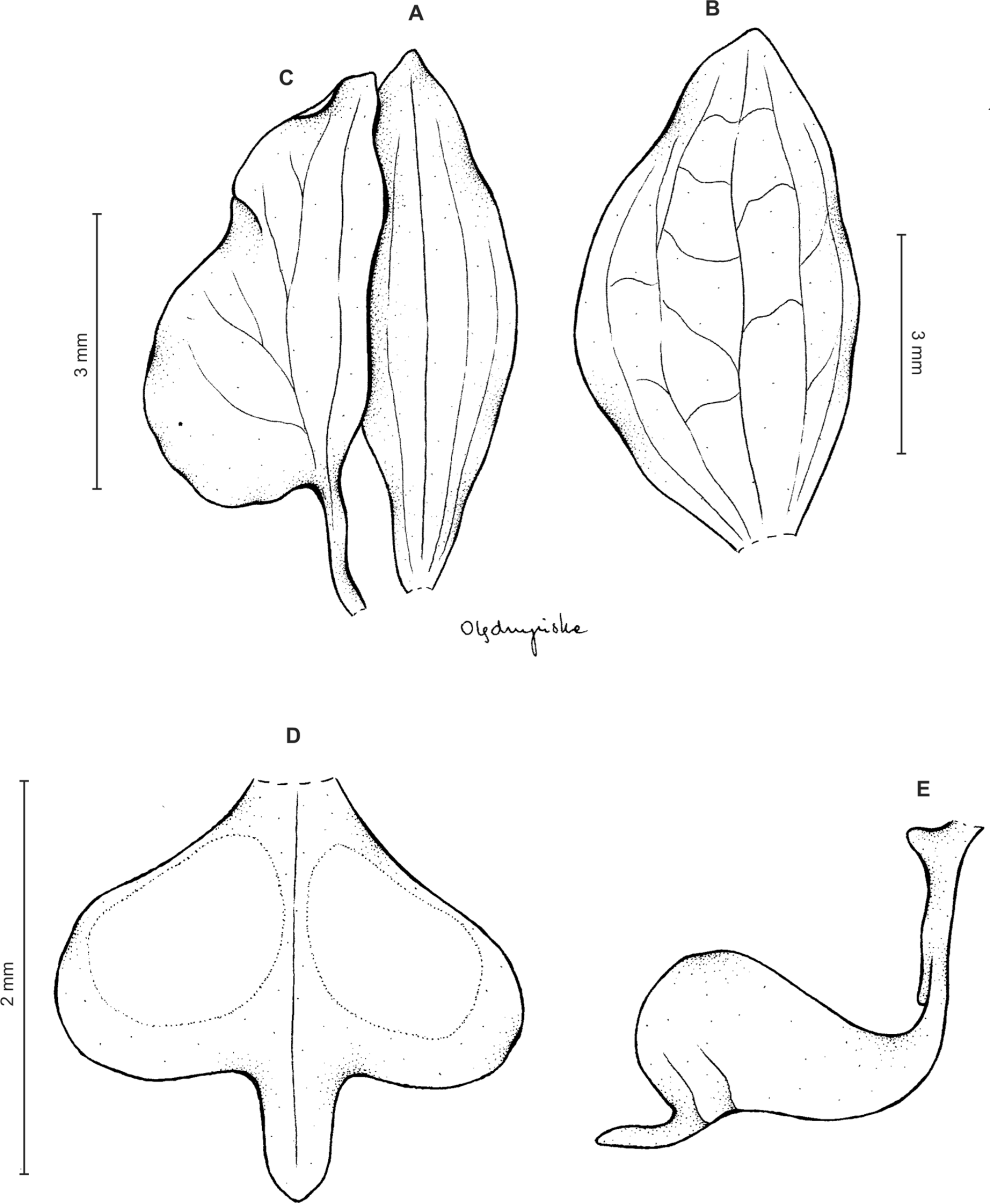
*Ponthieva lilacina* C. Schweinf. (A) Dorsal sepal, (B) lateral sepal, (C) petal, (D) lip, front view, (E) lip, side view. Drawn by N. Olędrzyńska from *Lehmann 7131*.

**Figure 17:**
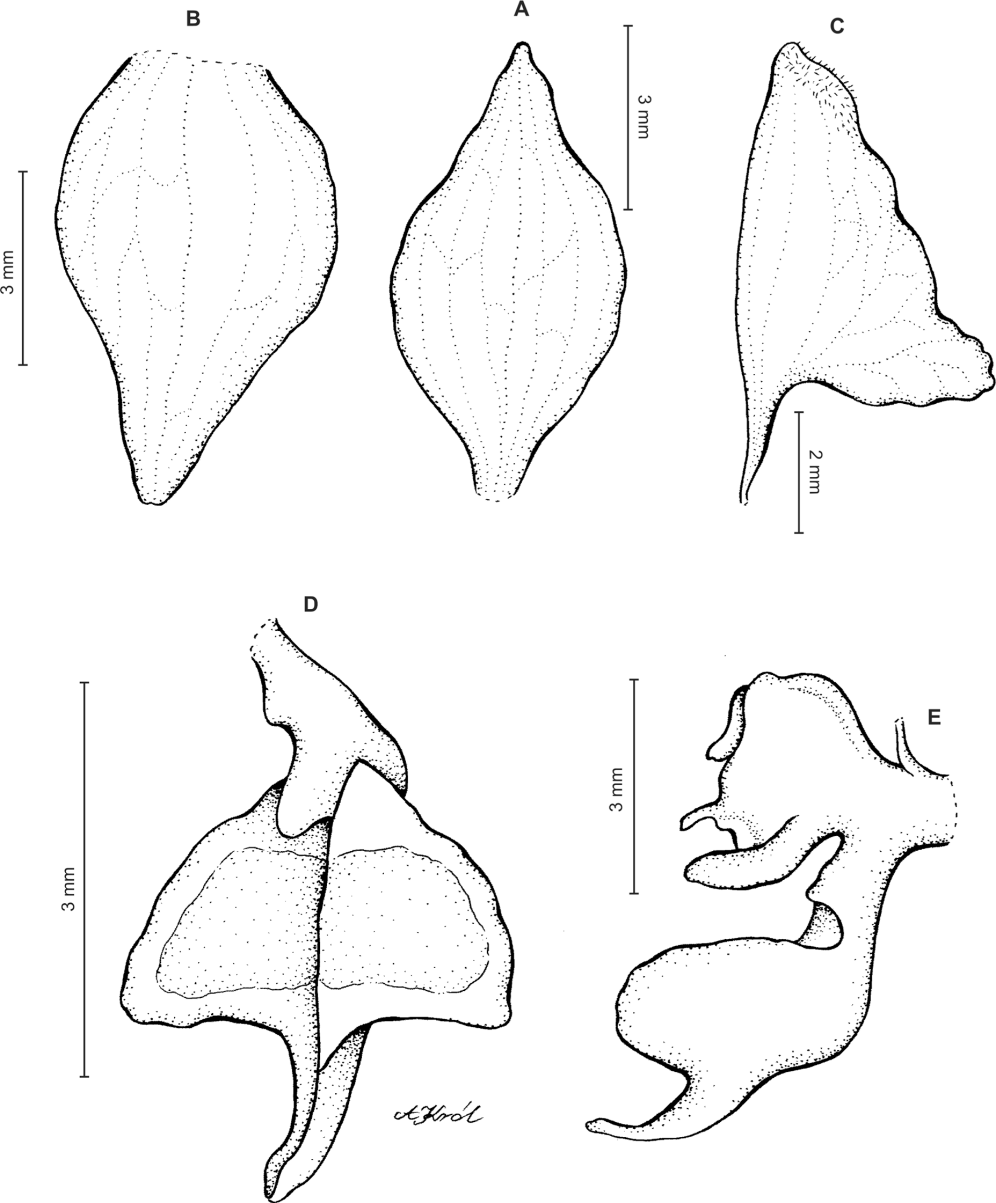
*Ponthieva lilacina* C. Schweinf. (A) Dorsal sepal, (B) lateral sepal, (C) petal, (D) lip, front view, (E) gynostemium and lip, side view. Drawn by A. Król from *Weberbauer 7842*.

Plants up to 23 cm tall, erect. Leaves 1–4, basal, petiolate; petiole up to 5.5 cm long, canaliculate; blade up to 12 cm long and four cm wide, oblong elliptic to broadly oblanceolate, acute to acuminate, cuneate at the base, glabrous. Peduncle finely glandular-pubescent, 2–3-sheathed, terminated by a laxly few- to several-flowered raceme up to nine cm long. Flowers rather small, lilac, sepals glandular-pubescent on the outer surface. Floral bracts five to nine mm long, lanceolate, acuminate, glandular. Pedicellate ovary up to 12 mm long, densely glandular-pubescent. Dorsal sepal 6–7.5 mm long, 2–3.1 mm wide, elliptic to elliptic-ovate, acute, 5-veined, veins branching or simple. Petals unguiculate, claw free part ca two mm long; blade six to seven mm long, 3–3.5 mm wide, obliquely triangular, subacute to obtuse, truncate at the base, 1–2-veined, veins branching, glandular on outer margin. Lateral sepals seven to eight mm long, 4–4.5 mm wide, free to the base, obliquely broadly elliptic to elliptic-ovate, subobtuse, 5-veined, veins branching. Lip unguiculate, claw ca two mm long, with a pair of erect, triangular calli; lamina two to three mm long, ca three to six mm wide, broadly triangular in general outline, with two somewhat thickened plates on both sides of midvein, cordate at the base, truncate at the apex, sharply 3-lobed in front; the middle lobe 0.6–1.5 mm long, ligulate, obtuse, canaliculate; lateral lobes semiobovate. Gynostemium 2.5–3 mm long, massive, shortly stalked (Figs. 16 and 17).

*Ecology*: Terrestrial in evergreen hard-leaved bushwood consisting of trees and shrubs in the shadow. Flowering in April.

*Distribution*: Peru, Colombia. Alt. 1,700–2,750 m.

*Representative specimens*: COLOMBIA. **Cauca**: Rio Cauca above Popayán, 2,200–2,400 m, *F.C. Lehmann 7131* (AMES!, UGDA!—drawing); **Valle del Cauca**: Cordillera Central, vertiente occidental, hoya del rio Bugalagrande, loma de Barragan, desde La Parrilla a La Machuca, 2,660–2,750 m, 13-14 Apr. 1946, *J. Cuatrecasas 20698* (AMES!, F!, US!, UGDA!—drawing) (Fig. 13).

*Other materials examined*: PERU. **Cuzco**: Quispicanchis. Marcapata Valley, in evergreen hard-leaved bushwood consisting of trees and shrubs in the shadow, 1,700–1,800 m, *A. Weberbauer 7842* (AMES!, W!, UGDA!—drawing); **Amazonas**: Chachapoyas, cerros Calla Calla, east side, 10 km above Leimebamba, *P. C. Hutchison & D. E. Bennett 4749* (E!, UGDA!—drawing).

*Notes*: This species is rather similar to *P. microglossa*, but it differs from the latter by the form of petals and lip. Petals have truncate base and the lip is broadly obtriangular with two thickened spots. Both Colombian collections cited above differ from the type specimen by having rounded basal margin of petals. Otherwise, they are similar.

*Ponthieva parvilabris* (Lindl.) Rchb.f., Xenia Orchid. 3: 18. 1878. *Cranichis parvilabris* Lindl., Orchid. Linden.: 27. 1846. TYPE: Venezuela, *J.J. Linden 1470* (lectotype, designated by Garay (1978: 221), K!; isolectotypes, P-00585472!, G, MO-1136291!—photo, US!—photo).
*Ponthieva disema* Schltr., Repert. Spec. Nov. Regni Veg. 14: 116. 1915. TYPE: Ecuador, *P. Mille 26* (B†; lectotype, designated by Garay (1978: 221), AMES-00103515!—drawing; isolectotype, QPLS).

**Figure 18:**
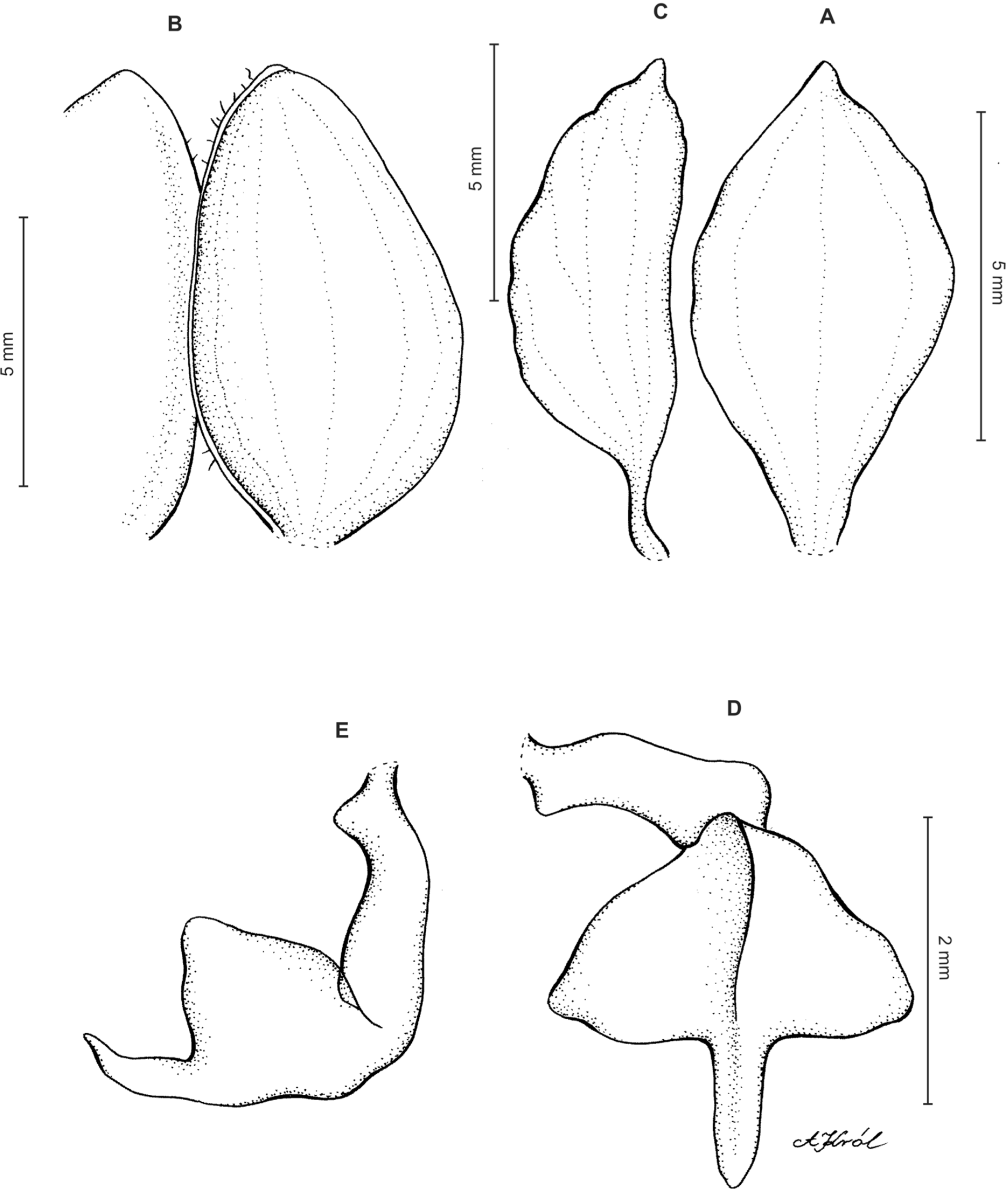
*Ponthieva parvilabris* (Lindl.) Rchb.f. (A) Dorsal sepal, (B) lateral sepals, (C) petal, (D) lip, front view, (E) lip, side view. Drawn by A. Król from *Karsten s.n*.

Plants up to 35 cm tall. Leaves up to 5, basal, rosulate, petiolate; petiole ca 0.5–1(3) cm long; blade up to 6.5 cm long and four cm wide, ovate to ovate-elliptic, acute above, commonly smaller, glabrous. Peduncle erect, rather slender, pubescent above, loosely 3–5 sheathed, terminated by a laxly several-flowered raceme. Flowers white, occasionally with greenish suffusion, sepals pubescent-glandular outside. Floral bracts up to nine mm long, ovate-lanceolate, acute, glandular-pubescent in the lower part. Pedicellate ovary up to 14 mm long, pubescent-glandular. Dorsal sepal 4.3–8 mm long, 2.1–5 mm wide, elliptic to ovate-elliptic, acute to obtuse, 3- or 5-veined. Petals unguiculate, claw free part ca one to two mm long; blade 4.5–8 mm long, 2.1–4 mm wide, obliquely triangular, apically connivent with dorsal sepal, 2-veined, veins branching, short ciliate only on external margins. Lateral sepals 4.5–9 mm long, 3.3–6 mm wide, oblique, broadly ovate, obtuse, occasionally connivent in natural position to give a bidentate synsepal effect, 5-veined. Lip unguiculate, claw two mm long, semiterete, apically somewhat expanded giving an appearance of narrow triangle; lamina 2.5–3 mm long, 2.5–5 mm wide, at right angle with claw, 3-lobed apically, obtriangular-obovate in outline from cuneate base, apex truncate, concave-conduplicate, glabrous; the middle lobe linear-lanceolate, acute; lateral lobes triangular, erect, obtuse. Gynostemium three mm long, massive, shortly stalked (Fig. 18).

*Ecology*: Terrestrial. Flowering in March, May, and August.

*Distribution*: Peru, Ecuador, Colombia, Venezuela. Alt. 2,000–3,200 m.

*Representative specimens:* COLOMBIA. **Cauca**: Popayán, *H. Karsten s.n*. (W!, UGDA!—drawing); **Nariño**: Pasto, *E. Klaboch s.n*. (W!, UGDA!—drawing); **Tolima**: Machín, Quindio Trail. Forest, 2,000–2,500 m, 3 Aug. 1922, *E.P. Killip & T.E. Hazen 9576* (AMES!, NY!, US!, UGDA!—drawing) (Fig. 13).

*Other materials examined:* PERU. **Cajamarca**: Cajabamba, 2,600–2,700 m, 16 Mar. 1948, *R. Ferreyra 3046* (AMES!, UGDA!—drawing). ECUADOR. **Pichincha**: Lloa, SE of Quito, entering by of the old road Quito-San Juan-Chiriboga-Santo Domingo, 2,400 m, May 1985, *A. Hirtz 2584* (RPSC!, UGDA!—drawing); Mt. Pasochoa, cerca de Alóag, carretera Quito-Machachi, 2,900–3,200 m, 1 May 1985, *C. Dodson & A. Hirtz 15819* (AMES!, QCA!, QCNE!, RPSC!, UGDA!—drawing); Pifo, Quito-Baeza, 2,800 m, Mar. 1984, *A. Hirtz 1594* (RPSC!); Pichincha in dumetis interandibus proep Pifo, *P. Mille 26* (AMES!—drawing); Quito, *H. Karsten s.n*. (W!); Quito, *W. Jameson s.n*. (W!). VENEZUELA. Mérida, *J. Linden 1470* (K!, G). UNPRECISE LOCALITY: Silvia, *F.C. Lehmann BT72* (NY!).

*Notes*: *P. parvilabris* is almost similar to *P. lilacina*, but the lip of this species is devoid of any thickened plates.

***Ponthieva vallecaucana*** Szlach. Kolan. & Olędrzyńska, sp. nov. TYPE: Colombia, *E.L. Core 1472* (holotype, US-2105659!, UGDA!—drawing; isotype, USF-26644!).
(urn:lsid:ipni.org:names:xxxxxxx-x)
*The new entity is similar to P. parvilabris, but unlike in this species the leaves are long-petiolate, petals are obliquely oblong-ligulate, with rounded apices, lip is shortly unguiculate, lamina ecallose, with a pair of large, triangular calli along the claw, broadly fused with the base of the gynostemium*.

**Figure 19:**
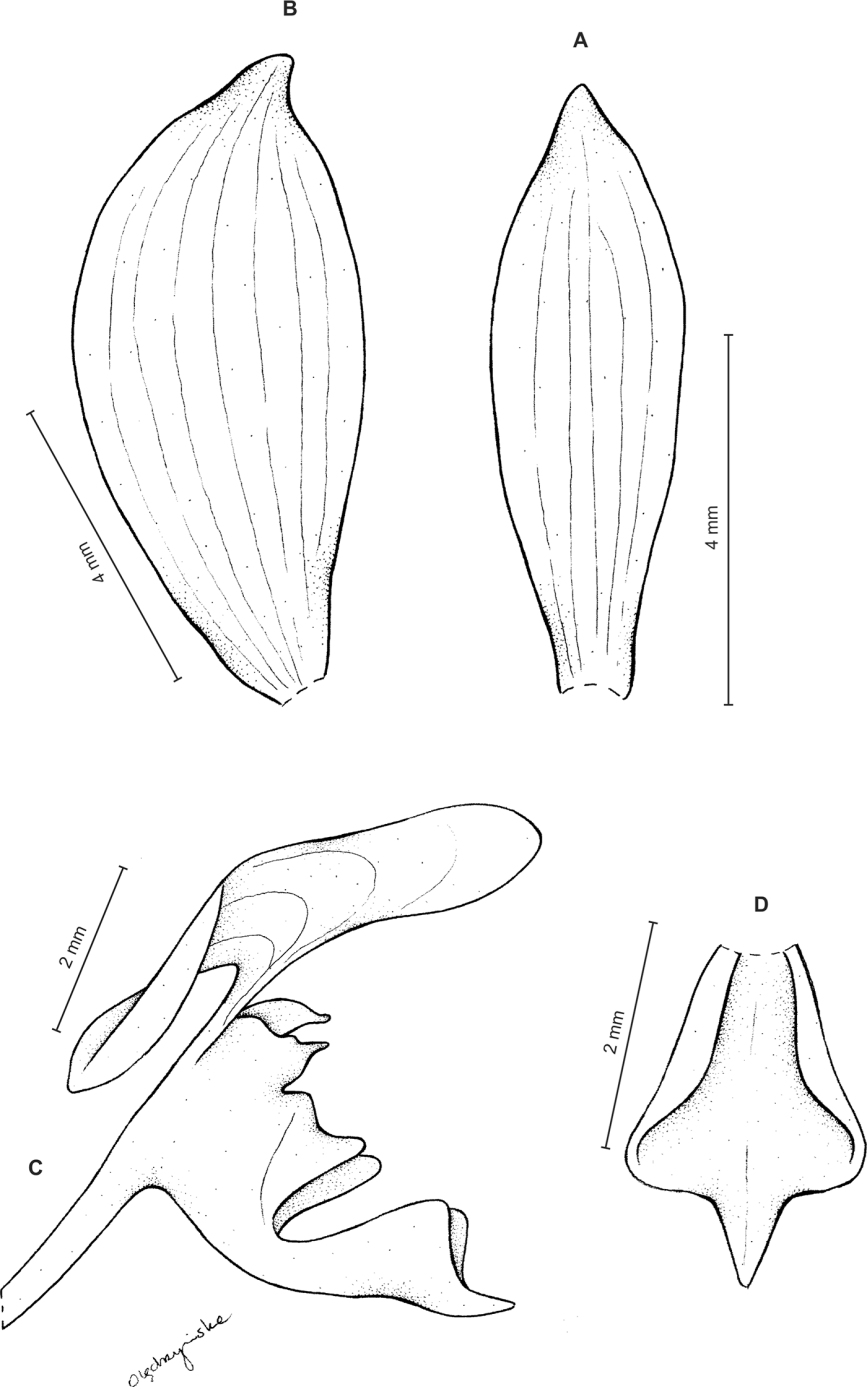
*Ponthieva vallecaucana* Szlach. Kolan. & Olędrzyńska. (A) Dorsal sepal, (B) lateral sepal, (C) petal, gynostemium and lip, (D) lip, front view. Drawn by N. Olędrzyńska from *Core 1472*.

Plants up to 35 cm tall. Leaves 3–7, petiolate; petiole up to six cm long; blade up to eight cm long, four cm wide, glabrous. Peduncle delicate, sparsely pubescent-glandular, loosely 3-sheathed, terminated by a laxly 10–20-flowered raceme six cm long. Flowers medium-sized. Brown at the base, tipped with bright yellow. Floral bracts eight mm long, densely glandular-pubescent. Pedicellate ovary 15 mm long, densely pubescent-glandular. Dorsal sepal nine mm long, two mm wide, narrowly elliptic, obtuse, 5-veined. Petals unguiculate, blade six mm long, 1.4 mm wide, obliquely oblong-ligulate, apically connivent with dorsal sepal, 2-veined, veins branching, short ciliate only on external margins. Lateral sepals 10.5 mm long, four mm wide, free to the base, obliquely broadly elliptic-obovate, obtuse, 7-veined. Lip shortly unguiculate, claw margins expanded into triangular, erect lobes; lamina three mm long, two mm wide, 3-lobed apically, obtriangular-obovate in outline from cuneate base, ecallose; middle lobe lanceolate, subacute; lateral lobes triangular-ovate, obtuse. Gynostemium ca 5.5–6 mm long, stalked, much swollen above fusion with lip (Fig. 19).

*Etymology*: In reference to the place of collection of the type specimen.

*Ecology*: Terrestrial. Flowering in October.

*Distribution*: Colombia. Alt. 1,380–1,500 m.

*Representative specimen*: COLOMBIA. **Valle del Cauca**: Near El Queremal, 1,380–1,500 m, 27 Oct. 1944, *E.L. Core 1472* (US!, UGDA!—drawing) (Fig. 13).

*Notes*: The new species is rather similar to *P. parvilabris*, but its leaves are long-petiolate (petiole up to six cm long vs. 0.5–1(3) cm long), petals are obliquely oblong-ligulate (vs. obliquely triangular), with rounded apices, lip is sessile (vs. lip claw two mm long), with a pair of large, triangular calli at the base broadly fused with the base of the gynostemium (vs. lip glabrous).

**3. *Diptera* group**

Plants with basal, petiolate, glabrous leaves, raceme glandular or pubescent, petals unguiculate, lip with reduced blade, with various form of calli, gynostemium long-stalked, slender.

## Key to the Species

1. Lip lamina shorter than the long, narrow claw*P. garayana*
1* Lip lamina longer than short claw or sessile2
2. Petals with twisted auricle*P. diptera*
2* Petals with nontwisted auricle*P. elata*

*Ponthieva garayana* Dodson & Vasquez, Icon. Pl. Trop., ser. 2, 4: pl. 365. 1989. TYPE: Bolivia, *R. Vasquez, C. Luer & A. Besse 822* (holotype, MO; isotype, LPB).

**Figure 20:**
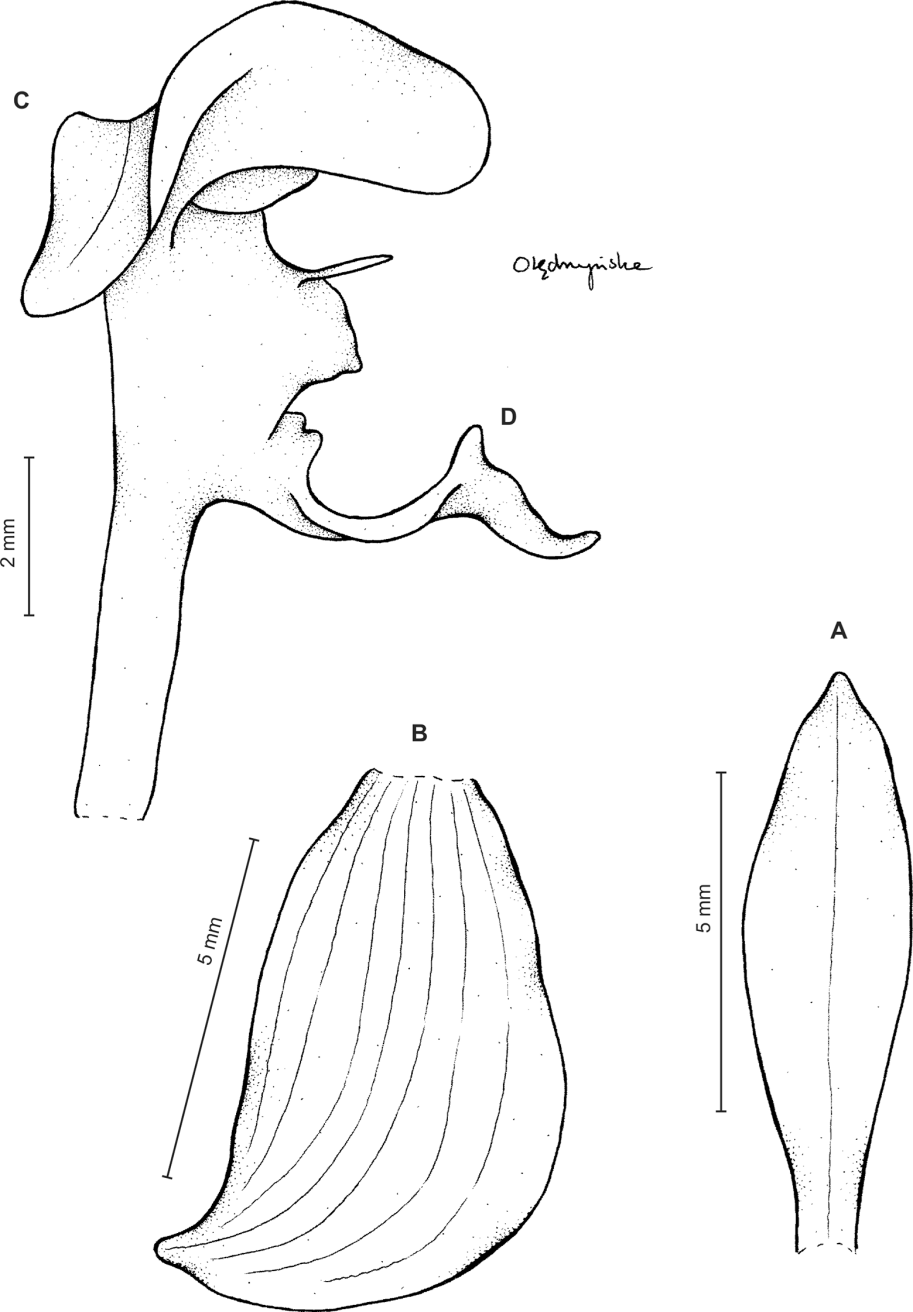
*Ponthieva garayana* Dodson & Vasquez. (A) Dorsal sepal, (B) lateral sepal, (C) petal and gynostemium, (D) lip. Drawn by N. Olędrzyńska from *Fosberg 19983*.

Plants 25 cm tall. Leaves 2–3, basal, rosulate, petiolate; petiole five cm long, cuneate; blade up to five cm long and 2.5 cm wide, elliptic-lanceolate, acute, margins undulate, dark green with four white stripes. Peduncle glabrous, with 3–4 sheaths, terminated by a loosely 1–12-flowered, pulverulent raceme. Flower small, sepals greenish maroon, lateral ones with a yellow spot, petals yellow, lip yellow with green spots. Floral bracts up to six mm long, ovate, acute. Pedicellate ovary up to nine mm long. Sepals pubescent on the outer surface. Dorsal sepal seven mm long, two mm wide, oblong lanceolate, subobtuse, 3-veined. Petals unguiculate, claw free part ca one mm long; blade four mm long, 0.5 mm wide, obliquely oblong ligulate, with prominent basal auricle, obtuse, yellow. Lateral sepals seven mm long, four mm wide, obliquely elliptic-ovate, subacute, 3-veined. Lip long unguiculate, claw longer than lamina, narrow, terete; lamina three mm long, ca one mm wide in total, oblong-lanceolate, acute, concave, basally subcordate. Gynostemium 4.5 mm long, clavate, very long-stalked (Fig. 20).

*Ecology*: Terrestrial in upper montane wet forest. Flowering in February and December.

*Distribution*: Bolivia, Colombia. Alt. 1,800–2,000 m. Reported from Peru by Zelenko & Bermúdez (2009).

**Figure 21:**
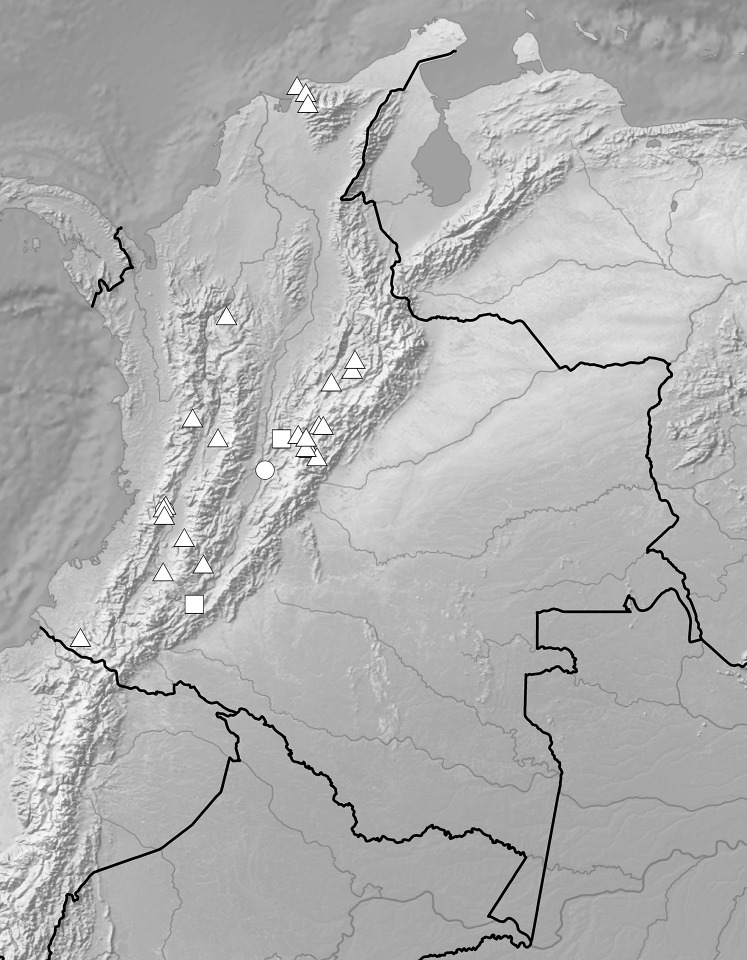
Distribution of representatives of *Diptera*-group. *P. diptera* (triangle), *P. elata* (circle), *P. garayana* (square). Map generated in ArcGis 9.3 (Esri, Redlands, CA, USA).

*Notes*: While examining herbarium material deposited in P and US cited above, we came across specimens which overall fit description of *P. garayana*, but the plants are larger with somewhat larger flower segments, but with slightly smaller lip. We did not include, however, their measurements to the above compilation. The most characteristic features of *P. garayana* are long-stalked gynostemium, long-unguiculate, very small, oblong-lanceolate lip with cordate base, and petals with large basal auricle.

*Representative specimens*: COLOMBIA. **Cundinamarca**: Alto del Trigo, 2,000 m, Dec. 1860, *J. Triana 574* (P!, UGDA!—drawing). **Huila**: Ridge between drainage of Rio Guarapas and Rio Guachicas, just below main ridge of Cordillera Oriental, above Palestina, SW of Pitalito, 1°35′N 76°16′W, 1,800–1,900 m, 6–7 Feb. 1943, *F.R. Fosberg 19983* (US!, UGDA!—drawing) (Fig. 21).

*Ponthieva diptera* Linden & Rchb.f., Bonplandia 2: 278. 1854; TYPE: Colombia, *L.J. Schlim 987* (lectotype, designated by Garay (1978: 216), W!; isolectotypes, AMES-00083531!, AMES-00103516!, AMES-00103517!,, P-00363609!, G, AMES!—drawing, UGDA!—drawing, US!—photo, MO!—photo).
*Ponthieva dicliptera* Rchb.f., Flora 69: 548. 1886. TYPE: Colombia, *F.C. Lehmann 1860* (lectotype, designated by Garay (1978: 216), W!).

**Figure 22:**
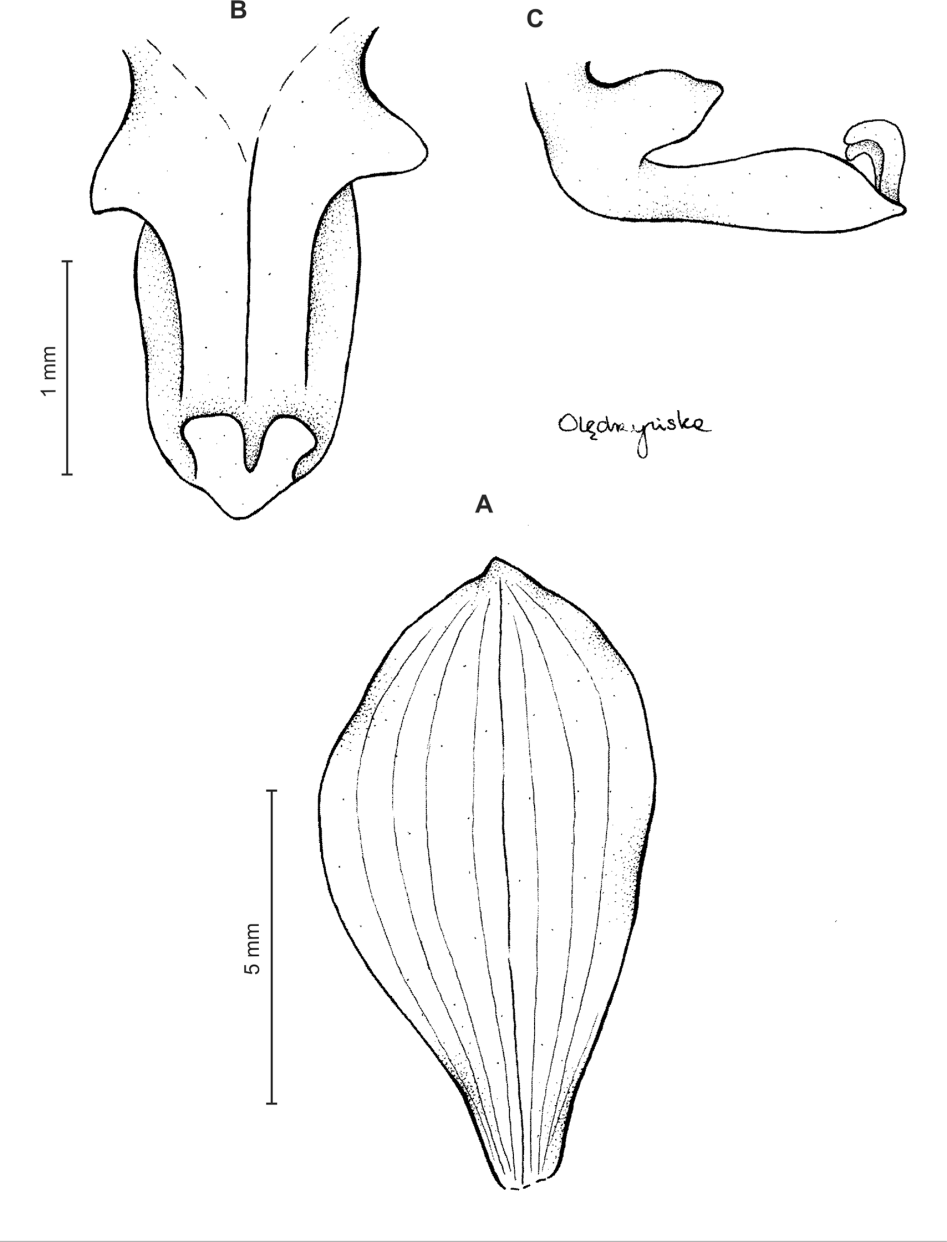
*Ponthieva diptera* Linden & Rchb.f. (A) Lateral sepal, (B) lip, front view, (C) lip, side view. Drawn by N. Olędrzyńska from *Schlim 987*.

**Figure 23:**
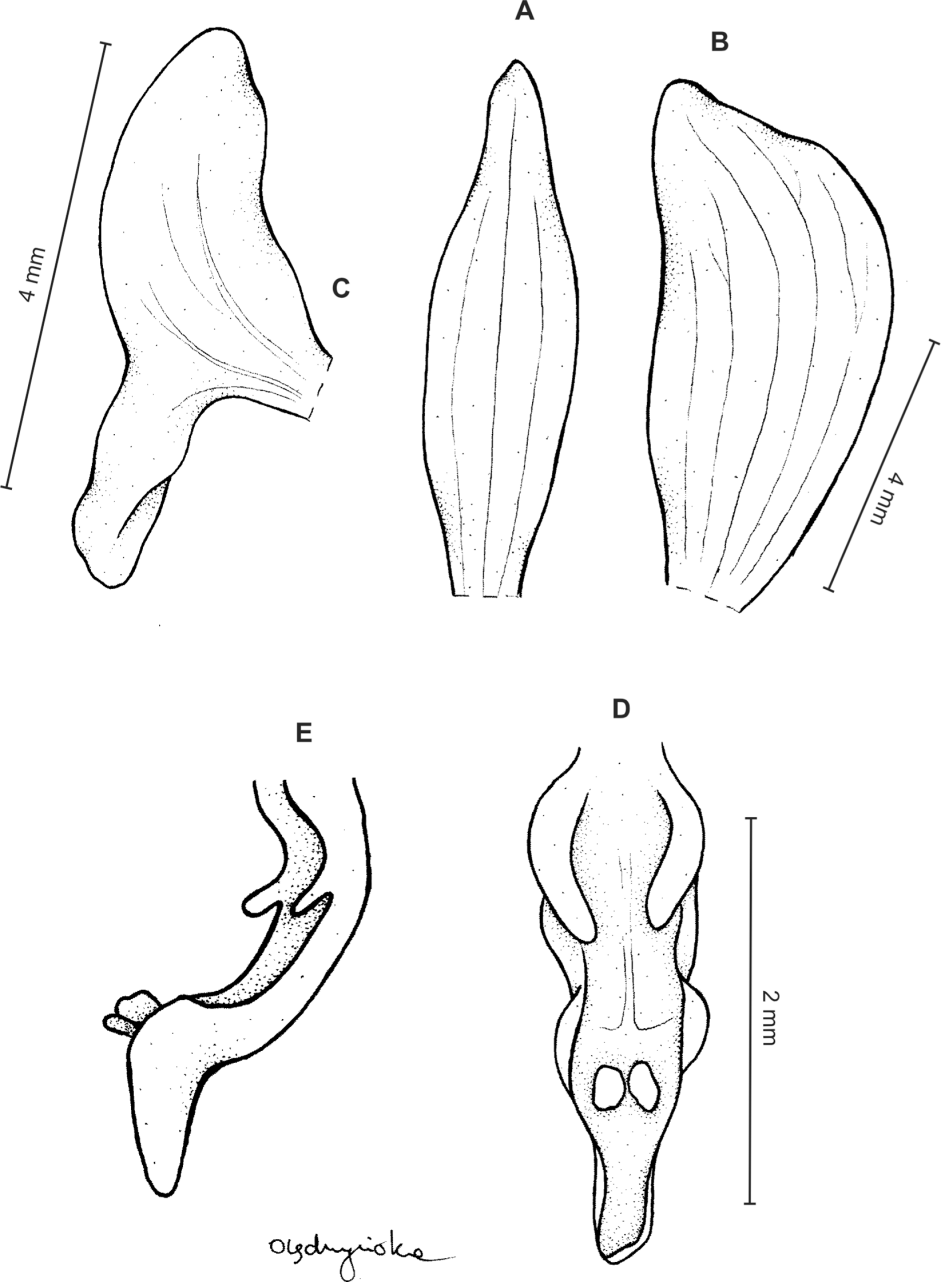
*Ponthieva diptera* Linden & Rchb.f. (A) Dorsal sepal, (B) lateral sepal, (C) petal, (D) lip, front view, (E) lip, side view. Drawn by N. Olędrzyńska from *Camara Leret 11*.

Plants up to about 95 cm tall. Leaves 3–10, clustered at or near the base, long-petiolate; petiole up to 11 cm long, channeled; blade up to 13 cm long and 5.7 cm wide, ovate or ovate-lanceolate to elliptic, acute or acuminate, more or less cuneate below, often oblique, glabrous. Peduncle erect, very sparsely pilose below, more densely above, provided with about 3 distant, tubular sheaths of which the lowest is foliaceous, terminated by a loosely or densely several- to many-flowered raceme about 7–25 cm long. Flowers carmine and yellow, sepals glandular-pubescent. Floral bracts 8–15 mm long, lanceolate, ovate-lanceolate or elliptic-lanceolate, acuminate, glandular-pubescent. Pedicellate ovary 11–15 mm long, glandular-pubescent. Dorsal sepal 7.5–10 mm long, 1.5–3 mm wide, linear-lanceolate to oblong lanceolate, acute to acuminate, 3- or 5-veined. Petals unguiculate, claw free part ca one mm long; blade four to eight mm long in total, 1–2.5 mm wide, obliquely ovate-elliptic to ligulate-ovate, rounded to obtuse at apex, elongate basally into a long narrowly ligulate, blunt and somewhat twisted auricle, veins 2, branching. Lateral sepals 8–11 mm long, 4–5.2 mm wide, obliquely elliptic-obovate to elliptic, acute, several-veined. Lip unguiculate; claw less than one mm long, with a prominent, bilobed callus at base; lamina ca two to three mm long in total, up to 1.3 mm wide, lanceolate, thick, with a prominent bidentate, upcurved callus below apex, apex hooked, ovate to lanceolate, attenuate, obtuse to subacute. Gynostemium up to four mm long, clavate, long-stalked (Figs. 22 and 23).

*Ecology*: Terrestrial in humid forest. Flowering throughout the year.

*Distribution*: The Greater Antilles (Cuba, Jamaica), Peru, Ecuador, Colombia, Venezuela. Alt. 1,350–3,000 m. The occurrence of this species in Ecuador was reported by Garay (1978: 217) and from Venezuela by Carnevali et al. (in Hokche, Berry & Huber, 2008).

*Representative specimens:* COLOMBIA. **Antioquia**: Mpio. Yarumal/Valdivia. Vereda el Cedro. Sitio Alto de Ventanas, 7°05′N 75°32′W, 1,710–1,980 m, 11 Jul. 1992, *A. Gomez & al. 678* (MO!, UGDA!—drawing); **Boyacá/Santander**: Corregimiento de Virolin. Finca La Sierra, 2,500–2,600 m, 13 May 1976, *G. Lozano C. & al. 2461* (COL!); **Cauca**: Bei San Rutonico de Cali, 2,000 m, 4 Mar. 1884, *F.C. Lehmann 3618* (US!, UGDA-LSz!—drawing); Gargantilla Ridge, near Tacueyo, Rio Palo basin, Huila group, Cordillera Central, 2,100 m, Jan. 1906, *H. Pittier 1008* (AMES!, US!, UGDA!—drawing); Popayán. Vangobio[?], 1,700–2,000 m, Jan. 1886, *F.C. Lehmann 10039* (AMES!, NY!, UGDA!—drawing); Monte del Diablo. Cerca de La Ceja, 21 Jul. 1944, *Hno. Daniel s.n*. (COL!); **Boyacá**: Mpio. de Moniquirá. Vereda Corolado, sector alto. Rio Pomeca. Bosque subandino, 2,200–2,300 m. 9 Jul. 2001, *H. Dueñas 3082* (COL!); **Cundinamarca**: Guasca, El Salitre, Reserva Biologica El Encenillo, Bosque nublados andinos maduros dominados por *Weinmannia tomentosa* y *Drymis granatensis*, 2,900–3,200 m, 27 Feb. 2009, *R. Camara Leret 11* (COL!, UGDA!—drawing); Carretera entre Subachoque y La Pradera, vertiente oriental, 3,000 m, 24 Nov. 1956, *M. Ospina H. 43* (AMES!, UGDA!—drawing); Mpio. del Suesca, Vereda de Hato Grande. 7.3 km al SE del caserio, 3,200 m, 19 Dec. 1963, *C. Saravia T. & G. Lozano C. 3143* (COL!); Bogotá, Quebrada del Chico, 2,800 m, 20 Feb. 1944, *M. Schneider 208/1* (COL!); Represa Muña, 2,800 m, 19 Jan. 1998, *H. Ospina M. 1499* (COL!); Usaquén, subparamo. Matorral subserial del bosque altoandino con *Weinmannia, Vallea stipularis, Oreopanax mutisiana* y *Hesperomeles*, 3,050 m, 20 May 1972, *A. Cleef 3923* (COL!); Paramo de Usaquén, 2,800–2,900 m, 6 Jun. 1952, *M. Schneider 208/1* (COL!); Usaquén, 2,900–3,000 m, 6 Jun. 1943, *M. Schneider 208/3* (COL!); Carretera entre Subachoque y La Pradera, vertiente Oriental, 3,000 m, 24 Nov. 1956, *M. Ospina H. & J.M. Idrobo 43a* (COL!); Quebrada del Chico, Bogotá, 20 Feb. 1944, *M. Schneider 208/1* (COL!); Snia, Sisga, 2,850 m, 3 Oct. 2003, *M. Ospina H. 1586* (COL!); **Magdalena**: Santa Marta, 1898–1901, *H.H. Smith 2496* (AMES!, F!, MO!, P!, US!, UGDA!—drawing); Sierra Nevada de Santa Marta. Cerro Quenado trail, above Hacienda Cincinnati, 1,500–2,300 m, 28 Aug. 1935, *G.W. Martin 3778* (AMES!, MO!); Sierra Nevada de Santa Marta. In forest on ridge W of Quebrada Botella along trail to San Pedro de la Sierra, 1,900–2,200 m, 6 Oct. 1972, *J. Kirkbride 2403* (COL!, NY!, US!); **Nariño**: Mpio. de Barbacoas, Corregimiento Altaquer. Vereda El Barro. Reserva Natural Rio Nambi, vertiente occidental andina. Bosque pluvial premontano, bosque primario poco intervenico, margen derecha delo Rio Nambi, 1°18′N, 78°08′W, 1,325 m, 2 Dec. 1993, *J. Betancur & al. 4369* (COL!—sterile); **Risaralda**: Mpio. de Pueblo Rico. Vereda Monte Bello. Potreros de La Playa. En immediaciones del Rio Taiba, 5°13′35″N, 76°05′01″W, 1,500 m, 1 Oct. 2006, *R. Arevalo & al. 691* (COL!); Mpio. de Santa Rosa de Cabal, Finca El Cortijo, 4°51′N, 75°32′W, 2,300 m, 22 Sep. 1988, *A. de Wilde 2854* (COL!); *Sine loc., L.J. Schlim 987* (W!), *Sine loc., F.C. Lehmann 1869* (W!), *Sine loc*. (Schlechter, 1920); **Santander**: Mpio. de Charalá. Corregimiento de Virolin. Margen del Rio Luisito, 1,650 m, 1 Dec. 1978, *S. Diaz P. & P.M. Ruiz 1430* (COL!, UGDA!—drawing); **Valle del Cauca**: Mpio. Cali. San Antonio, west of Cali, near summit of Cordillera Occidental, 1,900–2,350 m, 26 Feb–2 Mar. 1939, *E.P. Killip & H. Garcia 33906* (AMES!, COL!, US!). Mpio. La Cumbre, 1,700–2,200 m, 11, 16 Sep. 1922, *E.P. Killip 11349* (AMES!, US!); Finca Zingara. Km 18 de la carretera Cali-Buenaventura. Km 4 via de Dapa, corregimiento de la Elvira, Cordillera Occidental, 3°30′N 76°34′W, 1,900 m, 3 Apr. 1994, *J. Giraldo-Gensini & C. Espinosa 253* (MO!); San Antonio, W of Cali. Near summit of Cordillera Occidental, 1,900–2,350 m, Feb–Mar 1939, *E.P. Killip & H. Garcia 33906* (COL!). *Sine loc., F.C. Lehmann BT74* (NY!, UGDA!—drawing) (Fig. 21).

*Other materials examined*: PERU. **Cusco**: La Convención. Doist. Santa Teresa, cerro Yerbabuenayoc, 13°07′35″S 72°36′52″W, 2,400 m, 23 Mar. 2004, *I. Huamantupa & A. Huamantupa 4341* (MO!).

*Notes*: This is the most widespread species of the genus known in the study area and a morphological variation between populations is observed (Figs. 22 and 23). However, *P. diptera* can be easily distinguished from other species by presence of the obliquely ovate-elliptic petals which are basally elongate into long narrowly ligulate, blunt and somewhat twisted auricle as well as by the narrow, thick lip adorned with two digitate projections at base and unequally 3-dentate apex. Garay (1978: 217) synonymized under this name *P. elata* from Colombia. According to Schlechter (1920) and this study, however, *P. elata* can be easily separated from *P. diptera* by having non-twisted auricle of petal and lip with different calli. Calli consists of a single, acute, upcurved horn near the apex of the lip and unlobed calli at its base.

*Ponthieva elata* Schltr., Repert. Spec. Nov. Regni Veg., Beih. 7: 63. 1920. TYPE: Colombia, *M. Madero s.n*. (B†); NEOTYPE (here designated): COLOMBIA. **Cauca**: *Sine loc*. Alt. 1,900 m. *M. Madero 169* (B†, AMES-00103521!—drawing).

**Figure 24:**
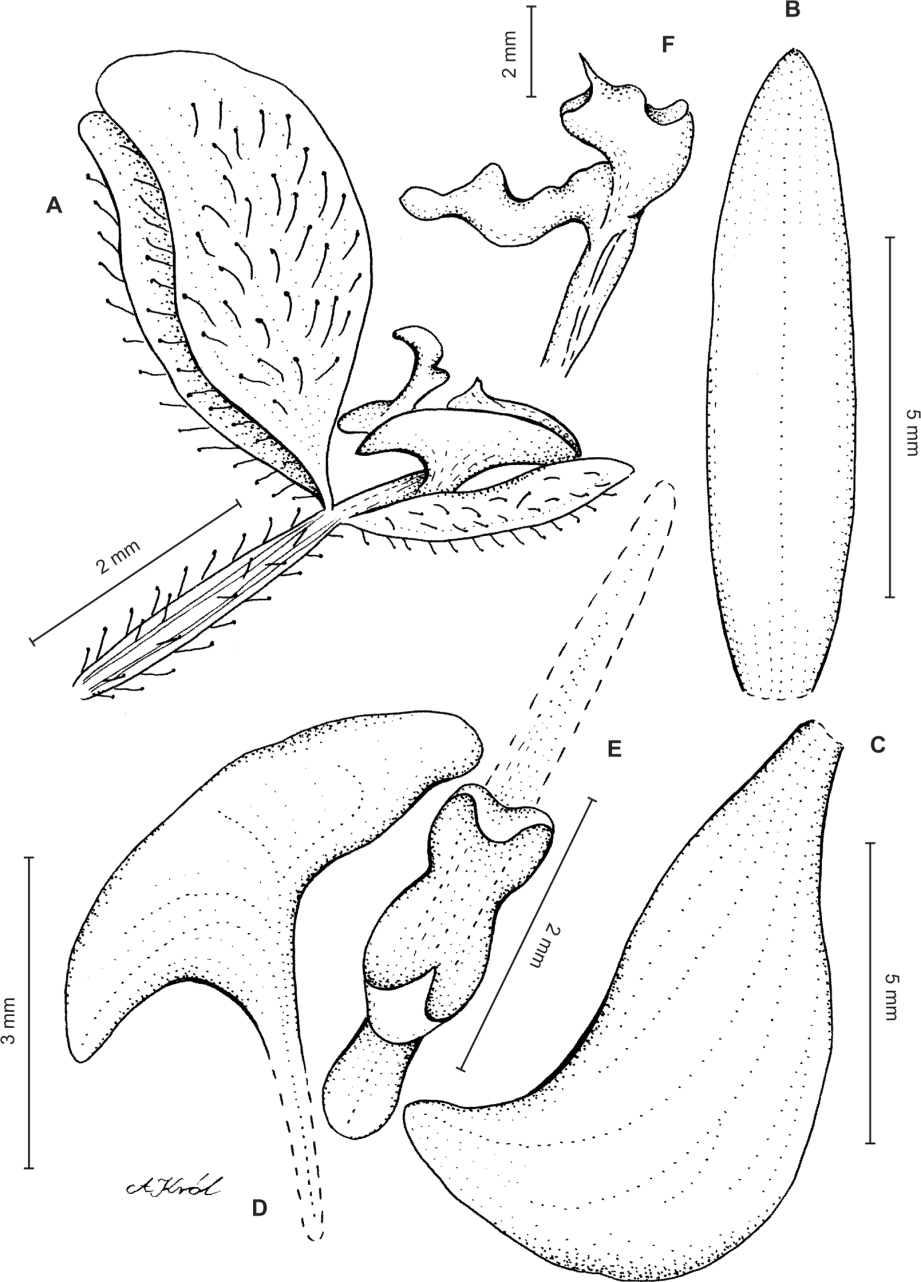
*Ponthieva elata* Schltr.—original Schlechter’s illustration reproduced by Mansfeld (1929) and redrawn by A. Król. (A) Flower, (B) dorsal sepal, (C) lateral sepal, (D) petal, (E) lip, (F) gynostemium. Scale bars made based on measurements provided by Schlechter in species description.

Plants 50–75 cm tall. Leaves 2–5, basal, petiolate, glabrous; petiole 5–11 cm long, canaliculate; blade 5.5–14 cm long, 2.5–4.5 cm wide, obliquely elliptic to elliptic-lanceolate, acuminate. Peduncle erect, glandular towards the apex, 2–4-sheathed, terminated by a subdensely many-flowered raceme up to 15 cm long. Flowers medium-sized, sepals rather sparsely glandular. Floral bracts 11–14 mm long, oblong, subacute, densely glandular. Pedicellate ovary 14–25 mm long, densely hairy-glandular. Dorsal sepal 7.2–9 mm long, 1.5–2 mm wide, oblong-ligulate, blunt, 5-veined. Petals unguiculate; claw free part ca 0.5 mm long; lamina five mm long, 1.5–2 mm wide, transversely elliptic-reniform or dolabriform-triangular, obtuse to blunt at both ends, veins branching from the base of the free part. Lateral sepals 8–10.5 mm long, 2.5–3 mm wide, elliptic-obovate, apically strongly falcate, obtuse, free to the base or basally connate, 5-veined, lateral veins more or less branching. Lip shortly unguiculate or subsessile; lamina 2.5–4.5 mm long, up to 1.8 mm wide, oblong-pandurate in general outline, apex ligulate, rounded to subacute, below apex prominent hook-like, upcurved appendage, at the base of disc prominent thickening directed toward the apex. Gynostemium 2–2.5 mm long, rather robust, shortly stalked (Fig. 24).

*Ecology*: Terrestrial in humid montane forest. Flowering in April.

*Distribution*: Colombia. Alt. 1,900–2,100 m.

*Representative specimens:* COLOMBIA. **Cauca**: *Sine loc*., 1,900–2,000 m, *M. Madero s.n*. (B†); *Sine loc*. Alt. 1,900 m. *M. Madero 169* (B†, AMES!—drawing); **Cundinamarca**: Ricaurte, *F.C. Lehmann BT73* (NY!, UGDA!—drawing); **Valle del Cauca**: Mpio. Yumbo. Dapa, Jorge Negret farm, 35 min from el Rodadero station, 2,074 m, 3°32′54.9″N 76°35′15.4″W, Apr. 2012, *E. Parra s.n*. (VALLE!) (Fig. 21).

*Notes*: According to Schlechter (1920) this species differs from similar *P. diptera*, by the lip and petals form. Garay (1978: 217), however, considered these two species as conspecific. The lip of *P. elata* is ornamented near the apex with hook-like, upcurved appendage, while in *P. diptera* the apical callus is distinctly bidentate.

There is a drawing of *Ponthieva elata* made under Schlechter’s supervision of *Madero 169* collection in the AMES herbarium. Schlechter (1920), however, mentioned in the protologue *Madero s.n*.

**4. *Mandonii* group**

Plants with petiolate, glabrous leaves, finely glandular-pubescent raceme, small inconspicuous flowers, with broadly ovate to transversely rhombic, long-unguiculate lip with dendritic venation, obscurely 3-lobed at the apex, with a transverse, triangular, cucullate callus at the base, and subsessile gynostemium.

*Ponthieva mandonii* Rchb.f., Xenia Orchid. 3: 18. 1878. TYPE: Bolivia, *G. Mandon 1164* (lectotype, here designated, W-50403!; isolectotypes, AMES-00103540!, G, GH-00103541, GOET-008658, HBG-506873, K-000573754, LE-00006489, MO, NY-00009281, P-00363563!, S-07-7263, W!, AMES-00103542!—photo, drawing, UGDA!—drawing).

**Figure 25:**
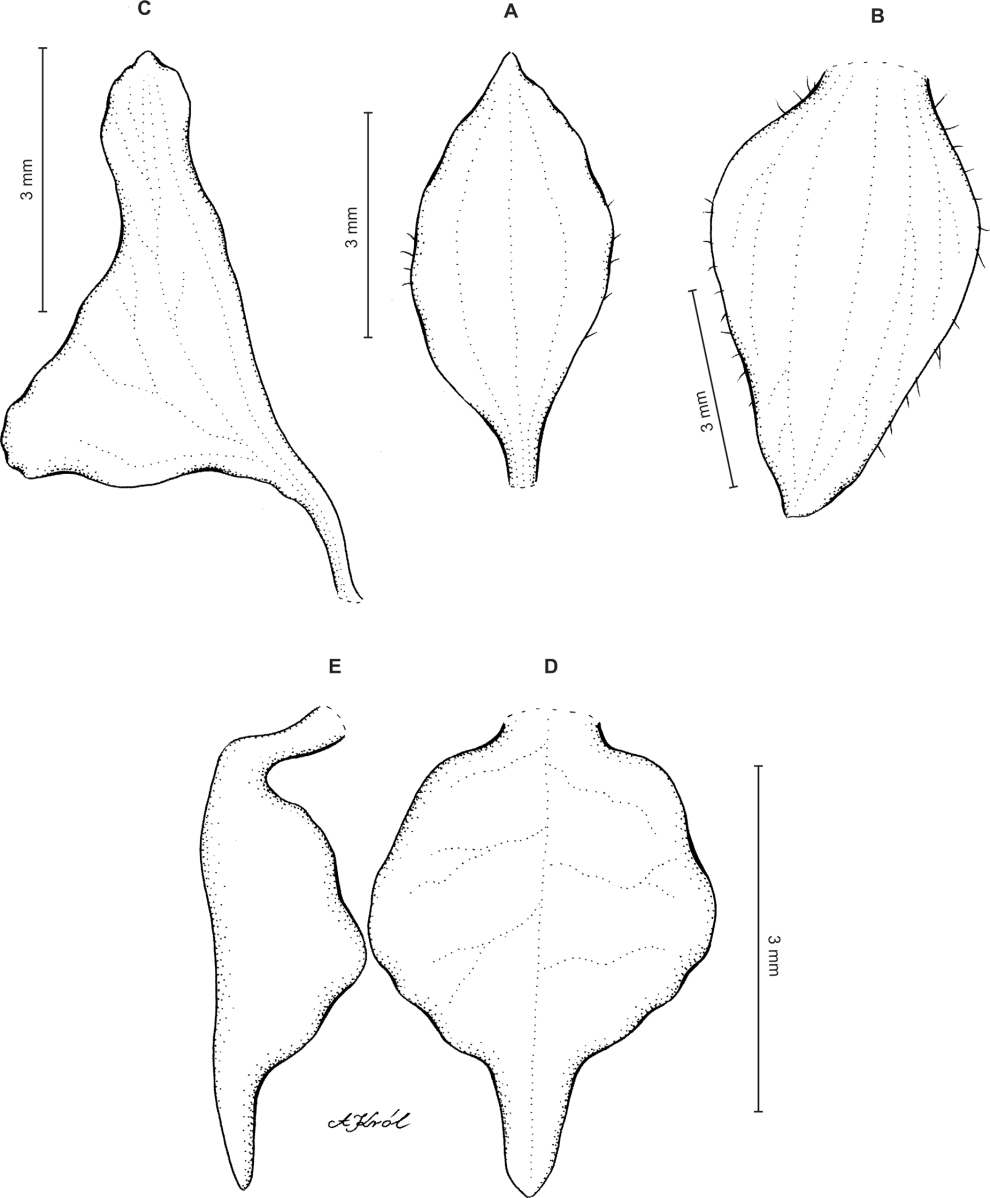
*Ponthieva mandonii* Rchb.f. (A) Dorsal sepal, (B) lateral sepal, (C) petal, (D) lip, front view, (E) lip, side view. Drawn by A. Król from W-R 50403.

Plants up to 25 cm tall. Leaves 3–6, basal, rosulate, petiolate; petiole ca one cm long; blade 3.5–6 cm long and up to 2.5 cm wide, elliptic to oblong-elliptic, acute, more or less narrowed below. Peduncle slender, glabrous below, finely glandular-pubescent above, provided with few (1–3) remote tubular sheaths, terminated by a loosely to subdensely few- to several-flowered raceme rarely up to eight cm long. Flowers small, white, with the ovary and sepals (especially the lateral ones) densely glandular-tomentose within. Floral bracts five mm long, ovate to ovate-lanceolate, sparsely glandular. Pedicellate ovary 12 mm long, densely glandular. Dorsal sepal about 5.8–7 mm long, ca 2.3–3 mm wide, elliptic-lanceolate or ovate-elliptic, acute or acuminate, 3- or 5-veined. Petals unguiculate, claw free part ca 1.5 mm long; blade five to six mm long, 2.5–3 mm wide, very obliquely oblong-triangular, rounded or obtuse at the apex, with the outer margin concave and more or less irregular, vein 1, basally branching. Lateral sepals seven mm long, four mm wide, free to more or less connivent, ovate to ovate-elliptic or ovate-lanceolate, acute or obtuse, veins 5, branching. Lip unguiculate, claw up to two mm long; lamina up to five mm long and three mm wide, broadly ovate to transversely rhombic, obscurely 3-lobed at the apex, concave-conduplicate in natural position, vein with dendric branches, with a transverse, triangular, cucullate callus at the base; the middle lobe narrowly ligulate. Gynostemium two to three mm long, subsessile (Fig. 25).

*Ecology*: Terrestrial. Flowering in May and August.

*Distribution*: Argentina, Bolivia, Peru, Ecuador, Colombia. Alt. 1,600–3,500 m. The occurrence of this species in Peru was reported by Zelenko & Bermúdez (2009).

**Figure 26:**
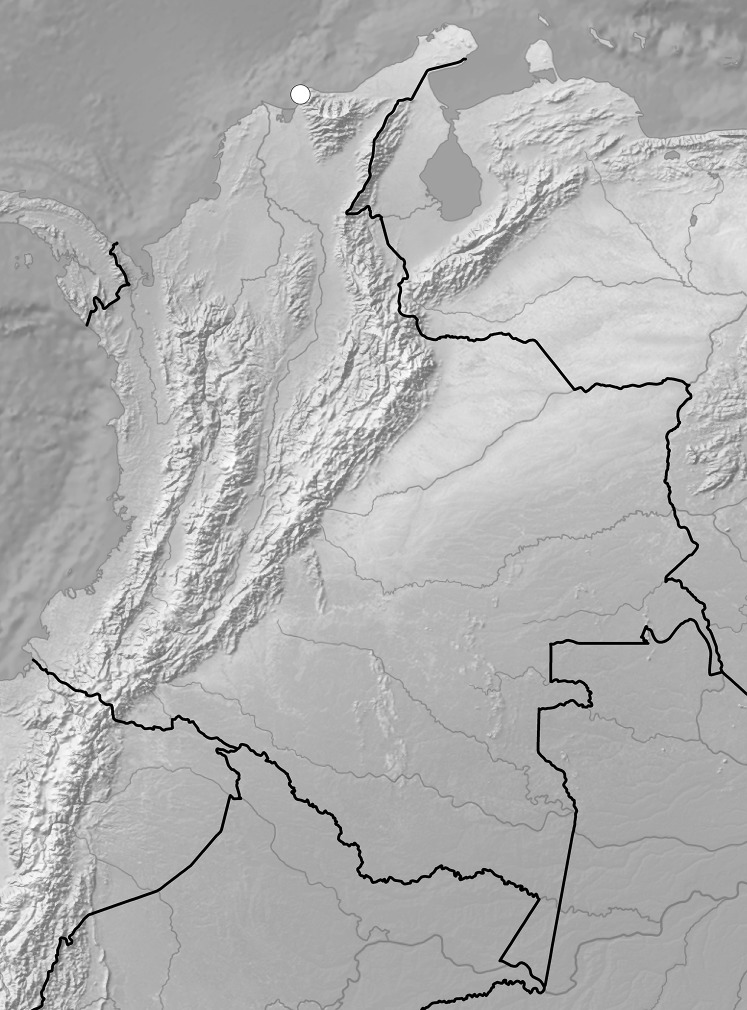
Distribution of *P. mandonii* (circle). Map generated in ArcGis 9.3 (Esri, Redlands, CA, USA).

*Representative specimens:* COLOMBIA. **Cauca**: *Sine loc*. (Schlechter, 1920: 216, *ex* Kraenzlin); **Magdalena**: Sta Marta, *J. Goudot s.n*. (P!, UGDA!—drawing) (Fig. 26).

*Other materials examined*: ECUADOR. Chimborazo, Canon of the Rio Chanchan, about five km N of Huigra. Moist forested valley in the afternoon fogbelt, 1,600–2,150 m a.s.l., 19–29 May 1945, *W.H. Camp E-3411* (AMES!, COL!, MO!, QCNE!); Cuenca. Bei Mari finca, 3,500 m a.s.l.. 16 Aug. 1878, *Sine coll. s.n*. (W!). BOLIVIA. Larecaja. Sorata, cerro del Immapi, 2,650 m a.s.l., *G. Mandon 1164* (W!).

*Notes*: According to Schweinfurth (1958), *P. mandonii* possesses a transverse, triangular, cucullate callus at the base of the lip lamina, but we were not able to observe it in the material we examined. This species is easily separable from any other *Ponthieva* species known from NW part of South America by its lip form with very unique dendritic venation. We found similar lip form in Colombian material cited above, but this specimen differs from the type of *P. mandonii* in having connate lateral sepals and falcate elliptic-ovate petals.

**5. *Maculata* group**

Leaves densely villose or pubescent throughout, including leaves. Flowers often showy, with sessile to subsessile lip with small blade, and variously developed calli. Gynostemium usually elongate, rather slender, long-stalked.

## Key to the Species

1. Petals with basal auricle provided with a short, lobe-like protuberance overlapping blade*P. similis*
1* Petals without protuberance2
2. Leaves margins densely hirsute, blade sparsely hirsute*P. cesarensis*
2* Leaves margins and blade evenly pubescent or villose3
3. Leaves shorter than inflorescence, both sides of basal thickenings long decurrent towards the middle of lip disc*P. maculata*
3* Leaves as long as or longer than inflorescence, both sides of basal thickenings abruptly decurrent on lip disc*P. villosa*

*Ponthieva maculata* Lindl., Ann. & Mag. Nat. Hist., ser. 1, 15: 385. 1845. TYPE: Colombia, *K.T. Hartweg s.n*. (lectotype, designated by Garay (1978: 217), K-L-000079990!).

*Ponthieva wallisii* Rchb.f., Linnaea 41: 101. 1876. TYPE: Colombia, *G. Wallis s.n*. (lectotype, designated by Garay (1978: 217), W-50380!; AMES-00103575!—drawing; UGDA!—drawing).

**Figure 27:**
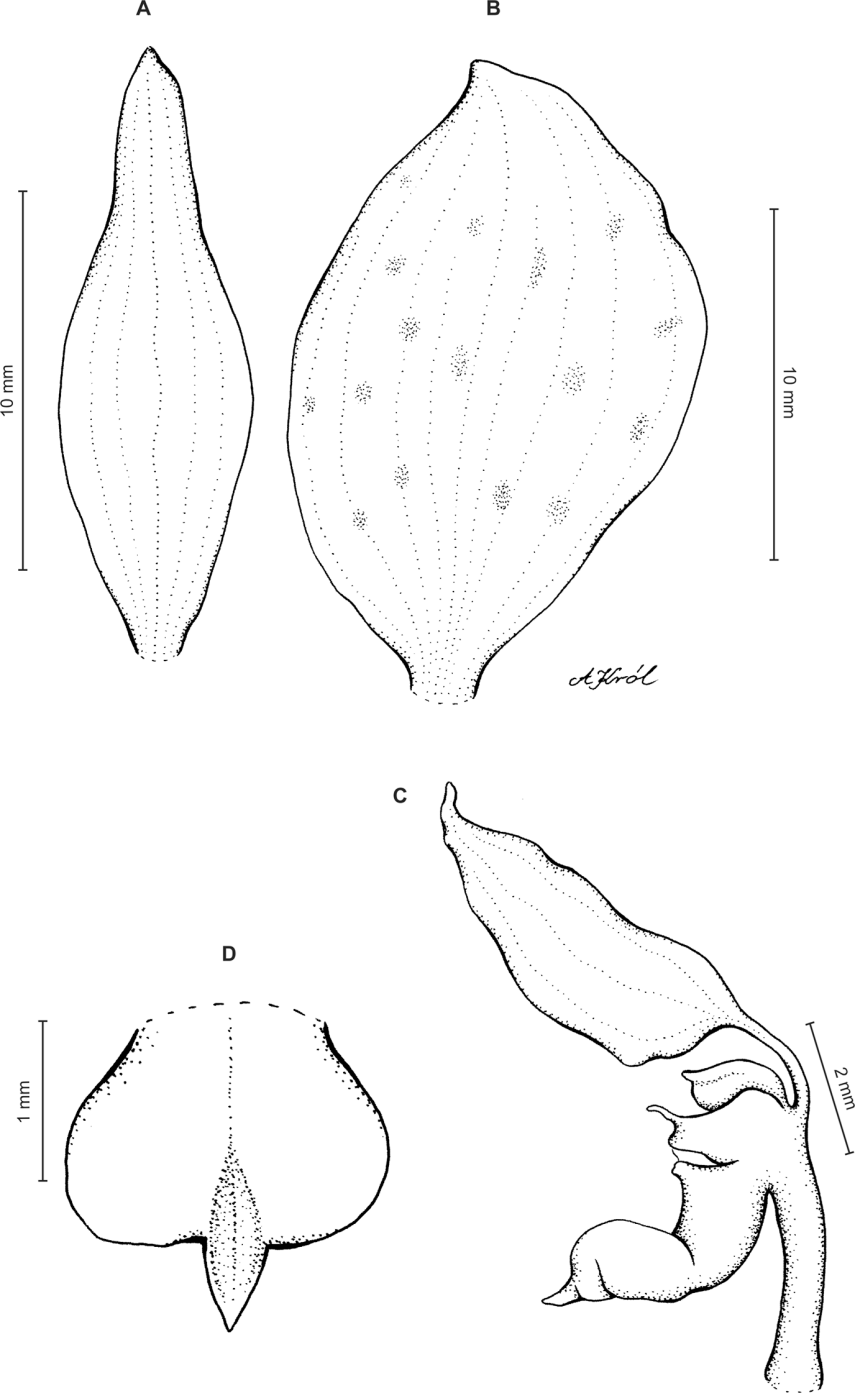
*Ponthieva maculata* Lindl. (A) Dorsal sepal, (B) lateral sepal, (C) petal, lip and gynostemium, (D) lip, front view. Drawn by A. Król from *Schlim 1020*.

**Figure 28:**
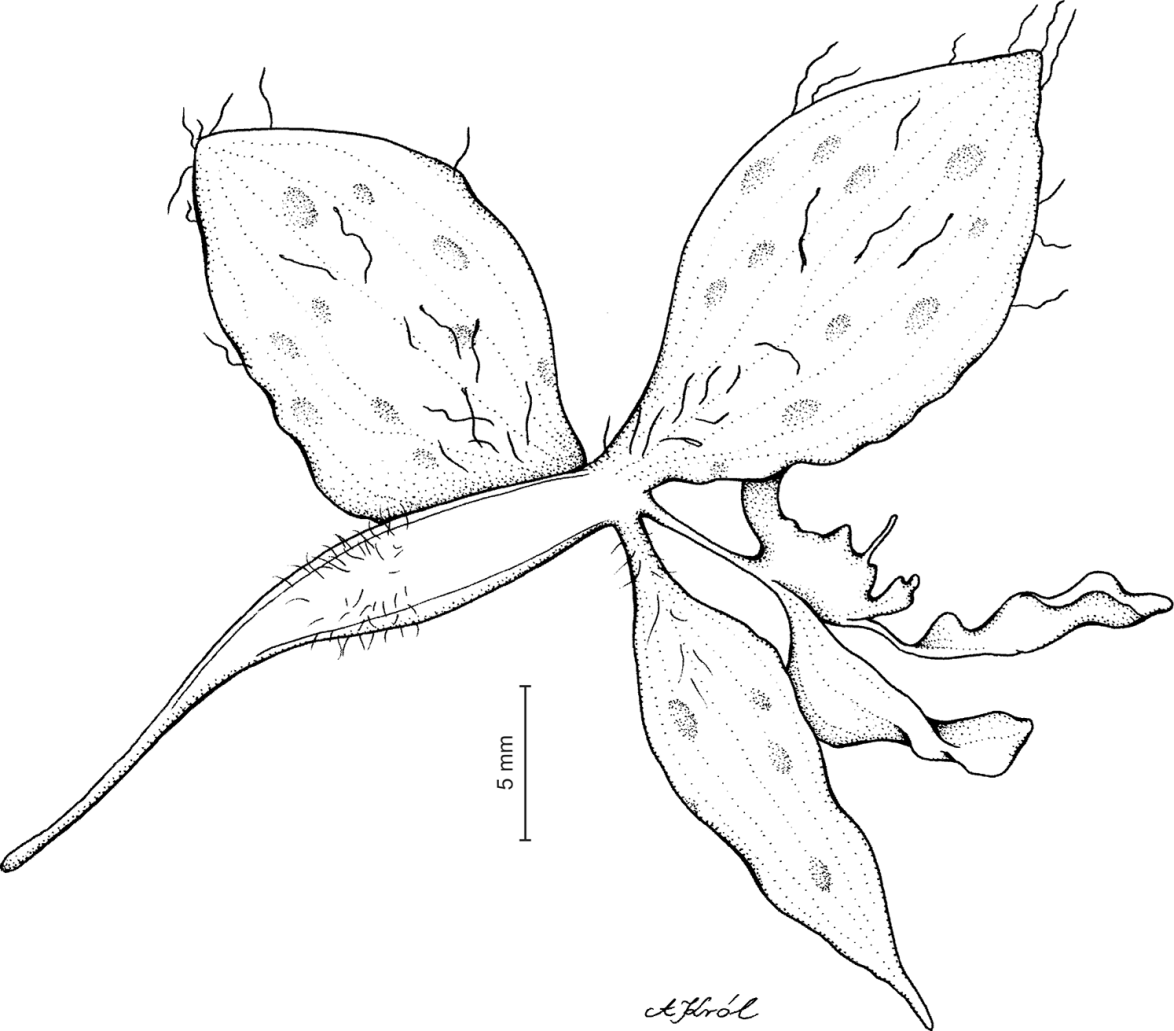
*Ponthieva maculata* Lindl.—flower. Drawn by A. Król from *Wallis s.n*.

Plant up to about 50 cm tall. Leaves 2–4, basal, erect to suberect, conduplicate; blade up to 37 cm long and five cm wide, lanceolate, elliptic-lanceolate, or oblanceolate, acute to abruptly acuminate, gradually tapering to a sessile base, densely pubescent. Peduncle erect or slightly arcuate, with one or two sheaths, villose throughout, terminated by a loosely many-flowered raceme. Flowers rather large for the genus, yellow-green with brownish to purplish veins and spots, sepals villose on the outer surface. Floral bracts up to 12 mm long, erect, lanceolate, cucullate, acute or shortly acuminate. Pedicellate ovary up to 27 mm long, delicate. Dorsal sepal up to 16 mm long and four mm wide, narrowly elliptic, concave, acute, 3- or 5-veined, veins branching or simple. Petals unguiculate; claw free part two to three mm long; blade up to 12 mm long and three mm wide, dolabriform to oblong lanceolate, obtuse, 1-veined, basally branching, margins glabrous. Lateral sepals up to 16 mm long and nine mm wide, free to the base, spreading, broadly elliptic to obovate, slightly oblique, acute or obtuse, sparsely papillose within, 3- or 5-veined, veins branching or simple. Lip up to 4.2 mm long and two mm wide, sessile, reddish green to orange in color, light pink in dried condition, conduplicate-cymbiform, abruptly acuminate, when expanded cuneate in outline, truncate in front, apiculate in the middle, fleshy, basally provided with a transverse, grooved plate, both sides of which are long-decurrent towards middle of disc. Gynostemium ca five to seven mm long, clavate, stalked, stalk ca (2.5) 4–5 mm long (Figs. 27 and 28).

*Ecology*: Terrestrial in montane forest. Ames & Correll (1952) reported also epiphytic populations from Guatemala. Flowering throughout the year.

*Distribution*: Mexico, Guatemala, El Salvador, Honduras, Nicaragua, Costa Rica, Panama, Ecuador, Colombia, Venezuela. Alt. 1,900–3,000 m.

**Figure 29:**
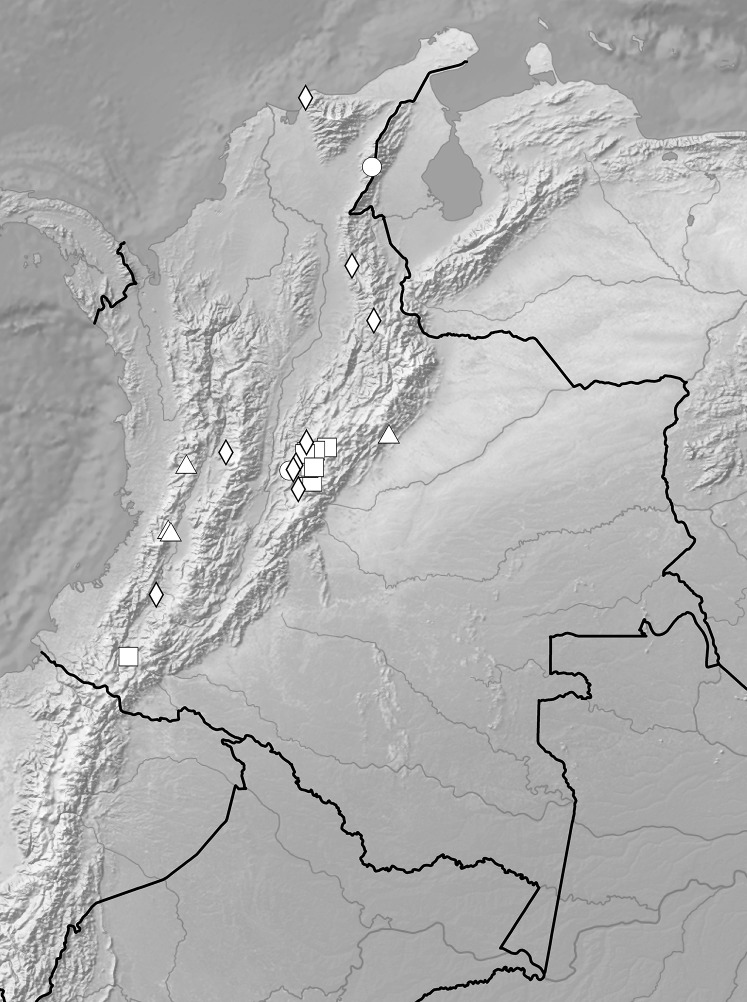
Distribution of representatives of *Maculata*-group. *P. cesarensis* (circle), *P. maculata* (diamond), *P. similis* (square), *P. villosa* (triangle). Map generated in ArcGis 9.3 (Esri, Redlands, CA, USA).

*Representative specimens:* COLOMBIA. **Caldas**: Pinares. Above Salento. Cordillera Central, 2,800–3,000 m, 2–10 Aug. 1922, *F.W. Pennell 9245* (AMES!, NY!, US!); **Cauca**: Road from El Tambo to 20 de Julio (vertiente oriental), ca 55 km, 2,100 m, 27 Jan. 1976, *T. Plowman & J. Vaughan 5309* (COL!); El Tambo, Parque Nacional Natural Munchique, alrededores de la Cabana de la Romelia, dentro de la Parcela Fenologica #1, 2,600–2,700 m, 12 Apr. 1994, *F. Gonzalez & al. 3136* (COL!); **Cundinamarca**: Mpio. de Bojacá, vereda San Antonio, La Merced, proximo a la carretera Mosquera-Tena, 2,600–2,700 m, 19 Mar. 1964, *J.H. Torres & G. Lozano C. 59* (COL!); Tena, *K.T. Hartweg s.n*. (K-L!); Bei Pasca, 2,000–2,300 m, 4 Feb. 1883, *F.C. Lehmann 2517* (W!); Veragua grande and Pacho, 1,900–2,300 m, Dec. 1892, *F.C. Lehmann 7301* (AMES!, NY!, W!); **Magdalena**: Santa Marta, Sierra del Libano. Mountain forest, 2,300 m, 21 Jun. 1899, *H.H. Smith 2359* (AMES!, F!, NY!, P!, US!, UGDA!—drawing); **Norte de Santander**: Ocaña, *Bruckmuller s.n*. (W!); Ocaña, *G. Wallis s.n*. (W!); Ocaña, *Schlim 1020* (W!, UGDA!—drawing); Ocaña, 2,000–2,300 m, 1846–1852, *L. Schlim s.n*. (P!); Pamplona, 2,500 m, 1847, *Funck & Schlim 1443* (P!, W!); **Santander**: S slope of Mt San Martín, near Charta, dense woods, 2,300–2,500 m, 10 Feb. 1927, *E.P. Killip & A.C. Smith 19184* (AMES!, NY!, US!, UGDA!—drawing); *Sine loc., J.W.K. Moritz & E. Klaboch 380* (W!) (Fig. 29).

*Other materials examined:* ECUADOR. **Zamora-Chinchipe**: Cruz Grande, 15 km N of Valladolid, 2,400 m, Oct. 1986, *D. Dalessandro 740* (RPSC!, UGDA!—drawing). VENEZUELA. Caracas, 1842, *J. Linden 140* (P!—4, W!). UNPRECISE LOCALITY. (New Granada) La Baja, 1847, *L. Schlim 1440* (W!).

*Notes*: Foldats (1969) synonymized under this species two Central American taxa—*P. formosa* Schltr. and *P. brenesii* Schltr., while Garay (1978: 217) synonymized *P. wallisii* Rchb.f. In our opinion, however, *P. brenesii* is rather conspecific with *P. villosa*, characterized below, as both species have inflorescence shorter than leaves. Both other aforementioned taxa seem to be conspecific with *P. maculata*.

*Ponthieva maculata* is very similar to *P. villosa*. According to Garay (1978) both species can be distinguished by vegetative characters, that is, *P. maculata* are larger, robust plants (vs. plants rather small in *P. villosa*), leaves are shorter than inflorescence (vs. leaves as long as or longer than inflorescence), raceme elongate, many-flowered (vs. short, few-flowered), as well as some flower parts, that is, lip twice longer than wide (vs. as long as wide), and connection between lip basal plate with disc. In *P. maculata* both sides of plates are long-decurrent toward the middle of disc, whereas in *P. villosa*—they are abruptly decurrent on disc. To the aforementioned differences we can add length of the gynostemium stalk—it is long, ca four to five mm, in *P. maculata*, and ca two mm long in *P. villosa*. Further study should reveal whether these characters warrant proper demarcation of both species.

*Ponthieva villosa* Lindl., Pl. Hartw.: 155. 1845. TYPE: Ecuador, *K.T. Hartweg s.n*. (lectotype, designated by Garay (1978: 229), K-L!; AMES-00083549!—photo).
*Ponthieva brenesii* Schltr., Repert. Spec. Nov. Regni Veg., Beih. 19: 165. 1923. TYPE: Costa Rica, *A.M. Brenes 83* (B†; lectotype, designated by Barringer (1986: 18), CR-26246; isolectotypes, AMES-00062183!, NY-9277, F—photo).

**Figure 30:**
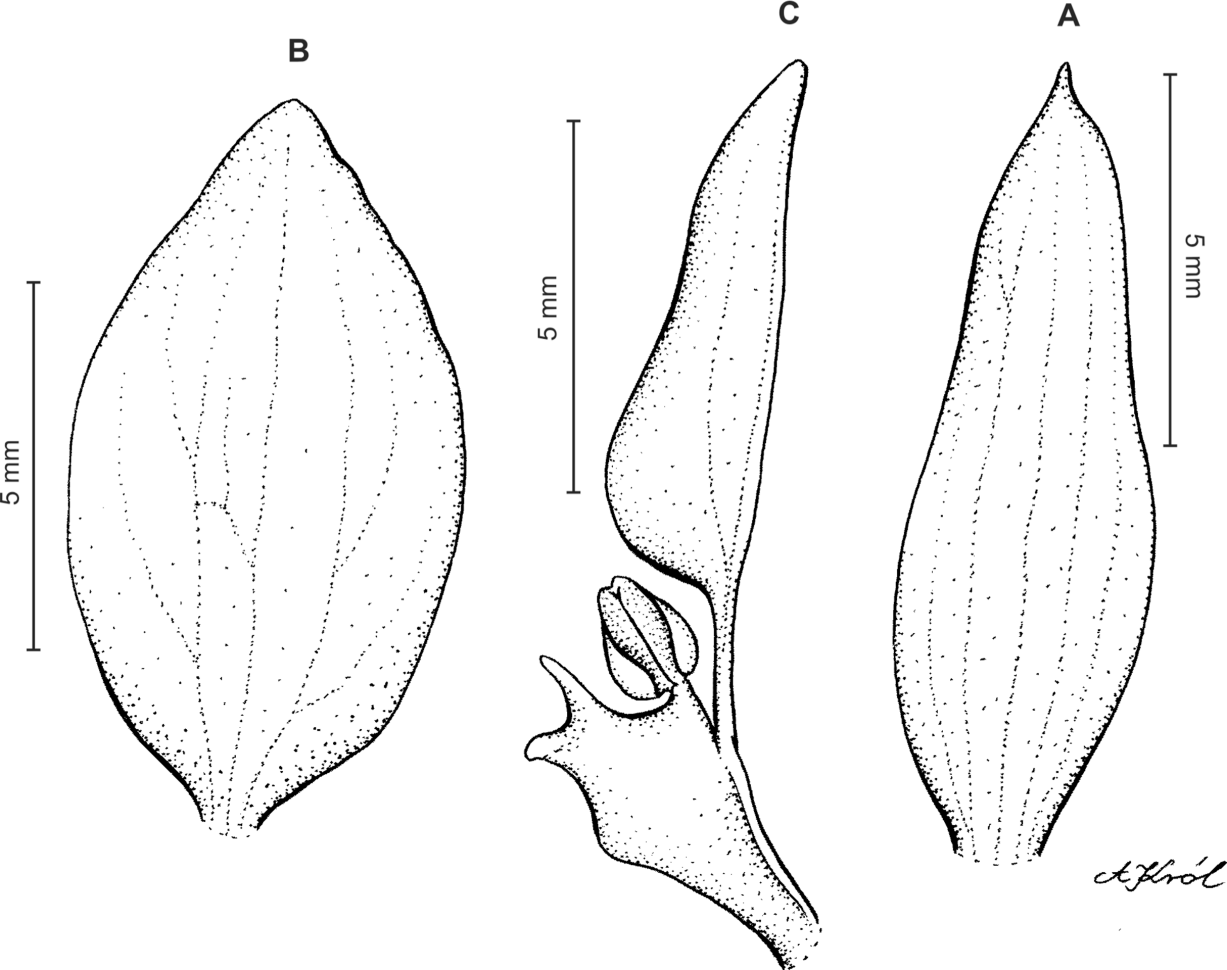
*Ponthieva villosa* Lindl. (A) Dorsal sepal, (B) lateral sepal, (C) petal and gynostemium. Drawn by A. Król from W-R 50425.

**Figure 31:**
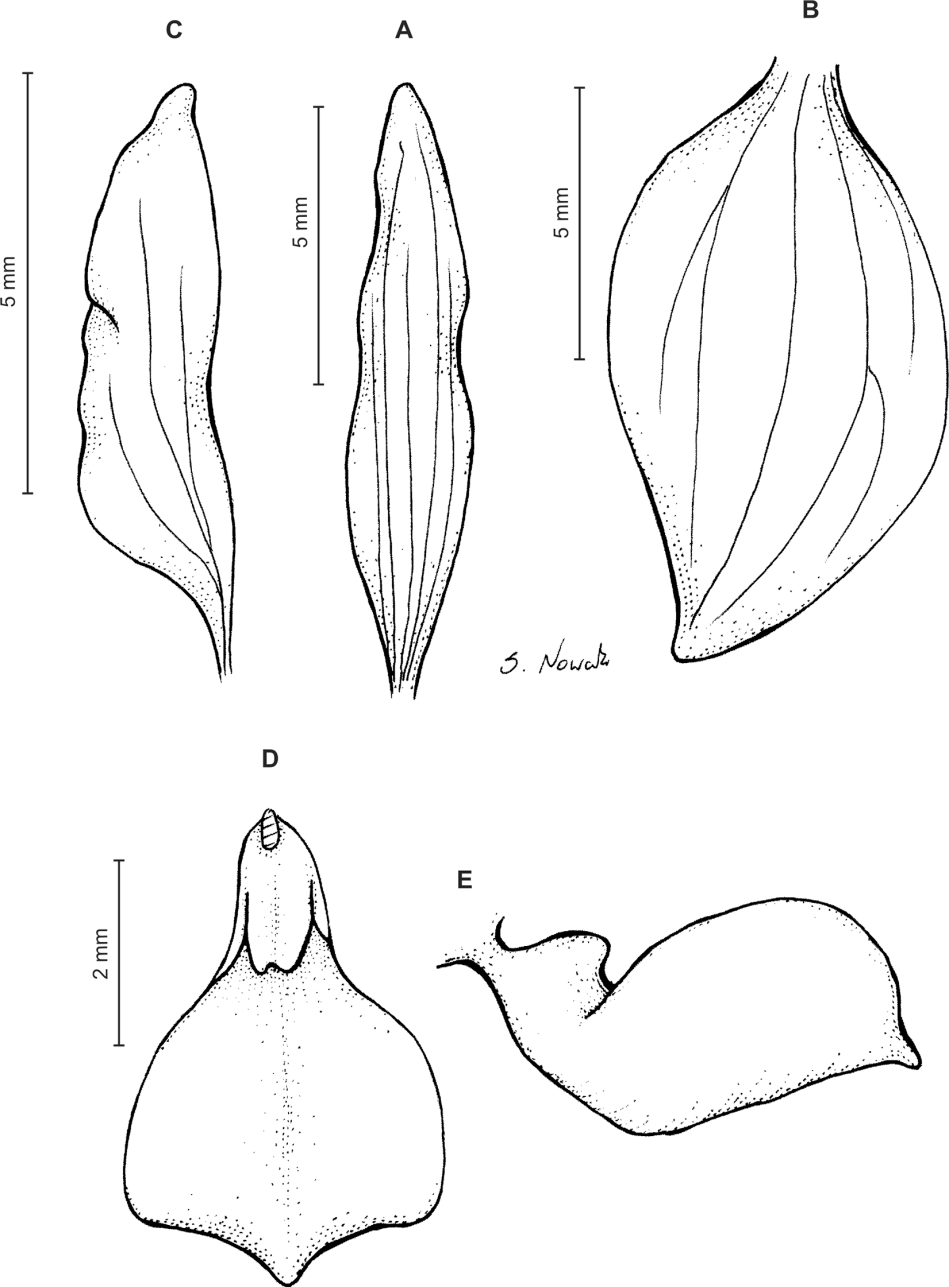
*Ponthieva villosa* Lindl. (A) Dorsal sepal, (B) lateral sepal, (C) petal, (D) lip, front view, (E) lip, lateral view. Drawn by S. Nowak from *Lozano C. & al. 965*.

**Figure 32:**
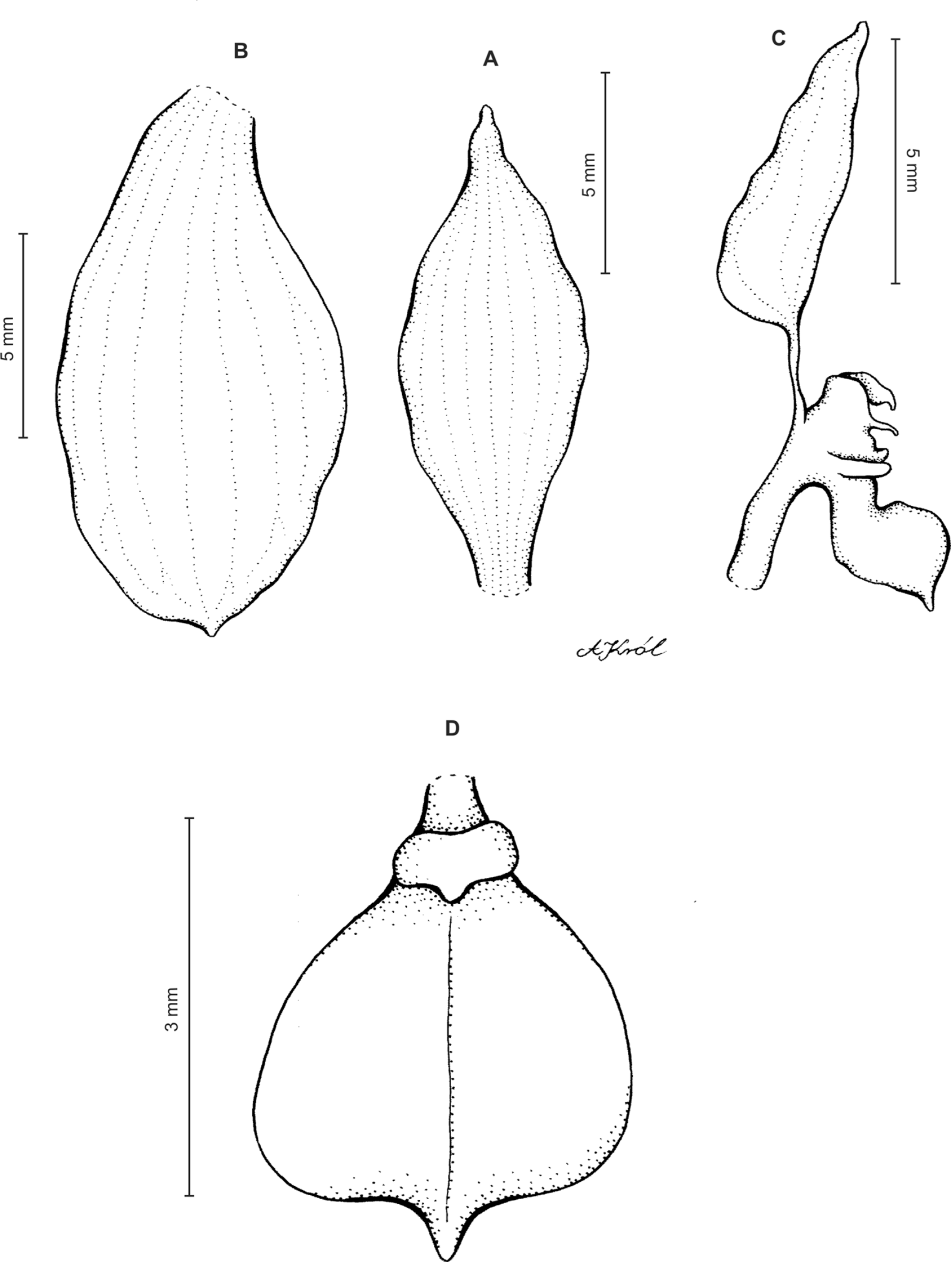
*Ponthieva villosa* Lindl. (A) Dorsal sepal, (B) lateral sepal, (C) petal, lip and gynostemium, (D) lip, front view. Drawn by A. Król from *Warszewicz s.n*.

Plants up to 20 cm tall, but usually shorter. Leaves 3–4, basal, rosulate, petiolate; petiole up to five cm long, narrow, canaliculate; blade up to 20 cm long and 1.7 cm wide, linear-lanceolate to lanceolate, acute, soft, densely villose. Peduncle delicate, villose, 2–3-sheathed, terminated by a laxly 6–10-flowered raceme. Flowers medium-sized, green, apical part of petals white, sepals pubescent-villose on both surfaces. Floral bracts five to seven mm long, lanceolate, acute, villose. Pedicel and ovary up to 27 mm long. Dorsal sepal 10–12 mm long, 2.8–4 mm wide, oblong-ovate to ligulate-oblanceolate, acute, 5-veined. Petals unguiculate; claw free part ca 2–2.5 mm long, narrow; blade six to seven mm long, 1.6–3 mm wide, obliquely oblong-ovate, rather acute at apex, 2-veined. Lateral sepals 10–13 mm long, 5.5–8 mm wide, suborbicular-elliptic, oblique, acute, free, 3-veined, veins branching. Lip 2.5–3.5 mm long and wide when spread, subsessile, obtrullate to subquadrate in general outline, cymbiform, deeply concave in the centre, subacute to subobtuse, basal thickening obscurely bilobed, abruptly decurrent on disc. Gynostemium ca 4.5 mm long, shortly stalked, ca two mm (Figs. 30–32).

*Ecology*: Epiphytic in montane wet forest. Flowering throughout the year.

*Distribution*: Mexico, Guatemala, El Salvador, Honduras, Nicaragua, Costa Rica, Panama, Peru (Zelenko & Bermúdez, 2009), Ecuador, Colombia. Alt. 1,200–2,200 m. Castillo-Campos et al. (2009) reported occurrence of *P. brenesii* from Mexico where it was found at the altitude of about 2,300 m.

*Representative specimens:* COLOMBIA. **Boyacá**: Mpio. de Pajarito, corregimiento de Corinto, 2,200 m, 16 Oct. 1967, *G. Lozano C. & al. 965* (COL!, UGDA!—drawing); **Valle del Cauca**: Mpio. El Cairo. Las Amarillas, frontera Valle-Choco, Cordillera Occidental, Serrania de los Paraguas, carratera destapada El Cairo-Rio Blanco a 1 hora en jeep de El Cairo, 2,070 m, 31 Mar. 1988, *F. Silvesrstone-Sopkin & al. 3830* (CUVC!); Mpio. La Cumbre. Alto Chicoral, detras de Dapa, vertiente Occidental, cabecera del rio Bitaco, bosque de neblina, corregimiento de Bitaco, 2,000 m, 27 Jan. 1989, *J. Ramos 1829* (MO!); Mpio. Yumbo, DAPA, Finca Debusale, 1,900 m, 23 May 2010, *O. Perez & E. Parra 0795* (CUVC!); Mpio. Yumbo. Dapa, Jorge Negret farm, 35 min from el Rodadero station, 2,074 m, 3°32′54.9″N 76°35′15.4″W, Apr. 2012, *E. Parra s.n*. (VALLE!); Cultivated by Orquivalle in El Barranco, San Antonio, 20 Feb. 2011, *D.L. Szlachetko s.n*. (photo) (Fig. 29).

*Other materials examined:* ECUADOR. **El Oro**: *K.T. Hartweg s.n*. (K-L!). COSTA RICA. **Alajuela**: San Pedro de San Ramón, *A.M. Brenes 83* (W!). *Sine loc. J. Warszewicz s.n*. (W!, UGDA!—drawing). **Heredia**: two km W of the branch in the road to Poas Volcano and to Puerto Viejo, 1,900 m, 17 Feb. 1965, *R. W. Lent 362* (COL!).

*Notes*: *Ponthieva crinita* and *P. villosa* share a similar habit, that is, they are rather slender, delicate plants with inflorescences usually shorter or as long as leaves, which is opposite to common *P. maculata*. According to Garay (1978), *P. villosa* has acute petals with branched veins, lip is triangular-ovate, and cuneate at base. Petals of *P. crinita* are obtuse to obliquely rounded with unbranched veins, lip is quadrate in outline, and basally auriculate. We cannot confirm different form of petals as discriminative character separating both species, but we observed differences in lip shape. Unfortunately, we do not have access to sufficient materials of both taxa to formulate definite conclusions concerning their taxonomic status. Barringer (1986) considered *P. brenesii* as synonymous with *P. maculata*.

*Ponthieva cesarensis* Szlach. & Kolan., Pl. Syst. Evol. 299(9): 1675. 2013. TYPE: Colombia, *O. Rivera Diaz & al. 2849* (holotype, COL-522507!, UGDA!—drawing).

**Figure 33:**
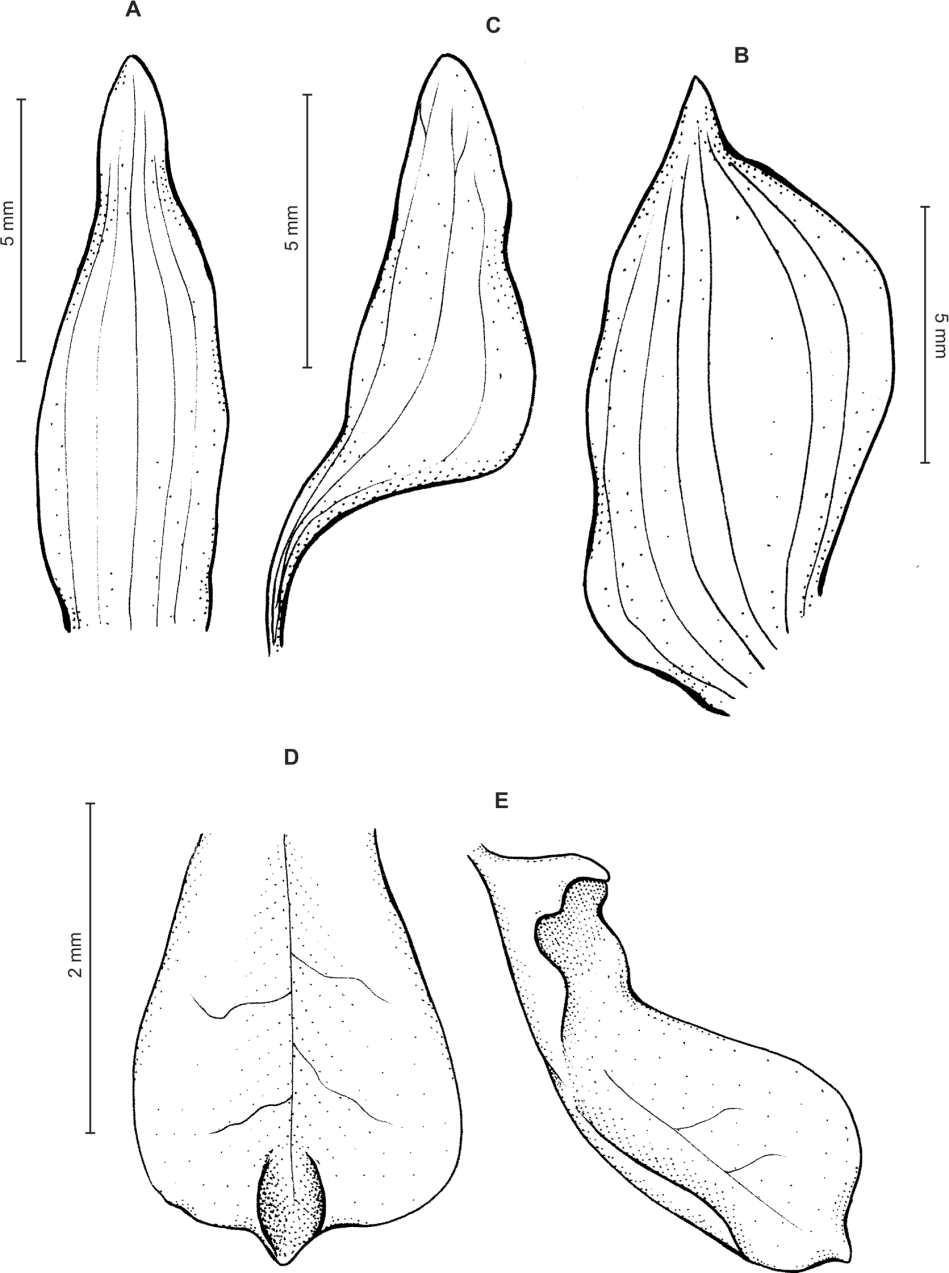
*Ponthieva cesarensis* Szlach. & Kolan. (A) Dorsal sepal, (B) lateral sepal, (C) petal, (D) lip, front view, (E) lip, lateral view. Drawn by P. Baranow from *Rivera Diaz & al. 2849*.

Plants up to 47 cm tall. Leaves 3–4, basal, rosulate, petiolate; petiole up to five cm long, narrow; blade up to 18 cm long and four cm wide, oblanceolate to oblong-oblanceolate, acute, somewhat oblique, margins densely hirsute, blade sparsely hirsute. Peduncle hirsute, 2-sheathed, terminated by a laxly 10-flowered raceme up to seven cm long. Flowers small, sepals green, lip yellow with dark green lines. Floral bracts up to 10 mm long, lanceolate, acute, densely hirsute. Pedicellate ovary up to 17 mm long, densely hirsute. Sepals sparsely hirsute or glabrous. Dorsal sepal up to 11 mm long and 3.2 mm wide, oblong-lanceolate, subacute, 5-veined. Petals unguiculate; claw free part 1.5–2 mm long; lamina up to eight mm long and 2.5 mm wide, cuneate to truncate at base, obliquely ovate-lanceolate, obtuse, veins 3, branching, margins glabrous. Lateral sepals 12 mm long, 6.5 mm wide, free to the base, obliquely elliptic-ovate with lanceolate, acute apex, 6-veined. Lip four mm long, 1.3–2 mm wide, subsessile, oblong-obovate in outline, rounded at apex with small triangular projection at the apex, conduplicate, concave in the center, with single, branching vein, basal callus roof-like, truncate, with sides decurrent abruptly on lip margins. Gynostemium 2.5 mm long, massive, subsessile (Fig. 33).

*Ecology*: Terrestrial in subparamo with vegetation dominated by Asteraceae and Ericaceae, in sandy and rocky soils. It was also found at lower elevations in the vegetation dominated by Anacardiaceae (*Toxicodendron*), Araliaceae (*Oreopanax*), Cunoniaceae (*Weinmannia*), Burseraceae (*Bursera*). Flowering in February, November and December.

*Distribution*: Colombia. Alt. 1,880–2,800 m.

*Representative specimens:* COLOMBIA. **Cesar**: Mpio. Agustin Codazzi. Serrania de Perijá. Vereda Siete de Agost. Colecciones camino al páramo de Tres Tetas, desde la Cuchilla Macho Solo, zona de subparamo arbustivo con suelos arenoso pedregosos de color blanco, dominado por Asteraceae y Ericaceae, 9°57′03.0″-24.4″N, 73°02′04.3″-00′58.9″W, 1,884–2,551 m, 10 Dec. 2005, *O. Rivera Diaz & al. 2849* (COL!); Mpio. Serrania de Perijá. Vereda Siete de Agosto. Bosque arriba de la Cuchilla Macho Solo, dominado por *Toxicodendron, Oreopanax, Weinmannia, Bursera* con dosel antre 15 y 20 m, 9°57′09.0″-10.6″N, 73°1′45.9″-34.3″W, 1,898–2,200 m, 27 Feb. 2006, *O. Rivera Diaz & al. 3143* (COL!); **Cundinamarca**: Cordillera Oriental. Quebrada El Chico au N de Bogotá, 2,700–2,800 m, 30 Nov. 1952, *H. Humbert & al. 27544* (P!, UGDA!—drawing); Bogotá, La Mesa, 1,200 m, *J. Triana 623 p.p*. (P!) (Fig. 29).

*Notes:* While describing this species it was referred to *P. similis*, species widely distributed from Peru to Colombia. *Ponthieva cesarensis* and *P. similis* are easily separable by some floral characters—free lateral sepals found in *P. cesarensis*, versus lateral sepals connate almost to the apex in *P. similis*, cuneate base of petals in this species versus unguiculate base in its closest congener. Additionally, the lip callus is very prominent in *P. similis* and very obscure in *P. cesarensis*. It is also similar to both *P. villosa* and *P. crinita. Ponthieva cesarensis* can be easily distinguished from *P. villosa* by the form of lip which is oblong-obovate in outline (vs. triangular-ovate, with cuneate base), and unlobed, roof-like callus (vs. callus obscurely bilobed). Unlike *P. crinita* the lip of *P. cesarensis* is devoid of basal lip auricles. Both species have different form of lip either. Lip lamina is similar in form to *P. curvilabia* Garay, but unlike the latter species lip of *P. cesarensis* is subsessile, and lateral sepals have narrow, cuneate base.

*Ponthieva similis* C. Schweinf., Bot. Mus. Leafl., Harvard Univ. 9: 226, tab. 5, Figs. 5–7. 1941. TYPE: Peru, *J. Macbride 3395* (lectotype, designated by Garay (1978: 225), F-0041598F; isolectotype, AMES-00103564!; UGDA!—drawing).
*Ponthieva ochreata* Renz, Candollea 11: 271. 1948. TYPE: Colombia, *O. Renz 4132* (holotype, RENZ-4132.1!).

**Figure 34:**
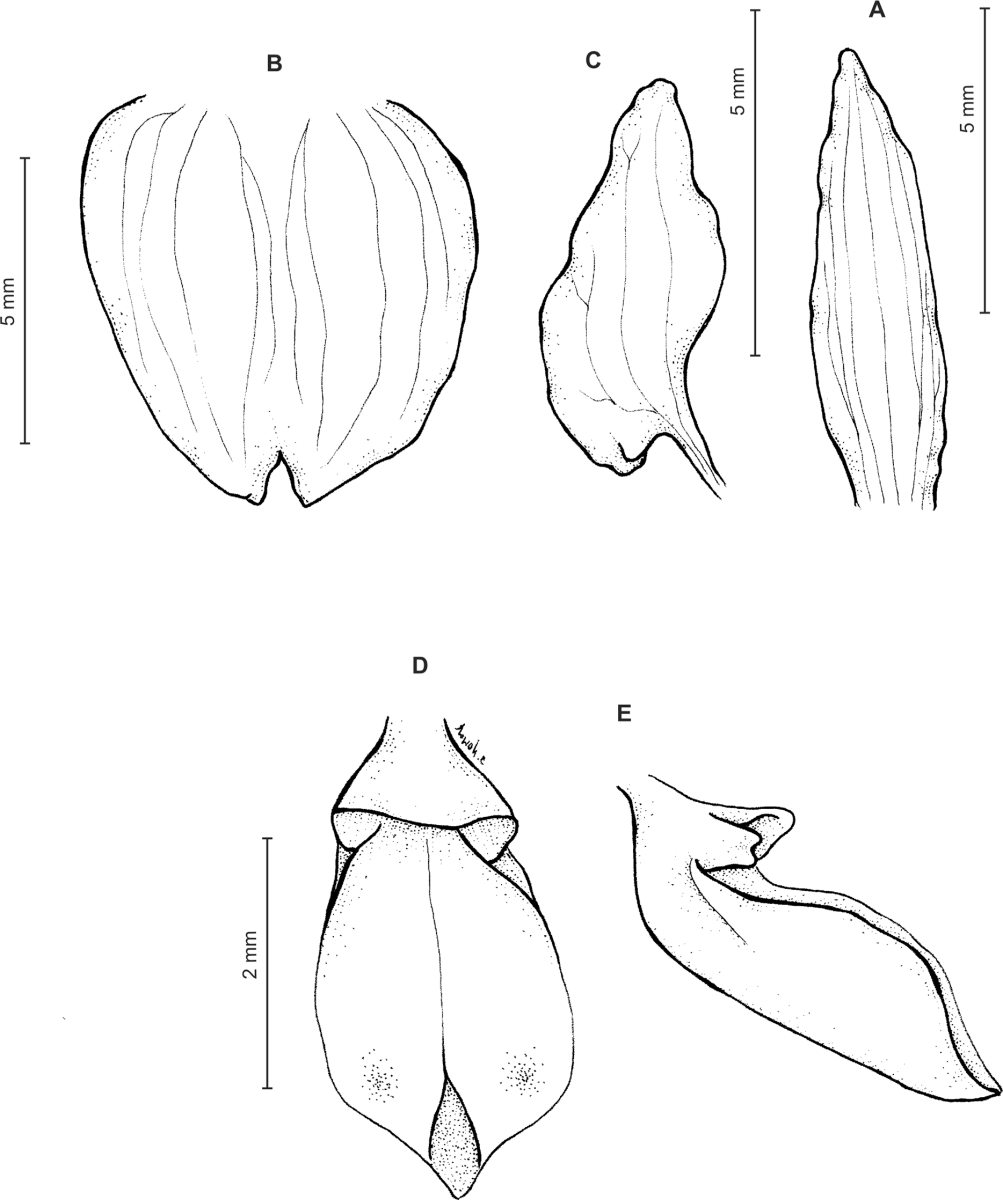
*Ponthieva similis* C. Schweinf. (A) Dorsal sepal, (B) lateral sepals, (C) petal, (D) lip, front view, (E) lip, lateral view. Drawn by N. Olędrzyńska from *Gonzalez 656*.

**Figure 35:**
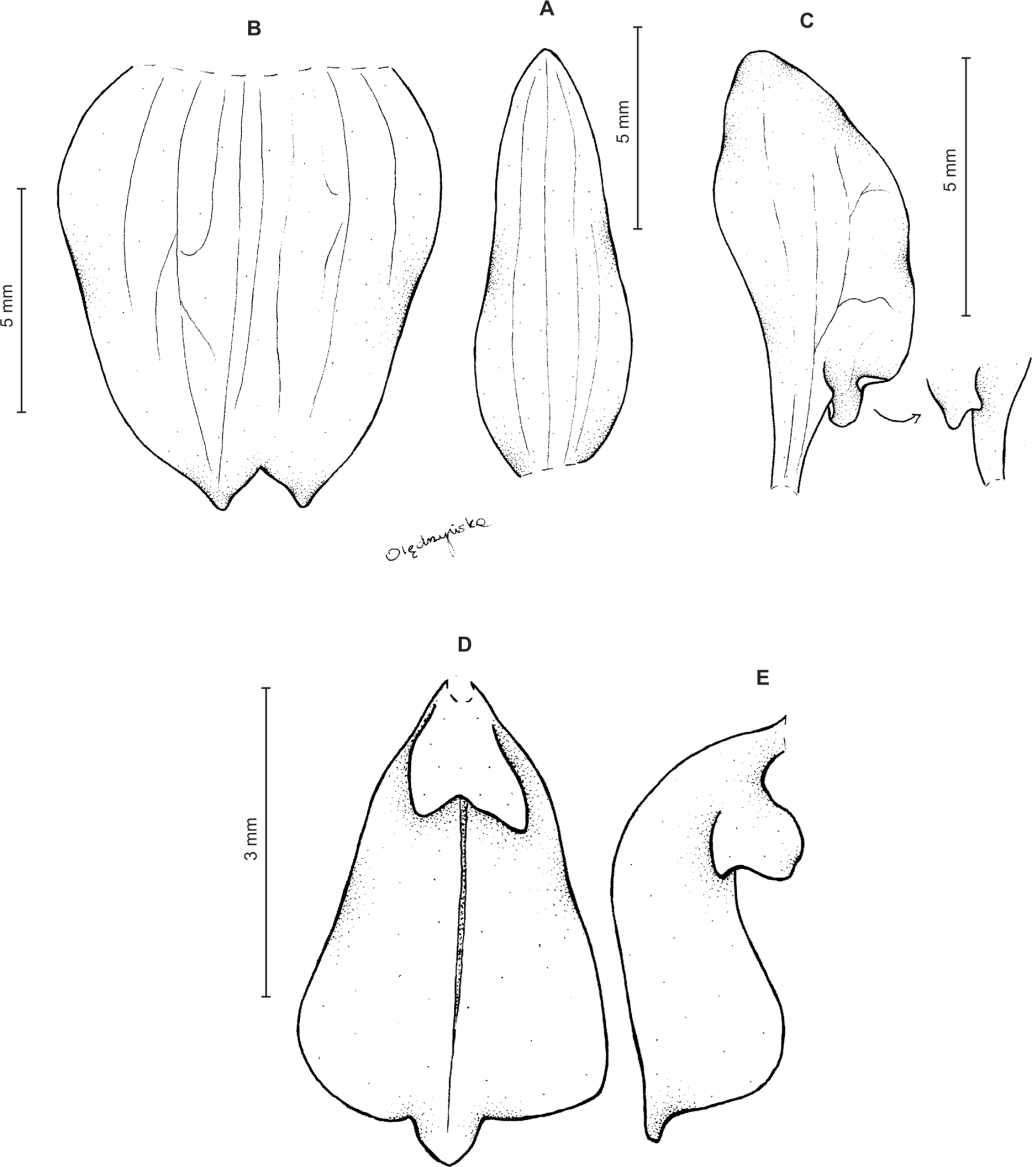
*Ponthieva similis* C. Schweinf. (A) Dorsal sepal, (B) lateral sepals, (C) petal, (D) lip, front view, (E) lip, lateral view. Drawn by N. Olędrzyńska from *Macbride 3395*.

Plants up to 60 cm tall. Leaves 2–9, basal, villose on both sides, petiolate; petiole up to 10 cm long, commonly shorter basally imbricating; blade up to 13 cm long and 3.5 cm wide, oblanceolate to elliptic-oblanceolate, abruptly acuminate, gradually tapering into a conduplicate petiole. Peduncle erect, pubescent throughout, remotely few-sheathed, terminated by a subdensely many-flowered raceme 4.5–8.2 cm long. Flowers glabrous, sepals white with green or brownish veins, lip white with two green spots on the disc, and pink to red at tip. Floral bracts up to eight mm long, ovate to ovate-lanceolate, acute to subacuminate, sparsely villose. Pedicellate ovary up to l0 mm long, cylindric, densely villose. Dorsal sepal up to 12 mm long and three mm wide, lanceolate to narrowly elliptic-lanceolate, acute to subacuminate, primarily 3-veined. Petals unguiculate, claw free part up to three mm long, linear; blade up to 8.5 mm long and four mm wide, obliquely elliptic, basal auricle provided with a short, lobe-like protuberance overlapping blade, veins 2, branching. Lateral sepals up to 10 mm long and nine mm wide in total, united into a cochleate synsepal, more or less orbicular or elliptic-ovate in outline, bidentate at apex, principally 10-veined. Lip subsessile, up to 4.5 mm long and three mm wide, fleshy, cymbiform to deeply concave, oblong obovate in outline, acute to obtuse in front, basally provided with a transverse, replicate plate, the sides of which are abruptly decurrent onto lip. Gynostemium ca three mm long, robust, subsessile (Figs. 34 and 35).

*Ecology*: Terrestrial. In Colombia flowering in May, June, July, August, and November.

*Distribution*: Peru, Ecuador, Colombia. Alt. 2,450–3,000 m.

*Representative specimens:* COLOMBIA. **Cundinamarca**: El Retiro—La Calera, 2,700 m, 18 Nov. 1938, *O. Renz 4132* (RENZ); Cordillera de Bogotá, 2,600–3,000 m, 19 Jul. 1943 & 8 May 1944, *M. Schneider 66* (AMES!, UGDA!—drawing); Mpio. de Sesquilé, zona de la Laguna Vieja de Guatavita, 2,900–3,000 m, 2 Jul. 2000, *J.L. Fernandez A. & J. Castillo 18916* (COL!, UGDA!—drawing); Mpio. de Chia. Vereda da Tiquiza, antigua quebrada, 2,650 m, Jul. 1996, *S. Cortes 733* (COL!); Carretera entre Subachoque y La Pradera. Vertiente oriental, 3,000 m, 24 Nov. 1956, *M. Ospina H. & J.M. Idrobo 58* (AMES!, COL!); Subachoque, Quebrada Matatigre, 2,750 m, 7 Aug. 1951, *H. Garcia Barriga 13622* (COL!); Alrededores de Bogotá, Quebrada de El Chico, 2,700 m, 29 Jun. 1951, *M. Schneider 66/2* (COL!); Al N de Bogotá, orillas de la Quebrada del Chico, 2,750–2,850 m, 8 Nov. 1941, *J.M. Gonzalez Sicard 125* (COL!); Usaquén, bosques densos y humedos, 2,700–2,900 m, 24 Jun. 1943, *M. Schneider 66/1* (COL!, UGDA!—drawing); Mpio. de Subachoque, Vereda Tobal. Inca El Cerro. En el interior de un bosque con presencia de *Weinmannia, Myrsine, Ilex, Miconia, Cavendishia, Ageratina*, 2,950 m, 14 May 2003, *M. Hernandez Schmidt 1142* (COL!); Bogotá, Localidad Usaquén. Ver. Torca, Calle 200. Frente estacion de policia, 2,650 m, 18 May 2000, *C.A.B. Gonzalez 656* (COL!, UGDA!—drawing); **Nariño**: Mpio. Pasto, Bosque de Dasa. Km 8 de la carretera al aeropuerto de Cano, 2,790 m, 22 May 1967, *G. López J. 097* (PSO!, UGDA!—drawing) (Fig. 29).

*Other materials examined:* PERU. Cani. Pueblo seven miles NE of Mito, 2,800 m, 16–26 Apr. 1923, *J. Macbride 3395* (AMES!, F). ECUADOR. **Azuay**: Under dense thorn scrub at lake Zarugucho, 20 km W of Cuenca, 2,800 m, 10 Jun. 1958, *C. Dodson 355* (RPSC!, UGDA!—drawing); **Zamora-Chinchipe**: S of Yangana above Valladolid, 2,450 m, 23 Mar. 1985, *C. Luer & al. 10883* (RPSC!, UGDA!—drawing).

Notes: The most distinguished character of this species is the petal form, especially its basal auricle, acute, falcate and overlapping blade.

**6. *Andicola* group**

Plants hirsute to pubescent, with basal leaves, relatively large and attractive flowers, usually with connate lateral sepals, unguiculate petals and small, unguiculate or sessile lip with or without any calli, gynostemium long or shortly stalked.

## Key to the Species

1. Petals with projection at base*P. inaudita*
1* Petals without any projection at base2
2. Lip ecallose*P. sylvicola*
2* Lip with callus3
3. Petals lamina prominently round-auriculate at base ... ***P. appendiculata***
3* Petals lamina with truncate base*P. andicola*

*Ponthieva andicola* Rchb.f., Linnaea 41: 52. 1877. TYPE: Ecuador, *R. Spruce 5998* (lectotype, designated by Garay (1978: 213), W!; isolectotypes, AMES-00112208!, B, F-0046472F!, G, GH-00112208, K-000573758).

*Ponthieva grandiflora* Ridl., Gard. Chron. 3: 264. 1888. TYPE: Ecuador, *F.C. Lehmann s.n*. (lectotype, designated by Garay (1978: 213), BM; isolectotype: AMES-00103530).

**Figure 36:**
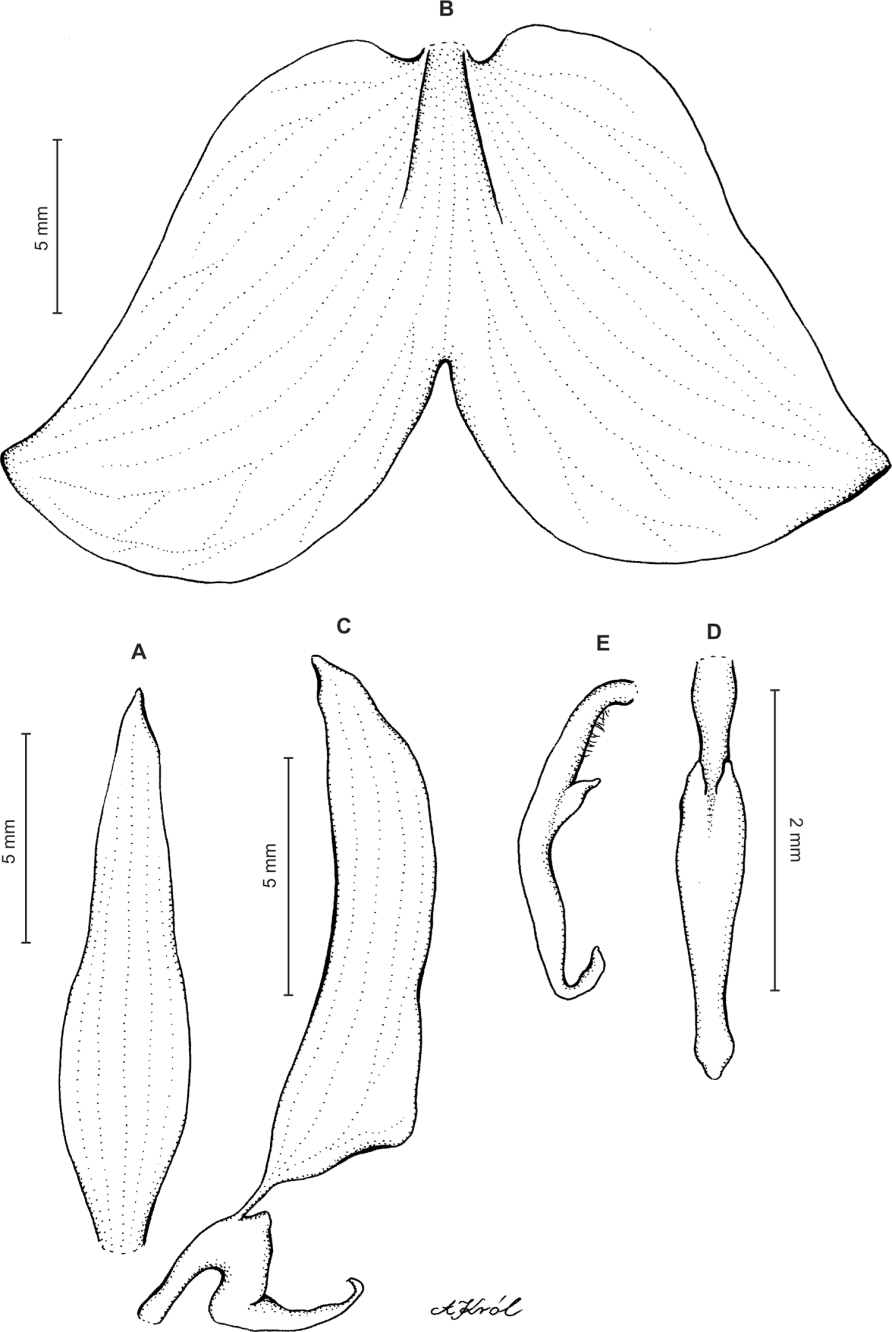
*Ponthieva andicola* Rchb.f. (A) Dorsal sepal, (B) lateral sepals, (C) petal and lip, (D) lip, front view, (E) lip, lateral view. Drawn by A. Król from *Lehmann s.n*.

Plants up to 35 cm tall. Leaves 1–3, basal, rosulate, subsessile, up to 20 cm long and 4.5 cm wide, elliptic to lanceolate-elliptic, acute to acuminate, hirsute throughout. Peduncle slender, villose, 2–3-sheathed, terminated by a loosely many-flowered raceme. Flowers large, glabrous, white with greenish stripes on sepals and brownish red stripes on petals. Floral bracts up to 10 mm long, ovate-lanceolate, acute to acuminate, villose. Pedicellate ovary up to 30 mm long, filiform, villose. Dorsal sepal 13–18 mm long, two to three mm wide, lanceolate, acute to acuminate, 5–9-veined. Petals unguiculate, claw free part ca three mm long; lamina 13–17 mm, 2.3–3 mm wide, ligulate to ligulate-triangular, with obtuse to rounded angles and cuneate base, veins 2, branching. Lateral sepals 13–20 mm long, 11–25 mm wide when spread, obliquely elliptic to elliptic-ovate, obtuse at apex, connate together to the middle or above, quadrate-flabellate in outline, concave at base. Lip unguiculate, claw ca one mm long, thickened, terete; lamina 2.5–3 mm long, 1–1.5 mm wide, linear-crescent-shaped from lateral view, linear-lanceolate, basally provided with a pair of conical to subglobose calli giving a subcordate appearance, apex acute. Gynostemium ca two mm long, long-stalked (Fig. 36).

*Ecology*: Terrestrial. Flowering in May and October.

*Distribution*: Ecuador, Colombia. Alt. 3,000 m. The occurrence of this species in Colombia was provided by Dodson (1992), Ortiz Valdivieso (1995), Ortiz Valdivieso & Uribe Vélez (2007), and Bernal, Gradstein & Celis (2015).

*Other materials examined*: ECUADOR. (**Azuay**): Pindelig bei Cuenca, *G. Wallis s.n*. (W!); Yerbabuena, W Andes of Cuenca. Oct. 1878. *F.C. Lehmann 7635* (AMES!, W!, UGDA!—drawing); **Cañar**: Near Tipococha on RR from Sibambi to Azogues, 3,000 m, 8 May 1958, *C. Dodson 352* (RPSC!, UGDA!—drawing); **Chimborazo**: 1857–1959, *R. Spruce 5998* (AMES!, B, G, K, W!); **Guayas**: Guayaquil, *E. Klaboch s.n*. (W!). *Sine loc., L. Schlim 5998* (G, RPSC!—photo).

Notes: The unique character of this species is its linear-lanceolate lip lamina, basally provided with a pair of conical to subglobose calli giving a subcordate appearance.

*Ponthieva appendiculata* Schltr., Repert. Spec. Nov. Regni Veg. 14: 116. 1915. TYPE: Ecuador, *A. Sodiro 141* (lectotype, designated by Garay (1978: 214), AMES-00103505!—drawing; isolectotype, QPLS).

**Figure 37:**
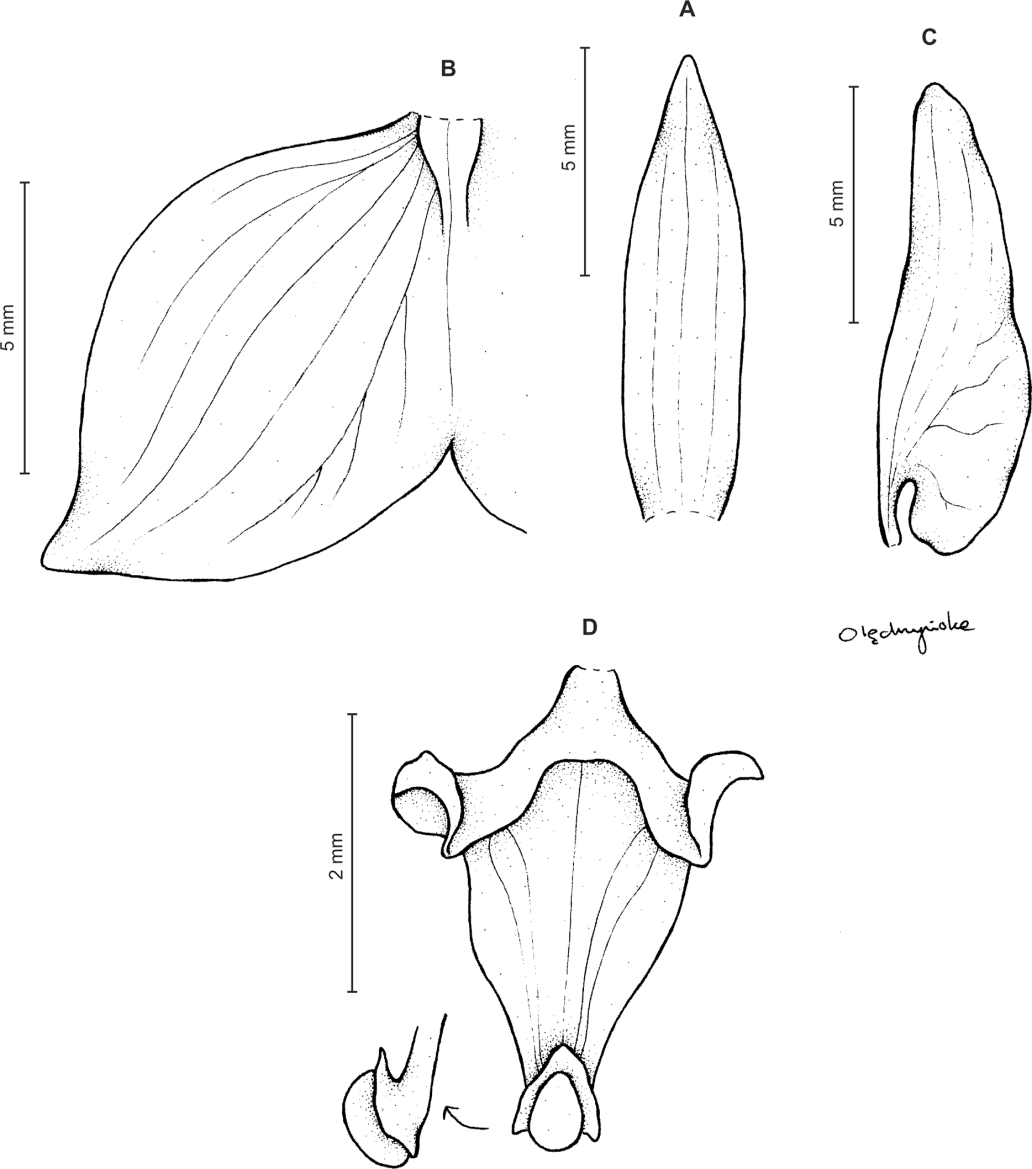
*Ponthieva appendiculata* Schltr. (A) Dorsal sepal, (B) lateral sepal, (C) petal, (D) lip, front view. Drawn by A. Król from *Hirtz 2069*.

Plants up to 20 cm tall. Leaves 1–3, basal, rosulate, subsessile to petiolate, petiole up to four cm long; lamina up to 16 cm long and 5.5 cm wide, oblanceolate, acute to subacuminate, gradually attenuate, villose throughout. Peduncle erect to somewhat arcuate, densely villose, without sheaths, terminated by a subdensely up to 10-flowered, raceme. Flowers greenish yellow, sepals rather sparsely pubescent-villose on the outer surface. Floral bracts up to 10 mm long, ovate-lanceolate, acuminate, villose. Pedicellate ovary up to 20 mm long, densely villose. Dorsal sepal 4–10.5 mm long, 2.3–3 mm wide, narrowly elliptic-lanceolate, acute, connivent with petals, 3-veined. Petals shortly unguiculate; claw ca 1–1.5 mm long; lamina up to 10 mm long and three mm wide, oblong-dolabriform to ligulate, obtuse, prominently round-auriculate at base, 2–3-veined. Lateral sepals up to 11 mm long and seven mm wide, broadly ovate, connate to middle, trapezoid in outline, truncate in front, inside sparsely papillose, multi-veined. Lip subsessile, up to four mm long in total, 2.5 mm wide, appressed against the column part, triangular in outline with a replicate base which is excised in the shape of reversed V, at the apex with a circular, cochleate callus, extending in a ligulate-cymbiform tip, basally with broadly spread, tubular lateral lobes. Gynostemium ca three mm long, massive, subsessile (Fig. 37).

*Ecology*: Terrestrial. Flowering in September and November.

*Distribution*: Ecuador, Colombia. Alt. 3,000 m. The occurrence of this species in Colombia was reported by Pedro Ortiz Valdivieso (1995).

*Other materials examined*: ECUADOR. **Pichincha**: Vulcan Pululahua, W of Quito, 3,000 m, 5 Nov. 1984, *A. Hirtz 2069* (MO!, RPSC!, UGDA!—drawing); In silvis occidentalibus montis Pieli, Sep. 1899, *A. Sodiro 141* (AMES!, QPLS).

*Notes*: *P. appendiculata* is easily separated from other representatives of the genus by peculiar lip form, which is triangular in outline with a replicate base. It is excised in the shape of a reversed V. The lip apex has a circular, cochleate callus, extending in a ligulate-cymbiform tip, and the base has broadly spread, tubular lateral lobes.

*Ponthieva sylvicola* Rchb.f., Linnaea 41: 52. 1877. TYPE: Ecuador, *W. Jameson s.n*. (lectotype, designated by Garay (1978: 226), W!).
*Ponthieva chuquiribambae* (Kraenzl.) Ames & C. Schweinf., Bot. Mus. Leafl., Harvard Univ.4(3): 38. 1936. *Pleurothallis chuquiribambae* Kraenzl., Ann. Naturhist. Mus. Wien. 44: 327. 1930. TYPE: Ecuador, *G. Wallis s.n*. (lectotype, designated by Garay (1978: 226), W-10882!, UGDA!—drawing).

**Figure 38:**
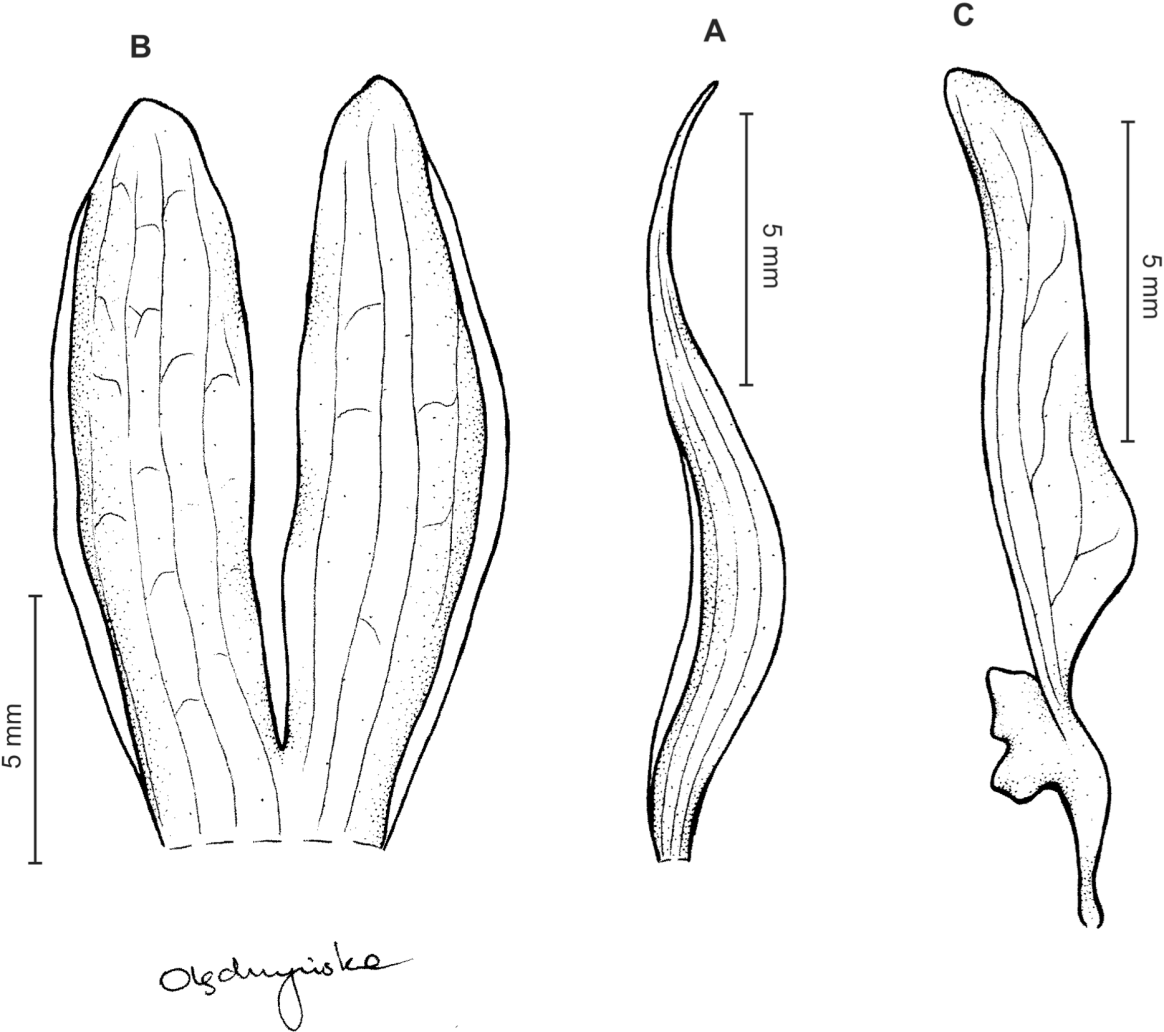
*Ponthieva sylvicola* Rchb.f. (A) Dorsal sepal, (B) lateral sepals, (C) petal and gynostemium. Drawn by N. Olędrzyńska from *Lehmann 340*.

**Figure 39:**
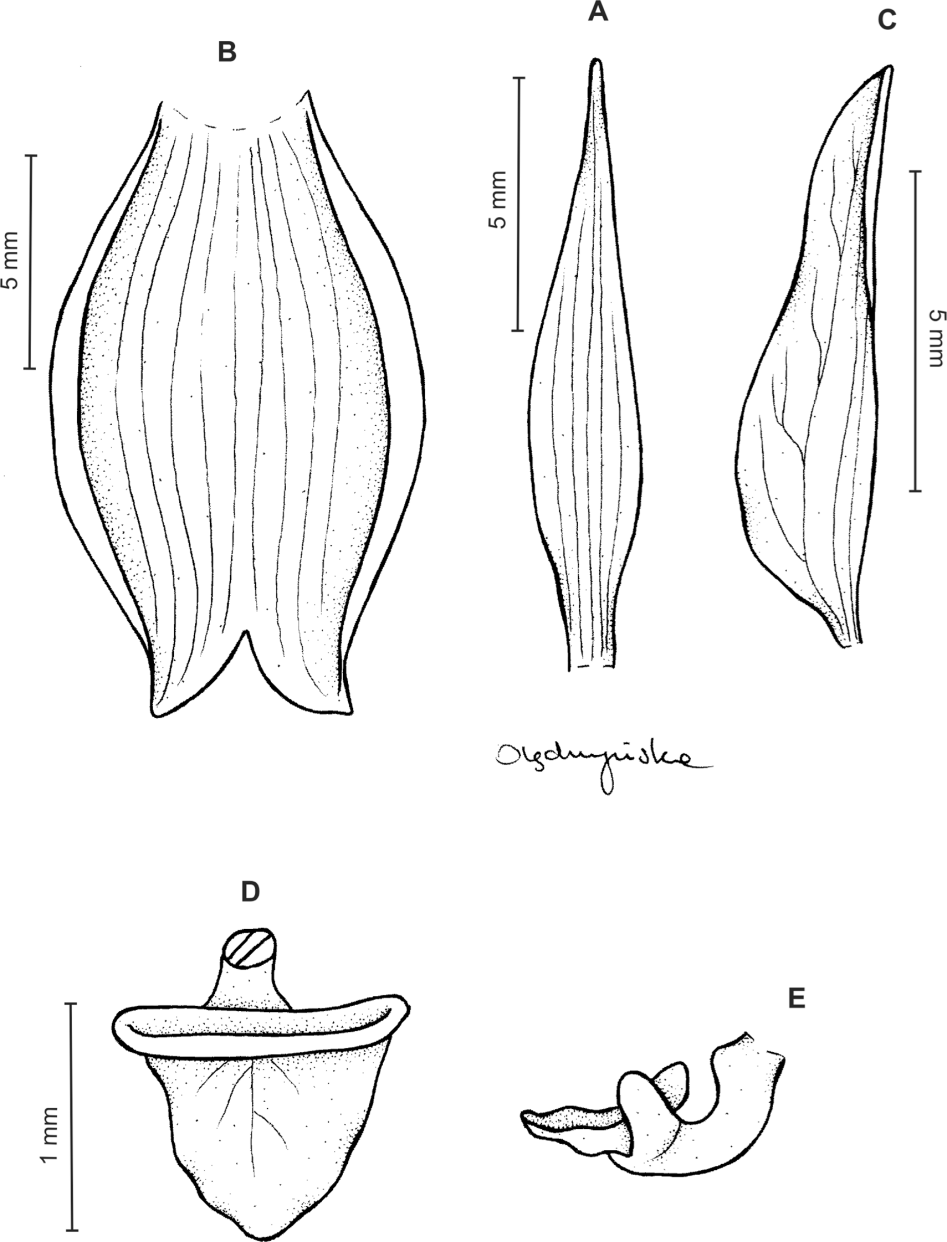
*Ponthieva sylvicola* Rchb.f. (A) Dorsal sepal, (B) lateral sepals, (C) petal, (D) lip, front view, (E) lip, side view. Drawn by N. Olędrzyńska from *Dodosn & Pozo 15469*.

Plants up to 60 cm tall. Leaves 5–6, basal, rosulate, petiolate; petiole up to 14 cm long, narrow, canaliculate; blade up to 15 cm long and five cm wide, elliptic-ovate to ovate, acute, rounded to cuneate at base, densely ciliolate-pubescent. Peduncle densely villose, with a single sheath in the middle, terminated by a densely to sublaxly many-flowered raceme. Flowers light green, lip white, sepals glabrous. Floral bracts 8 mm long, ovate-lanceolate, acute, villose. Pedicel and ovary 10–15 mm long, villose. Dorsal sepal 6–12 mm long, 1.5–3 mm wide, linear-lanceolate to oblong-ovate, acuminate, 5–7-veined. Petals subsessile to shortly unguiculate, 5.5–9 mm long, 1.5–2.2 mm wide, obliquely oblanceolate to lanceolate-elliptic, acute to acuminate, cuneate basally, spreading. Lateral sepals 9–14.5 mm long, up to 10 mm wide when spread, obliquely orbicular-ovate, rounded at apex, united nearly up to the apex to form a large, flat nearly rounded plate, with reflexed margins, and diverging apices, sometimes united only basally, multi-veined. Lip unguiculate; claw ca one mm long, upcurved, terete; lamina one mm long, 1.5 mm wide, horizontally placed, triangular-ovate, bilobed at the base, lobes connivent, the base developed into an elongate curved nectar tube inserted in the sepaline tube, the apex obliquely subobtuse. Gynostemium ca three mm long, long-stalked (Figs. 38 and 39).

*Ecology*: Terrestrial in wet upper montane forest. Flowering in February and November.

*Distribution*: Bolivia, Peru, Ecuador, Colombia. Alt. 2,000–2,900 m. The occurrence of this species in Peru was reported by Zelenko & Bermúdez (2009).

**Figure 40:**
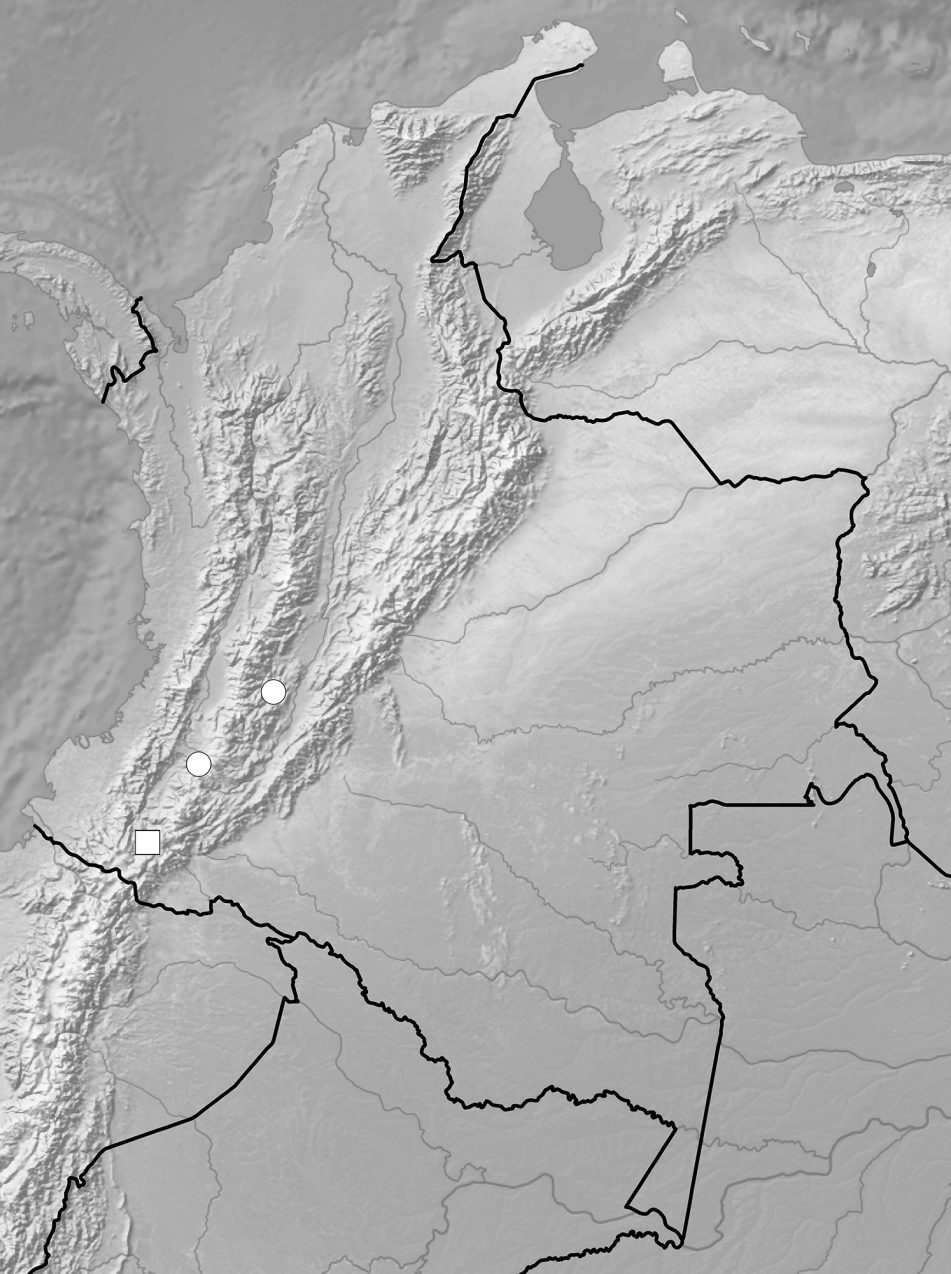
Distribution of representatives of *Andicola*-group. *P. inaudita* (circle), *P. sylvicola* (square). Map generated in ArcGis 9.3 (Esri, Redlands, CA, USA).

*Representative specimen:* COLOMBIA. Cordillera von Pasto, 2,000 m, 19 Feb. 1881, *F.C. Lehmann 340* (W!, AMES!—drawing) (Fig. 40).

*Other material examined*: ECUADOR. **Azuay**: Pindelig bei Cuenca, *G. Wallis s.n*. (W-R!, UGDA!—drawing); **Loja**: Chuquiribamba, *G. Wallis s.n*. (W!); […..], Cuenca-Las Cajas, 2,900 m, 28 Nov. 1984, *C. Dodosn & A. Pozo 15469* (RPSC!, UGDA!—drawing); Pichincha, *Sine loc.*, 1864, *W. Jameson s.n*. (W!).

*Notes*: According to Ames & Schweinfurth (1936) *P. chuquiribambae* is allied to the widespread *P. maculata* Lindl., particularly to the rather dwarf form of the plant found in Central America, but it differs in having apparently narrower connate lateral sepals and dissimilar distinctly unguiculate ovate-triangular lip.

*Ponthieva inaudita* Rchb.f., Linnaea 41: 18. 1876. TYPE: Peru, *J. Warszewicz s.n*. (lectotype, here designated, W!; AMES-00103536!—drawing; UGDA!—drawing).
*Ponthieva microglossa* Schltr., Repert. Spec. Nov. Regni Veg. Beih. 9: 56. 1921. TYPE: Peru, *E. Kohler s.n*. (lectotype, here designated, AMES-00103544!).

**Figure 41:**
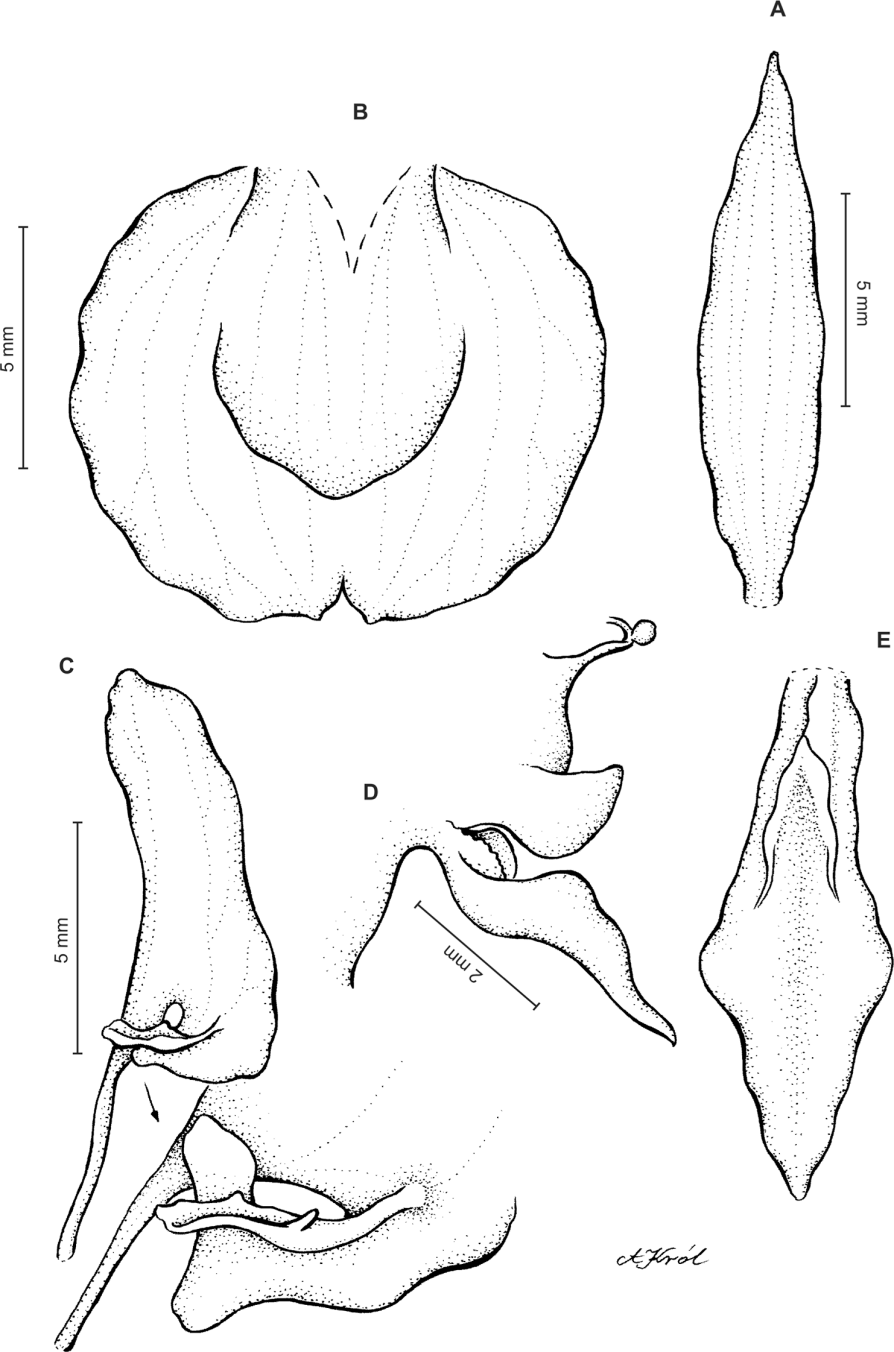
*Ponthieva inaudita* Rchb.f. (A) Dorsal sepal, (B) lateral sepals, (C) petal and lip, (D) lip and gynostemium, side view, (E) lip, front view. Drawn by A. Król from *Warszewicz s.n*.

Plants up to 35 cm tall. Leaves 2–5, basal, rosulate, petiolate; petiole four to five cm long; blade up to nine cm long and two cm wide, narrowly lanceolate, acute to acuminate, pubescent. Pedunce pubescent, 1–3-sheathed, terminated by a densely many-flowered raceme. Flowers medium-sized, sepals glabrous. Floral bracts seven mm long, oblong-lanceolate to lanceolate, acute, glandular. Pedicellate ovary 18 mm long, densely pubescent. Dorsal sepal 13 mm long, three mm wide, narrowly lanceolate to linear-lanceolate, acute, veins 5, simple. Petals unguiculate, claw free part five mm long, filiform; lamina eight mm long, 2.5–3 mm wide, oblong-ligulate to ligulate-subpandurate, somewhat falcate, obtuse, with hook-like, slightly twisted lobe at truncate base adorned with oblong, canaliculate callus with two teeth on both sides. Lateral sepals connate together almost completely forming almost orbicular lamina 11 mm long and wide, slightly notched at the apex, deeply concave in the center, veins numerous, distal ones branching. Lip simple, almost sessile, four mm long, two mm wide, narrowly sagittate, canaliculate, acute at the apex, with prominent keel at the base becoming furculate in front. Gynostemium ca four mm long, long-stalked (Fig. 41).

*Ecology*: Terrestrial or epiphytic in montane wet forest (Dodson & Vasquez, 1989). Flowering in February and November.

*Distribution*: Bolivia?, Peru, Ecuador?, Colombia. Alt. 2,300–2,500 m. The occurrence of this species in Ecuador was reported by Jørgensen & Leon-Yanez (1999) and from Bolivia by Jørgensen et al. (2014), however these authors included in *P. inaudita* also *P. koehleri* and *P. microglossa* Schltr. (Schlechter, 1921).

*Representative specimens:* COLOMBIA. **Cauca**: Hacienda Sotará, 2,300 m, 22 Feb. 1884, *F.C. Lehmann 3716* (G—Dueñas Gómez & Fernández Alonso, 2009); **Tolima**; La Ceja rio Ullnos, 2,500 m, 9 Nov. 1882, *F.C. Lehmann 2150* (G—Dueñas Gómez & Fernández Alonso, 2009) (Fig. 40).

*Other material examined*: PERU. *Sine loc., J. Warszewicz s.n*. (AMES!, W!).

*Notes*: The unique character of this species is the petal adorned with an oblong, canaliculate callus with two teeth on each side. This element is not shown in the illustration of *P. microglossa* deposited in AMES. As all other characters of this taxon correspond to *P. inaudita*, we follow previous concepts (Schweinfurth, 1958; Dodson & Vasquez, 1989) that the two species are conspecific.

*Incertae sedis:* We are not completely sure whether the species characterized below actually occurs in Colombia. The only examined material is deposited in W and it was collected by Moritz, probably in this country. More material is required to confirm its occurrence in Colombia.

*Ponthieva glandulosa* (Sims) R.Br., Hort. Kew., ed. 2, 5: 200. 1813. *Neottia glandulosa* Sims, Bot. Mag. 21: t. 842. 1805. TYPE: “West Indies,” *Anderson s.n*. (not localized).

**Figure 42:**
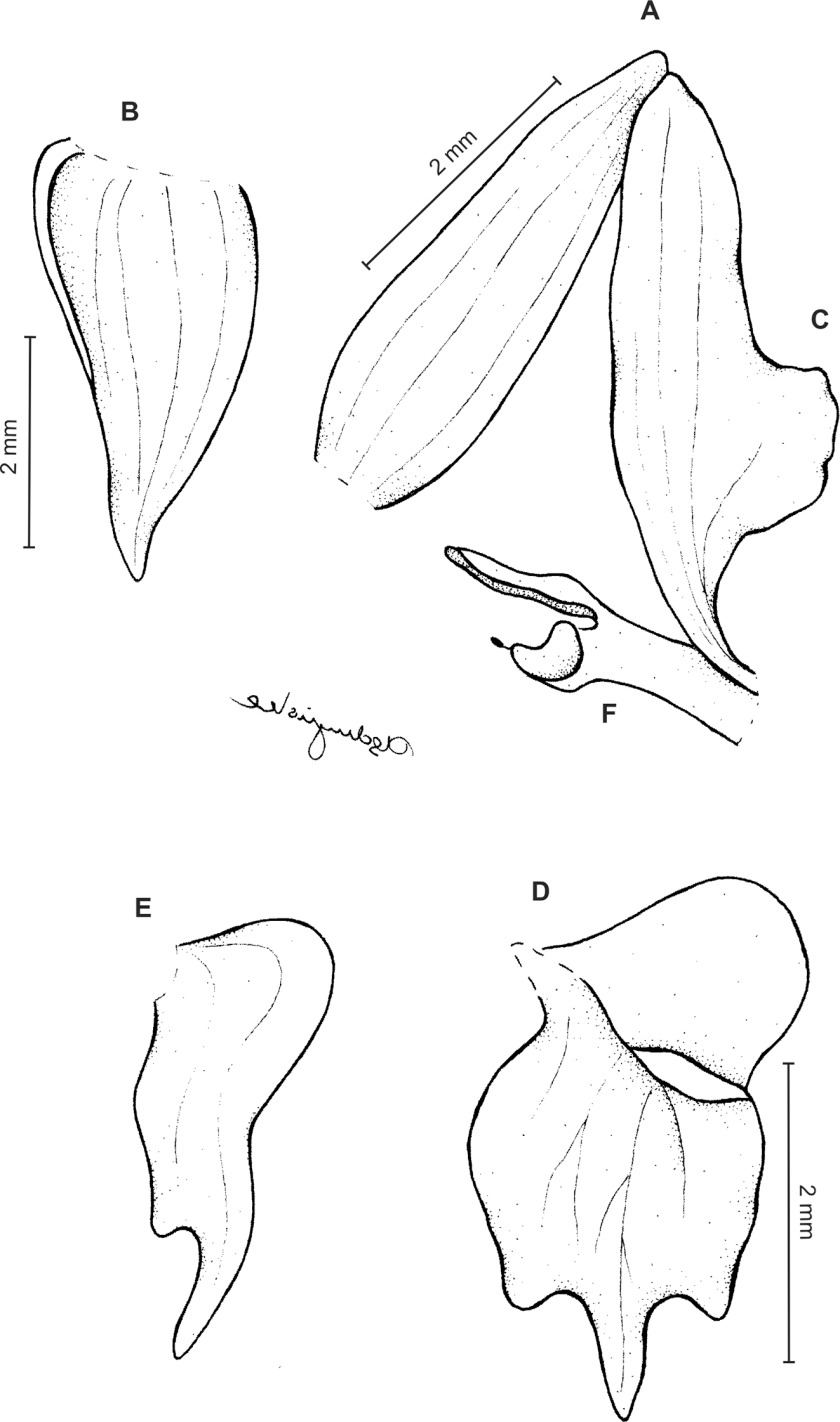
*Ponthieva glandulosa* (Sims) R.Br. (A) Dorsal sepal, (B) lateral sepal, (C) petal, (D) lip, front view, (E) lip, side view, (F) gynostemium. Drawn by N. Olędrzyńska from *Siutenis s.n*.

Plants 40 cm tall. Leaves 3–5, basal, rosulate, shortly petiolate; petiole up to two cm long; blade up to 10 mm long and two cm wide, lanceolate to oblong lanceolate, acute to acuminate, the uppermost above the base of the stem, ovate, sessile. Peduncle erect, covered by several, partially imbricating sheaths, terminated by a loosely many-flowered raceme about 20 cm long. Floral bracts 10 mm long, ovate-lanceolate, acuminate, glandular-hairy. Pedicellate ovary 13 mm long, glandular-hairy. Dorsal sepal six to seven mm long, two mm wide, oblanceolate to linear-oblanceolate, acute to subobtuse, 3-veined, lateral nerves branching. Petals unguiculate; claw connivent to the column part; lamina five mm long, four mm wide, obliquely triangular, with both ends rounded to blunt, nerves branching from the base of the free part. Lateral sepals six to seven mm long, three mm wide, ovate-elliptic to ovate-lanceolate, apically falcate, obtuse, nerves 5, branching and anastomozing. Lip unguiculate; claw connate with the column part; disc five mm long and wide, transversely elliptic-cordate, concave in the center, with deltoid or sagittate, blunt apiculus. Gynostemium 2.6 mm long in total, long-stalked (Fig. 42).

*Ecology*: No data.

*Distribution*: Colombia, Cuba, Puerto Rico.

*Representative specimens:* COLOMBIA. **Antioquia**: *Sine loc*. (Schlechter, 1920: 216); **Cauca**: *Sine loc*. (Schlechter, 1920: 216); *Sine loc. J.W.K. Moritz s.n*. (W!).

*Other materials examined:* CUBA. *Sine loc.*, 1824, *E. Poeppig s.n*. (W!). PUERTO RICO. *Siutenis s.n*. (AMES!).

*Notes*: This species was considered as conspecific with *P. racemosa* (Walter) C. Mohr by several researchers (D’Arcy, 1987; Bogarín et al., 2014); however, both species differ in flower morphology. In *P. glandulosa* petals are unguiculate, glabrous on the margin and the lip apical part is deltoid or sagittate.

## Additional Information and Declarations

### New Species Registration

The following information was supplied regarding the registration of a newly described species: Ponthieva vallecaucana Szlach. Kolan. & Olędrzyńska LSID 77196150-1.

### Data Availability

The following information was supplied regarding data availability: Herbarium specimens are deposited in herbaria AMES, BM, CUVC, COL, E, F, FI, K, MO, NY, QCA, QCNE, P, PSO, RPSC, UGDA, US, USF, VALLE, and W. The information about specimen location is indicated for each specimen in the article. There are no accession codes—the collection nr is the only recognition method of each specimen and this is stated for each examined specimen. The research in this article did not generate any data or code. All relevant data are presented in the manuscript. List of herbaria where cited examined specimens are deposited is provided in the ‘Materials and Methods’ section of the paper.
